# Rod-shape theranostic nanoparticles facilitate antiretroviral drug biodistribution and activity in human immunodeficiency virus susceptible cells and tissues

**DOI:** 10.7150/thno.39847

**Published:** 2020-01-01

**Authors:** Bhavesh D. Kevadiya, Brendan Ottemann, Insiya Z. Mukadam, Laura Castellanos, Kristen Sikora, James R. Hilaire, Jatin Machhi, Jonathan Herskovitz, Dhruvkumar Soni, Mahmudul Hasan, Wenting Zhang, Sarella Anandakumar, Jered Garrison, JoEllyn McMillan, Benson Edagwa, R. Lee Mosley, Richard W. Vachet, Howard E. Gendelman

**Affiliations:** 1 Department of Pharmacology and Experimental Neuroscience, College of Medicine, University of Nebraska Medical Center, NE, USA; 2 Department of Pharmaceutical Sciences, College of Pharmacy, University of Nebraska Medical Center, NE, USA; 3 Department of Chemistry, University of Massachusetts, Amherst, MA, USA; 4 Department of Pathology and Microbiology, College of Medicine, University of Nebraska Medical Center, NE, USA; 5 Nebraska Center for Materials and Nanoscience, University of Nebraska-Lincoln, NE, USA

**Keywords:** Long acting antiretroviral therapy, Laser ablation inductively coupled plasma mass spectrometry, Single photon emission computed tomography, Drug biodistribution, Multimodal imaging

## Abstract

Human immunodeficiency virus theranostics facilitates the development of long acting (LA) antiretroviral drugs (ARVs) by defining drug-particle cell depots. Optimal drug formulations are made possible based on precise particle composition, structure, shape and size. Through the creation of rod-shaped particles of defined sizes reflective of native LA drugs, theranostic probes can be deployed to measure particle-cell and tissue biodistribution, antiretroviral activities and drug retention.

**Methods**: Herein, we created multimodal rilpivirine (RPV) ^177^lutetium labeled bismuth sulfide nanorods (^177^LuBSNRs) then evaluated their structure, morphology, configuration, chemical composition, biological responses and adverse reactions. Particle biodistribution was analyzed by single photon emission computed tomography (SPECT/CT) and laser ablation inductively coupled plasma mass spectrometry (LA-ICP-MS) imaging.

**Results**: Nanoformulated RPV and BSNRs-RPV particles showed comparable physicochemical and cell biological properties. Drug-particle pharmacokinetics (PK) and biodistribution in lymphoid tissue macrophages proved equivalent, one with the other. Rapid particle uptake and tissue distribution were observed, without adverse reactions, in primary blood-derived and tissue macrophages. The latter was seen within the marginal zones of spleen.

**Conclusions**: These data, taken together, support the use of ^177^LuBSNRs as theranostic probes as a rapid assessment tool for PK LA ARV measurements.

## Introduction

Current management of human immunodeficiency virus type one (HIV-1) infection has transformed clinical outcomes from a life-threatening disease to a chronic manageable medical condition.^1^, ^2^ Nonetheless, required lifelong daily dosing of combination antiretroviral therapy (ART) has limitations for regimen adherence and in pill fatigue.^3^, ^4^ Recently the prospect of broad dissemination of antiretroviral drugs (ARVs) to virus-infected and susceptible people has led to the notion that improved therapeutic regimen adherence could eliminate the acquired immunodeficiency (AIDS) epidemic.^5^, ^6^ However, complete elimination requires more effective clinical outcomes for both pre-exposure prophylaxis and treatment regimens.^7^ Moreover, recent studies suggest that total HIV-1 elimination could be achieved by excision of integrated HIV-1 DNA or through the use of broadly neutralizing antibodies. However, these treatments are years from human use, which underscores the immediate need for better antiretroviral regimens.^8^-^11^

One pathway forward to affect patient treatment outcomes is by long-acting (LA) ARV injectables.^12^-^15^ These new deliverables hold considerable promise towards improving patient adherence and affecting reduction of viral reservoirs. This is made possible by changing pharmacological management to affect drug efficacy. Such approaches eliminate the requirements for lifelong daily pills. Nonetheless, a number of challenges exist to implement such regimens. These include, but are not limited to, required dosing volumes, frequencies of injection, untoward side effects, and maintenance of antiretroviral efficacy.^16^-^18^ Even more importantly, only two LA ARVs are currently in the pipeline.^12^ Such limitations in drug choices preclude alternative therapies when required by either developed resistance mutations or adverse events. To these ends, our laboratories have employed ARV chemical modifications to create novel LA prodrug libraries designed to extend drug half-life and enhance bioavailability.^13^, ^14^, ^19^-^22^ Notwithstanding, these novel prodrugs ensure drug penetration into “putative” viral reservoirs and sustain maximal antiretroviral activities.^15^, ^23^ We posit that development can be accelerated by modeling drug delivery, pharmacokinetics (PK), and biodistribution (BD).

HIV theranostics, if appropriately implemented, would accelerate the development of LA ARVs by predicting real-time drug biodistribution and potency.^24^, ^25^ The creation of theranostic probes would serve to speed bench to bedside research in the development of the most effective LA ARV nanoformulations. Thus, we sought to engineer nanoparticles that mimicked LA ARV particles in shape and morphology while optimizing size and design. To these ends, the use of bismuth (Bi) and sulfur (S) proved to be a natural choice as the components add a heavy metal detector, proven bioimaging capabilities and rapid oxidative decompositions required for human translation.^26^, ^27^ Intrinsically-radiolabeled ^177^lutetium (^177^Lu) bismuth sulfide nanorods (^177^LuBSNRs) were created as multimodal imaging particles to serve as probes for drug biodistribution of LA nanoformulated rilpivirine (NRPV). RPV, a non-nucleoside reverse transcriptase inhibitor (NNRTI), was chosen as a model for other LA ARVs based on its highly hydrophobic and protein binding properties as well as extended plasma half-life.^24^ We used ^177^LuBSNRs as imaging probes to mirror the structure, morphology, biological properties and atomic configuration of the ^177^LuBSNRs of LA NRPV. Biodistribution of ^177^Lu/BSNRs/RPV into lymphoid tissue macrophages was demonstrated in Balb/c mice using single photon emission computed tomography (SPECT) and laser ablation inductively coupled plasma mass spectrometry (LA-ICP-MS). The BSNRs/RPV particles demonstrated potent antiretroviral activities in cell-based assays. These data, taken together, demonstrate that these multimodal particles can be effective imaging probes for assessing LA NRPV biodistribution. The work demonstrates their translational application for determining LA ARV PK and pharmacodynamics (PD).

## Results

### Creation of ^177^LuBSNRs particles

**Figure 1:**
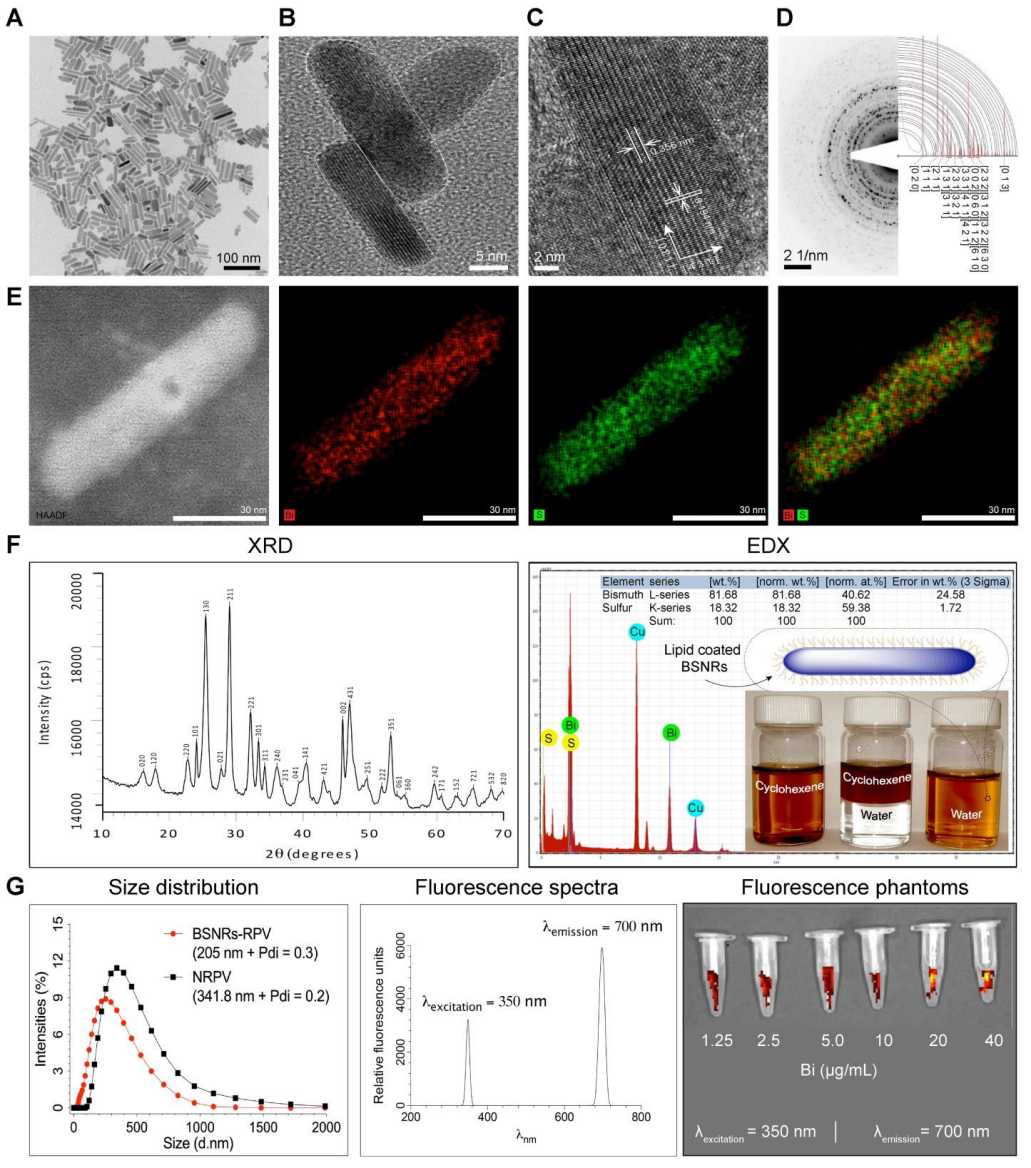
** Synthesis and characterization of multimodal particles.** Uniform rod shaped BSNRs were made by a modified solvothermal method in the presence of oleylamine and oleic acid.^26^, ^28^, ^29^ Oleylamine was the solvent for thioacetamide in forming the BSNRs (**Supplementary Figure S1**). Low-resolution TEM images of BSNRs are illustrated in (A). The BSNRs dimensions were approximately 12 nm in diameter by 70 nm in length. High-resolution TEM images of twin BSNR particles with lattice planes are shown in (B) with corresponding electron diffraction patterns. These are illustrated through the drawing of [4 3 1] and [1 3 0] space groups in the vector directions (C). The selected area electron diffraction (SAED) pattern of BSNRs are shown by simulation indexation. The indexation-detected lines correspond to characteristic interplanar spacing of the BSNR's single crystal structure in (D). The bismuth (red) and sulfur (green) element mapping showed element localization within the particles by corresponding high-angle annular dark-field electron microscopy in (E). XRD patterns, correlations with HR- TEM, SAED and simulated data sets confirmed the particle's structural configurations (F left panel). Quantitative measurements of particle element distribution were made by energy dispersive X-ray spectroscopy (F right panel). The hydrophobic and hydrophilic properties of the lipid coated particles are shown in the inset. The vial BSNRs were dispersed in cyclohexane. The vials contain mixtures of cyclohexane. The third vial shown illustrates the BSNRs particle hydrophilic properties produced by lipid coatings. These were dispersed in water. The schematic diagram of the particle's configuration is shown on the vial top. The hydrodynamic size distribution of lipid-coated drug loaded particles as determined by DLS was compared against NRPV (G left panel). Excitation and emission spectra of BSNRs (g middle panel) are illustrated. BSNRs NIR fluorescence imaging is shown (G right panel). Chemical composition of BSNRs was assessed by X-ray fluorescence and demonstrated the following; Bismuth = 88.61 mass% and sulfur = 11.38 mass% contents (**Supplementary figure S3**)

Chelator-free, ^177^LuBSNRs nanocrystals were first synthesized by solvothermal precipitation of bismuth neodecanoate and thioacetamide in the presence of oleic acid, oleylamine and ethanol. The crystallinity, monodispersity, shape, and size of the particles were monitored during synthesis, and optimized by varying time intervals and molar ratios of bismuth and thiol precursors. During synthesis, oleyl amine served as ligand and solvent with bismuth.^28^ The prepared ^177^LuBSNRs were dispersed in cyclohexane and converted to a freely dispersed hydrophilic solution in the aqueous phase. To ensure biocompatibility, the hydrophobic ^177^LuBSNRs particles were coated with biocompatible lipids. Lipid coated ^177^LuBSNRs were synthesized by a simple film hydration technique to make uniform preparations more appropriate for biological use than uncoated hydrophobic particles. L-α-phosphatidylcholine and DSPE-PEG_2000_ were used to functionalize the particles. An experimental outline is presented in the schematics diagram (**Supplementary Figure S1 top panel**). The plain, non-lipid coated and non-radiolabeled BSNRs were characterized by various techniques. Low power TEM revealed that the plain non-radiolabeled BSNRs nanocrystals had a rod shape, of approximately 12 nm in diameter by 70 nm in length **(Figure 1A)**. High resolution (HR)-TEM images **(Figure 1B-C)** show the lattice fringes with spacings of 0.356 and 0.194 nm. These are in accord with spacing planes of [130] and [431] of the atomic configuration of BSNRs. The selected area electron diffraction (SAED) patterns of the BSNRs are shown by simulation indexation (**Figure 1D**). The lines correspond to the rod's interplanar spacing.^29^ High-angle annular dark-field scanning TEM (HAADF-STEM) illustrate bismuth (Bi) (red) and sulfur (green) (**Figure 1E**). The XRD shown in **Figure 1F** reveals that all peaks are indexed into an orthorhombic crystal (JCPDS no. 01-089-8963), indicating high quality of crystallization. The XRD patterns of the BSNRs match with reported values in the literature.^30^-^32^ BSNRs present peaks that are assignable to the orthorhombic structure (JCPDS no. 01-089-8963). BSNRs are elongate along the [130] direction, representing the crystal plane of the longitudinal direction **(Figure 1C)**. The energy dispersive X-ray spectroscopy (EDX) analysis demonstrated “Bismuth” and “sulfur” signals were detected in the BSNRs; whereas the copper peaks originated from the TEM grid **(Figure 1F)**. The percent metal ions distributed in single particles are 81.68 and 18.32 weight percent of bismuth and sulfur, respectively, as seen in the inset of **Figure 1F** (Right top panel) and these results were similar to X-ray fluorescence (XRF) analysis results **(Supplementary figure S3)**. The mean hydrodynamic size of BSNRs by laser-light scattering is illustrated in **Figure 1G**. The size is ~ 205 nm and equivalent to that of NRPV **(Table 1) (Supplementary Figure S1).**^33^ Phantom near infrared (NIR) fluorescence images of particles are shown in **Figure 1G**. Synthesis and characterization results indicate that ^177^LuBSNRs could be employed to design a potential multi-modal theranostics agent that integrates SPECT and NIR imaging for a real-time non-invasive visualization of the virus sanctuary sites, which may provide more complementary, effective, and accurate information on progress of HIV-I spread with delivery drugs at the sanctuary sites. Non-radioactive LuBSNRs and BSNRs-RPV particles synthesis and characterization results were similar to that of radioactive particles **(Supplementary SR1 and figure S2, S4-5)** and **Table 1.**

### Radiolabeling stability assessment

In order to accurately localize our nanoparticles by SPECT imaging, we verified that the radiolabel remained stably associated within the nanoparticle to preclude false positive signals in the SPECT scans that would result if the radiolabel dissociates from the particle. We developed a process of intrinsically doping ^177^Lu atoms into the crystal lattice structure (formed by regular repeating units of bismuth and sulfur) of the BSNRs, creating a unique radiolabel nanoprobe for *in vivo* studies. The stability results are summarized in **Table 2**. ^177^LuBSNRs were incubated for 3 days at 4ºC or 37ºC in either PBS (pH 7.4) or rat plasma. Every 24 hours, the percent radioactivity left in the nanoparticles was calculated. Over the course of 72 hours in PBS at 4ºC or 37ºC, nanoparticles lost approximately 30% of their radioactivity, with 72% and 66% remaining, respectively. In 4ºC and 37ºC plasma, particles lost approximately 50% radioactivity, with 51% and 56%, remaining, respectively. ^177^BSNRs particles lost approximately 30% of their radioactivity when incubated in PBS for 72 hours. This can be attributed to the fact that some sample is lost (about 10-15%) each time the nanoparticles are pipetted, as the BSNRs particles “stick” to the inside of the pipette tip during these experiments. The greater loss of radioactivity in plasma can be attributed to the association of BSNRs with lipids and lipoproteins present in the rat plasma. We believe this to be the most likely explanation because we intrinsically doped the BSNRs particles. In this system the ^177^Lu ions exist in a tight coordination with bismuth and sulfur atoms within the core of nanoparticle which is shielded by an external lipid layer.^33^ These nanoparticles, along with other lipid coated nanoparticles are known to form associations with lipids and proteins both *in vivo* and when incubated in plasma *in vitro*. These lipids and lipoproteins do not pellet when subject to the centrifugation applied in this experiment and instead remain floating atop the supernatant. Typically, lipids and lipoproteins will not pellet unless subjected to ultracentrifugation.^34^ This leads to a higher reading in the supernatant (due to floating, lipid bound BSNRs particles) and a lower reading in the pellet, potentially masking the true stability of the radiolabel to the particle. Overall, these results demonstrate that ^177^Lu-labeled particles are stable, with half-lives that mirror the expected decay of ^177^Lu intrinsically doped within nanoparticle cores.

### MDM particle uptake, retention and release assessments

**Figure 2:**
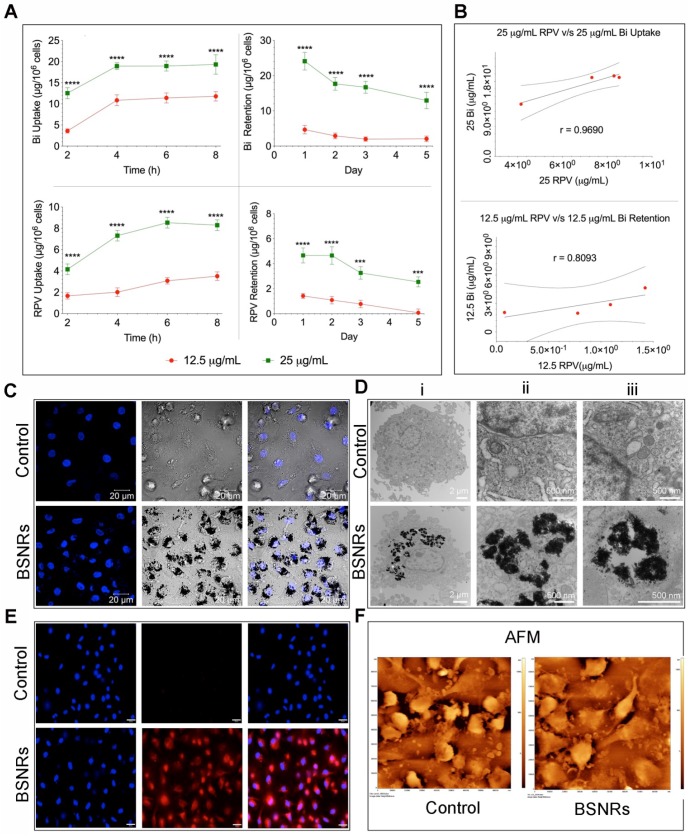
** Human macrophage particle uptake and retention.** Human monocyte-derived macrophage uptake and retention of lipid-coated RPV loaded BSNRs were determined at two treatment concentrations (12.5 and 25 µg/mL as bismuth content) (A). Bismuth and RPV content were determined by ICP-MS and UPLC-MS/MS analysis, respectively. Pearson's correlations of average bismuth and RPV content over time for both uptake and retention were determined (B). These served as a cross-validation of the drug loaded particle integrity in cells over the time periods used for analysis, presented in** supplementary figure S9)**. To qualitatively determine cell uptake, macrophages were treated with 25 µg/mL of the BSNRs particles (25 µg/mL) for 8 hours and confocal (C), TEM (D) and NIR (E) cell images were obtained. The surface morphology of particle-loaded cells was assessed by AFM (F). Statistical differences were determined using two-way ANOVA between groups with a Bonferroni's post hoc-test to correct for multiple comparisons. ***p < 0.001; ****p < 0.0001.

Time-dependent uptake of the BSNRs-RPV particles at 12.5 and 25 µg/mL by human monocyte-derived macrophages (MDM) was observed over 8 hours** (Figure 2A left panels).** As expected, treatment with 25 μg/mL of BSNRs-RPV provided increased particle uptake compared to the 12.5 μg/mL treatment. Intracellular bismuth and RPV levels were 11.78 μg/10^6^ cells for bismuth and 3.51 μg/10^6^ cells for RPV with the lower concentration treatment and 19.32 μg/10^6^ for bismuth and 8.28 μg/10^6^ cells for RPV with the higher concentration treatment at 8 hours. With particles left in the cell culture media, the cellular uptake reaches equilibrium within 8 hours. In retention studies, removal of particles from the media led to gradual diminution of intracellular particles **(Figure 2A right panels).** The amount of bismuth and RPV remaining in MDM treated with 25 μg/mL of particles after five days was 51.5% and 54.5%, respectively. Fluorescence characteristics of BSNRs-RPV and BSNRs particles in cells were assessed by measuring the relative fluorescence emission intensity in MDM suspended in PBS. Fluorescence spectra of BSNRs-RPV in MDM showed a higher intensity compared to BSNRs. However, both particles were taken up in MDM, and fluorescence intensity correlated with the concentration of particles **(Supplementary Figure S10D).** We hypothesized that with treatment with BSNRs-RPV particles, uptake and retention of bismuth in MDM would correlate with the amount of drug (RPV) taken up and retained by the cells. **Figure 2B** shows the two-parameter Pearson's correlation between bismuth and RPV cell concentration for uptake (top) and retention (bottom). Comparisons were performed at each time point and the uptake of bismuth positively correlated with the uptake of RPV, with an “r” value of 0.9690. For retention, the correlation between bismuth and RPV was lower (r = 0.8093), most likely due to the longer period of time over which the cells were analyzed (details for the *in vitro* correlation study are available in the **supplementary results SR3 and Figure S9**).

To qualitatively examine the intracellular uptake of BSNRs-RPV particles and the microstructure of the cells, we added 25 μg/mL of BSNRs-RPV particles to MDM for 8 hours, collected the cells and fixed them in a fixative buffer.^24^, ^35^ Nanoparticle loaded cells were examined by confocal microscopy (**Figure 2C**), TEM (**Figure 2D**), and validated by NIR fluorescence using NIR microscopy **(Figure 2E).** TEM images show BSNR particles endocytosed by MDM with higher magnification showing large numbers of BSNRs-RPV particles in vesicles, observed as black clusters distributed within lysosomes and other microvesicles (**Figure 2D**). These results were confirmed by May-Grünwald Giemsa staining that showed particles in the cells clearly visible by light microscopy **(Supplementary Figure S10A-C).** We assessed the surface topography of treated cells by AFM to determine characteristic morphological changes compared to control cells. BSNRs treated macrophages displayed a surface pseudopodium like morphology compared to control cells. AFM results demonstrated that particles were taken up in the cells with a slight change in cell surface morphology (**Figure 2F**).

**Figure 3:**
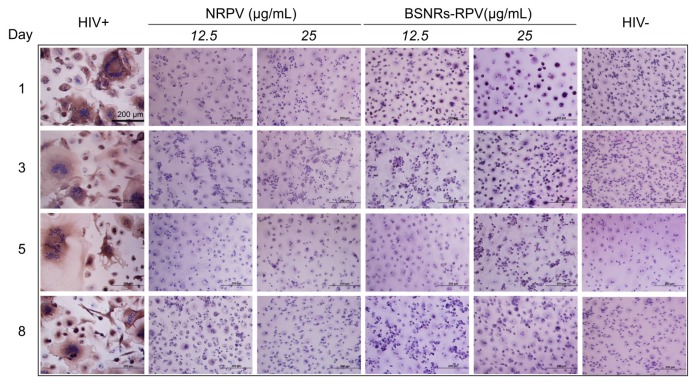
** Antiretroviral activities.** Antiretroviral activity was determined in macrophages treated for 8 hours with NRPV and BSNRs-RPV particles (12.5 and 25 µg/mL), and then infected with HIV-1_ADA_ at a multiplicity of infection (MOI) of 0.1 infectious particles per cell at days 1, 3, 5, or 8 after drug loading. At 10 days after infection, progeny HIV virion production was determined by P24 staining of cells. Antiretroviral activity was comparable for BSNRs-RPV and NRPV. Results for associated HIV RT activity in cell culture medium are available in **supplementary figure S11.**

Antiretroviral activity was assessed in MDM to determine the activity of BSNRs-RPV and NRPV particles. Cells were pre-treated with 12 or 25 μg/mL of NRPV and BSNRs-RPV for 8 hours before being challenged with HIV-1_ADA_, a macrophage tropic strain, at days 1, 3, 5 and 8 after treatment. Ten days after infection, culture supernatants were collected and HIV-1 reverse transcriptase (RT) activity was assessed as a measure of viral replication (**Supplementary Figure S11**). The remaining cells were fixed and HIV-1 p24 staining was used as a qualitative measurement of viral infection (**Figure 3**). Treatment with 12.5 or 25 μg/mL of BSNRs-RPV showed a notable reduction in HIV-induced multinucleation of macrophages at days 1, 3, 5, and 8 compared to positive control (HIV infected macrophages). These results paralleled RT activity measurements while demonstrating comparable antiretroviral responses for NRPV. The antiretroviral activity of BSNRs-RPV (98% HIV-1 inhibition) was similar to that of NRPV (98% viral inhibition) after 8 days of infection without any apparent difference of the BSNRs-RPV concentrations.

### Biodistribution and cellular localization of particles by SPECT, LA-ICP-MS and TEM imaging

Our laboratory has been developing a theranostic, nanoparticle-based imaging platform that can be used to predict the long-term biodistribution of these medications by use of MRI and SPECT imaging early in the treatment regimen.^24^, ^33^, ^35^ We hypothesized that a rod-shaped nanoparticle (termed “nanorods”) approximately the same size as our long-acting RPV nanocrystals, created from safe, (bismuth and sulfur) non-toxic “green elements”^36^ could serve as an accurate predictor of RPV long-term biodistribution by intrinsically doping the BNSRs with ^177^Lu, a gamma-emitting radioisotope. If successful, a theranostic imaging platform would allow the creation of a data library to predict drug biodistribution in patients with greater precision than traditional PK and biodistribution analysis without the need for invasive and experiment ending isolation of tissue samples.

**Figure 4:**
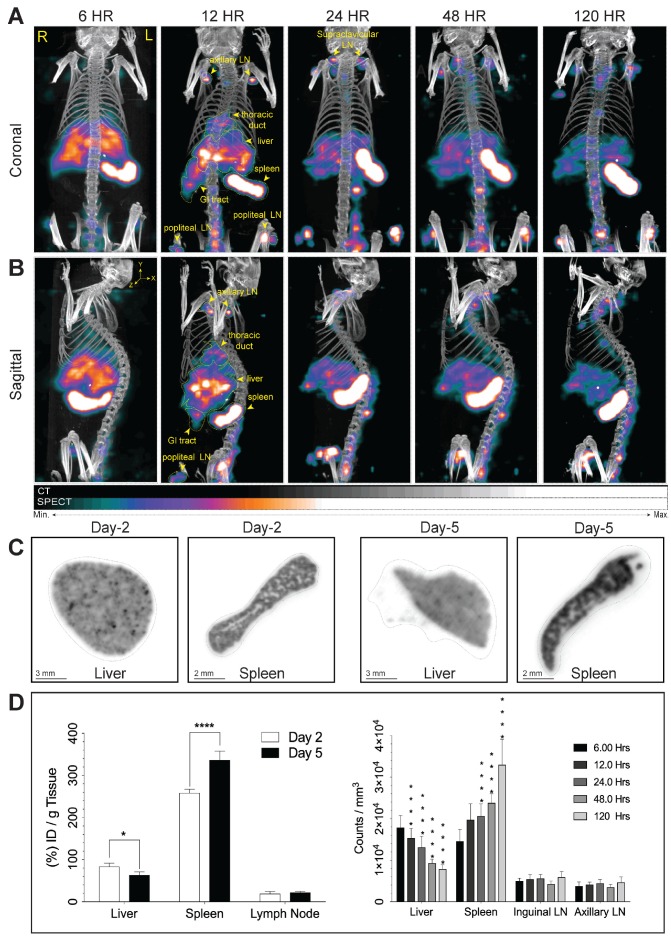
** Real time biodistribution of multimodal particles in mouse tissues.** SPECT imaging was used to assess distribution of intrinsically labeled ^177^LuBSNRs. ^177^LuBSNRs particles (^177^Lu = ~1000 μCi, particle size of ~ 200 nm based on DLS) were injected intravenously into Balb/c mice. Whole body coronal SPECT/CT images were collected at 6, 12, 24, 48 and 120 hours after injection (A). Corresponding sagittal images are also shown (B). ^177^Lu radioactivity intensity is reflected by the colors shown. High signal intensity was detected in the lymph nodes (LN), liver, spleen and gastrointestinal (GI) tract tissues. Images were adjusted for an appropriate fitting with the tracer distribution. The ex-vivo autoradiography studies (C) show homogeneous accumulation of ^177^LuBSNRs in the liver tissue that decreases over time (48 hours > 120 hours). Breakdown of particles in liver could lead to free ^177^Lu absorption in bone, as seen near the lower spinal cord at later times. Accumulation of ^177^LuBSNRs in spleen showed a heterogeneous distribution pattern and increased particle concentrations over time (120 hours > 48 hours). Quantitative studies were done by measuring radioactivity by gamma scintillation spectrometry analysis (left) and SPECT 3D images were used to drawn ROIs (right) to obtain radioactivity counts per volume of tissue (D). Significant differences were determined using two-way ANOVA between groups followed by a Bonferroni's post-hoc test to correct for multiple comparisons. *p < 0.05, ***p < 0.001; ****p < 0.0001.

We assessed the effectiveness of ^177^LuBSNRs particles as multimodal contrast agents for non-invasive real time imaging of biodistribution to the lymphoid organs in Balb/c mice. Biodistribution of ^177^LuBSNRs was investigated by SEPCT/CT at 6, 12, 24, 48, and 120 hours after IV injection, as shown in **Figure 4.** Three-dimensional image reconstruction and autoradiographic counts were performed in excised livers and spleens at days 2 and 5 (48 and 120 hours, respectively). Quantitative studies were performed by measuring radioactivity by gamma scintillation spectrometry and by SPECT three-dimensional images to draw regions of interest (ROIs) and obtain counts per unit volume of tissue. The three-dimensional rendered SEPCT/CT images of the thoracic duct (cisterna chyli), liver, spleen, gut and lymph nodes are illustrated in **Figure 4A** and** B** (coronal and sagittal views, respectively), and illustrate the transition of ^177^LuBSNRs from the liver to lymph nodes over the 120 hour period. In the early time points high deposition of ^177^LuBSNRs in liver and spleen was observed. Radioactive signals in liver decreased steadily over time, with less than half of the radioactivity remaining by 120 hours. A strong signal was observed in spleen within 6 hours which increased over time. After 12 hours, ^177^LuBSNRs were readily observed in the gut and lymph nodes, in addition to liver and spleen**. (Figure 4A-B and supplementary video files SV1-SV5;6 to 120 hours time points).** At 24 hours post injection, ^177^LuBSNRs were deposited largely in spleen and were diminished in the liver. Importantly, 120 hours after particle administration, the particles were highly retained in the spleen while minimal levels were found in the liver **(autoradiography results Figure 4C).** Particle levels in the lymph nodes remained constant from 12 to 120 hours. *Ex vivo* gamma scintillation examination was used to quantify radioactivity in liver, spleen and lymph nodes. Values of radioactivity in spleen were 258 ± 9 and 337 ± 21 %ID/g at 48 and 120 hours, respectively. In contrast, liver radioactivity was 84 ± 8 and 63 ± 8 %ID/g at 48 and 120 hours, respectively **(Figure 4D).** Radioactivity in axillary and inguinal lymph nodes remained constant between the two time points **(Figure 4D left panel).** This data was confirmed by 3D ROIs from SPECT images **(Figure 4D right panel).** Even after 120 hours, no SPECT signal was found in the bladder, demonstrating the stability of ^177^LuBSNRs *in vivo*. The distribution of the ^177^LuBSNRs in the liver and spleen at 48 and 120 hours was heterogeneous as illustrated by *ex vivo* autoradiography **(Figure 4C).** In the spleen, high particle numbers accumulated in the red pulp with limited accumulation in the white pulp.

The utility of BSNRs particles as *in vivo* X‐ray CT contrast agents was also demonstrated using the newer technique of small animal radiation research platform (SARRP) (Xstrahl, Inc.) as illustrated and cited in **supplementary Figure S14.**^37^

**Figure 5:**
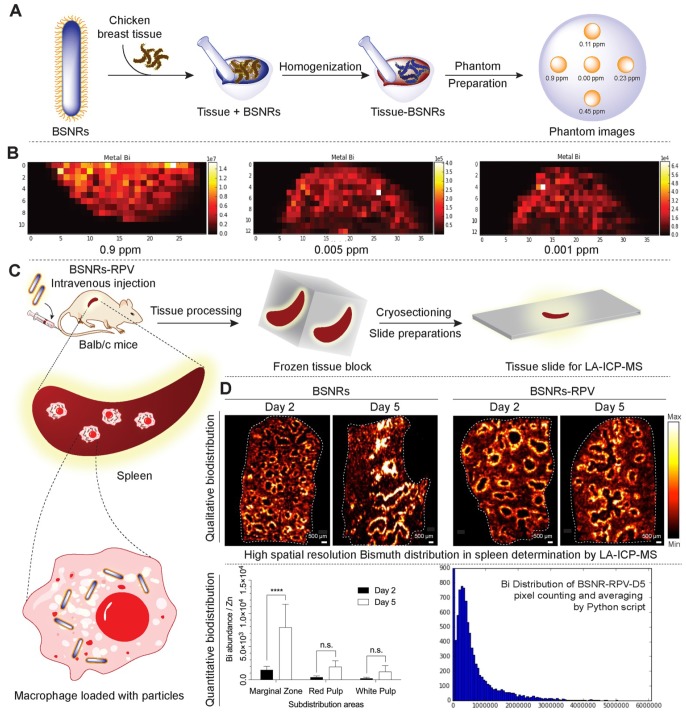
** Tissue particle localization by LA-ICP-MS.** Schematic representation of preparation of in vitro phantoms using chicken breast homogenates mixed with BSNRs particles and creation of the calibration curve from matched matrix (A). Determination of spatial resolution, sensitivity and lower limit of detection (LOD) of BSNRs particles in breast homogenates tissues (B). Lower limit of bismuth detection analyzed by LA-ICP-MS by preparation of “phantom tissue” with particle mixture by using chicken breast tissues homogenate. Serial dilutions of particles plus tissues homogenate mixture were used to create a calibration curves from LA-ICP-MS imaging spots. Bismuth was detectable in tissue mimics at all calibration points. Fifty micrometer resolution is needed to properly observe subcellular distribution. Distribution of particles in spleen slices was determined on days 2 and 5 after injection of BSNRs and BSNRs-RPV particles in mice (C). The distribution of the particles in the spleen was monitored with high spatial resolution and sensitivity using LA-ICP-MS imaging. Bismuth signal in spleen sub regions after injection of particles (D) showed a heterogeneous distribution pattern. Higher distribution in the macrophage-rich marginal zone compared to white pulp and red pulp was observed (D). The uneven particle distribution pattern observed across the structural architecture of the spleen and confirmed by quantitative measurements by LA-ICP-MS can be explained by higher macrophage populations in marginal zone that are specialized for uptake and storage of particles. A two-way ANOVA between groups followed by a Bonferroni's post-hoc test to correct for multiple comparisons was performed to determine statistical differences. ****p < 0.0001; n.s.= not significant. (Associated controls and additional experimental results analysis are presented in **supplementary Figure S15-16**)

**Figure 6:**
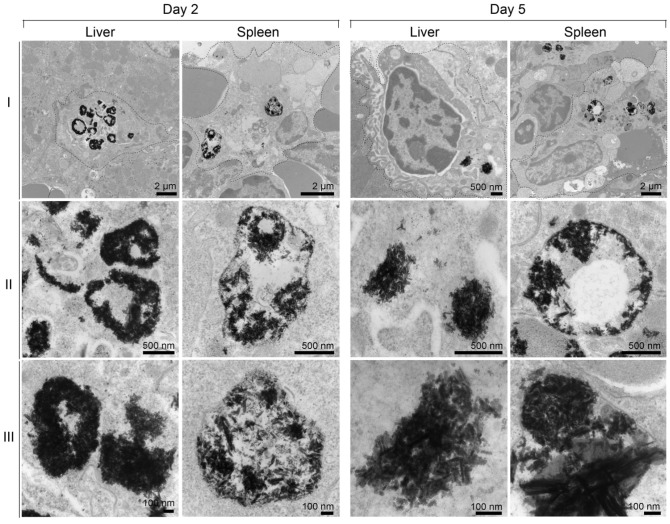
** Subcellular particle localization.** TEM images were obtained for liver and spleen 2 and 5 days after injection of BSNRs particles (80 mg /kg bismuth). Panels (ii) and (iii) are higher-powered images from black color particles deposited regions by from panel (i). Presence of BSNRs particles (black dots) can be visualized in macrophages in both liver and spleen sections

Cellular distribution of the particles in the spleen was investigated by laser ablation inductively coupled plasma mass spectrometry (LA-ICP-MS) imaging. Schematic representation of preparation of *in vitro* phantoms using chicken breast homogenates mixed with particles and creation of the calibration curve from matched matrix is shown in** Figure 5A.** The BSNR particles demonstrated very high spatial resolution, sensitivity and low limits of detection (LOD) close to 0.001 ppm in chicken breast tissue homogenates **(Figure 5B and supplementary Figure S15-16).** Spleen tissues prepared using this approach met the requirements of LA-ICP-MS analysis.^38^ The BSNRs particles *ex-vivo* cellular distribution in spleen using LA-ICP-MS imaging of the bismuth signal showed a heterogeneous distribution pattern of the particles **(Figure 5C, D)**. The results indicated that BSNRs and BSNRs-RPV particles deposited in the spleen to a higher extent on day 5 compared to day 2. Furthermore, distribution of particle patterns in the marginal zones of the spleen was much higher compared to distribution in white and red pulp. Quantitative analysis of the images demonstrated that the primary site of accumulation for particles is in the marginal zones where the maximum number of macrophages are located **(Figure 5D, lower left panel)**. Particles also distributed to the red and white pulp, but to a lesser degree. BSNRs and BSNRs-RPV were uniformly distributed in matrix-matched spleen samples without aggregation **(Figure 5D, lower right panel).** We hypothesized that the particles would be taken up and sequestered by macrophages in tissues leading to particle depot formation that would allow slow release of therapeutic amounts of particles over time. To test our hypothesis, we injected mice intravenously with BSNRs (G6) and prepared liver and spleen sections for TEM imaging at days 2 and 5 (**Figure 6)**. Macrophages containing BSNRs could readily be found with a black aggregate in liver and spleen at days 2 and 5 **(Figure 6, Row I)**. At higher magnifications, macrophages show large amounts of intracellular BSNRs particles **(Figure 6, Row II)**.

Increasing magnifications to 5x that of row **II** allowed validation of particle deposition in macrophages with clear visualization of individual BSNRs, and clusters of BSNRs within cells **(Figure 6, Row III)**. The fact that BSNRs in cells appear to cluster, suggests the particles are taken up by endosomal vesicles in a similar manner to that observed with other nanoparticle platforms.^24^, ^35^

### Tissue drug and metal analysis profile

**Figure 7:**
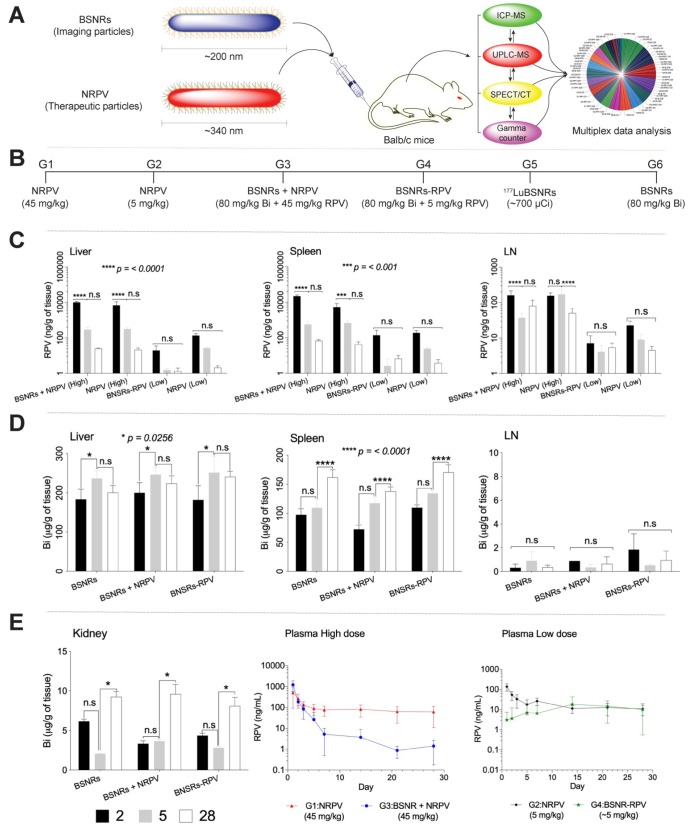
** BD and PK.** A schematic representation of the nanoformulated therapeutic particles (NRPV) and multimodal imaging particles (BSNRs) is shown in (A). The type of treatment and particle size administered to mice, and analyses techniques used are illustrated. Treatment scheme for intravenous injection of high dose NRPV (G1) (45 mg/kg RPV), low dose NRPV (G2) (5 mg/kg RPV), BSNRs (80 mg/kg based on bismuth) plus NRPV (45 mg/kg RPV) (G3), BSNRs-RPV (80 mg/kg based on bismuth plus ~5 mg/kg RPV) (G4), ^177^LuBSNRs (37 MBq = ~1000 µCi = ~ 80 mg/kg based on bismuth) (G5) and plain BSNRs (80 mg/kg based on bismuth) in male Balb/cJ mice is shown in (B). RPV levels in liver, spleen, and lymph nodes 2, 5, and 28 days after treatment of mice with NRPV high and low dose, BSNRs plus NRPV and BSNRs- RPV were determined by UPLC-MS/MS (C). Bismuth contents in liver, spleen, lymph nodes and kidneys from mice treated with BSNRs and BSNRs plus NRPV and BSNRs-RPV were determined by ICP-MS from days 2, 5, and 28 (D-E left side). Plasma RPV concentrations of mice treated with NRPV high and low dose, BSNRs plus NRPV and BSNRs- RPV were determined on days 1 through 28 by UPLC-MS/MS. Statistical differences were determined using two-way ANOVA between groups. Multiple comparison correction was done using a Bonferroni's post-hoc analysis. *p < 0.05 (p = 0.0256); ***p < 0.001; ****p < 0.0001; n.s.= not significant.

To determine whether ^177^LuBSNRs particles could provide an accurate estimate of the long-term biodistribution of long-acting antiretroviral medications, we compared the distribution of BSNR particles to that of NRPV as a model long-acting medication. As shown in **Figure 7A**, male Balb/c mice received a single intravenous injection of either therapeutic particles (NRPV) or imaging particles (BSNRs) at various doses and in combination. The biodistribution of the different particles was assessed at various times post-injection using *in vivo* SPECT imaging and *ex vivo* ICP-MS and UPLC-MS/MS analyses for metal and drug content in tissues and plasma. Additionally, *in vivo* SPECT and LA-ICP-MS imaging results were complemented by measuring radioactivity in excised tissue samples of treated mice by gamma scintillation analysis and autoradiography. The various treatment groups are shown in **Figure 7B**. **Table 3** displays the analytical technique used to measure each parameter for every group and at what time point each parameter was measured. In brief, the UPLC-MS/MS results for RPV content in tissues for groups treated with NRPV or BSNRs-RPV are shown in **Figure 7C**.

The levels of RPV in liver were similar for animals treated with a high dose (45 mg/kg) of NRPV alone (G1) or in combination with BSNRs (G3). After two days, the RPV content in livers from G1 animals was approximately 6883.2 ± 4074.54 ng/g. By day 5 the liver RPV concentration decreased to 317.2 ± 61.15 ng/g. By the end of the study, 28 days after injection, 21.3 ± 5.7 ng/g RPV remained in the livers of G1 animals. RPV content in the livers of G3 (BSNR plus NRPV) animals showed a similar pattern with RPV content of 10,033.6 ± 1344.96 ng/g, 291.7 ± 93.28 ng/g, and 25.0 ±1.7 ng/g for days 2, 5, and 28, respectively. The pattern of RPV concentration over time in the livers of animals treated with a lower dose (5 mg/kg) of NRPV (G2) or BSNRs-RPV (G4) was also similar. G2 livers contained 139.9 ± 37.9, 26.8 ± 2.6, and 2.1 ± 0.55 ng/g RPV on days 2, 5, and 28, respectively. RPV content in G4 livers was lower than that of G2 livers at 19.7 ± 15.6, 1.6 ± 0.35, and 1.3 ± 0.67 ng/g RPV on days 2, 5, and 28, respectively, but the trend over time was similar.

RPV concentrations in the spleens of G1 (NRPV 45 mg/kg) animals were lower than those in liver on day 2 but higher on days 5 and 28 **(Figure 7C)**. Concentrations were 5250.4 ± 3063 ng/g RPV on day 2, 683.6 ± 221 ng/g RPV on day 5 and 42.5 ± 16.86 ng/g RPV on day 28. For G3 (BSNR plus NRPV 45mg/kg) animals, RPV concentrations in spleen were twice that of the liver RPV concentrations on all days with values of 22,020.1 ± 3971 ng/g RPV on day 2, 569.2 ± 215.4 ng/g on day 5, and 67.7 ± 11.04 ng/g on day 28. RPV content in the spleens of G2 and G4 animals, which received a lower dose of NRPV or BSNRs-RPV, were similar to concentrations in autologous livers. For G2 spleens, RPV concentration was 189.2±64.5 ng/g on day 2, 24.4±3.8 ng/g on day 5, and 3.8±2.5 ng/g by day 28. Splenic RPV concentrations for G4 animals closely matched that observed for G2 animals with values of 138.8±121 ng/g, 2.6±3.6 ng/g, and 6.71± 3.3 ng/g RPV on days 2, 5, and 28, respectively.

Concentration of RPV in the lymph nodes was less than that of liver and spleen, but followed a similar pattern over time as in those tissues **(Figure 7C)**. For G1 animals, RPV concentrations in the lymph nodes were 156.6 ± 31.6 ng/g RPV on day 2, 171.4 ± 71.8 ng/g on day 5, and 50.9 ± 16.8 ng/g on day 28. Similar values were obtained from lymph nodes excised from G3 animals with RPV concentrations of 160.9 ± 52.5 ng/g, 37.3 ± 12.4 ng/g, and 80.4 ± 37 ng/g on days 2, 5, and 28, respectively. RPV concentrations in lymph nodes of animals treated with the low dose of NRPV (G2) decreased over time with values of 22.8 ± 7.3 ng/g on day 2, 4.1 ± 3.8 ng/g on day 5, and 5.4 ± 1.2 ng/g on day 28. However, the RPV content of lymph nodes from animals treated with RPV loaded BSNRs (BSNRs-RPV) was lower than that in G2 animals on day 2 (7.1 ± 4.4 ng/g) and remained constant thereafter through day 28 (5.4 ± 1.8 ng/g).

Bismuth concentrations in tissues over time from animals in G3 (BSNRs plus NRPV), G4 (BSNRs-RPV) and G6 (BSNRs only) were determined by ICP-MS analysis. The results for liver, spleen, lymph nodes, and kidneys are shown in **Figure 7D and E**. For G6 animals, bismuth concentration in the liver increased significantly (P<0.05) from day 2 to day 5, from 183.3 ± 26.19 μg/g to 237.2 ± 19.41 μg/g and decreased slightly to 200.7 ± 17.88 μg/g by day 28. Bismuth content in the livers of G3 animals showed a similar pattern with a significant increase in bismuth concentration from day 2 to day 5 (200.0 ± 26μg/g bismuth to 246.7 ± 18.3 μg/g bismuth) followed by a non-significant drop to 223.9 ± 19.3 μg/g on day 28. Again, a similar pattern was observed in G4 livers in terms of bismuth concentration. On day 2, liver concentration was 181.9 ± 36.4 μg/g bismuth followed by a significant accumulation by day 5 (251.6 ± 42 μg/g) and a non-significant decrease by day 28 (241.1 ± 13.7 μg/g).

The concentration of bismuth in spleens showed a pattern of non-significant accumulation between days 2 and 5, followed by a significant increase in concentration between days 5 and 28 for each of the three groups of animals that received BSNRs (G3, G4, and G6). The concentration of bismuth in G3 spleens increased from 72.4 ± 7.0 μg/g to 117.4 ± 11.2 μg/g from day 2 to day 5 and then further increased to 137.8 ± 7.6μg/g by day 28. For G4 spleens a similar pattern was observed, with a non-significant increase from 109.7 ± 4.9 μg/g to 134.4 ± 4.8 μg/g bismuth from day 2 to day 5 followed by a larger, significant increase to 170.6 ± 13.1 μg/g by day 28. Bismuth concentration in G6 spleens followed the same pattern with measured values of 97.5 ± 10.3 μg/g, 109.5 ± 13.7 μg/g, and 162 ± 13.1 μg/g bismuth on days 2, 5, and 28, respectively.

Concentrations of bismuth in lymph nodes of G3, G4, and G6 animals were lower than that in the spleen and liver. However, lymph node concentrations of bismuth did not change significantly over the course of 28 days for any of the three groups. For G3, bismuth concentration in the lymph nodes on day 2 was 1.84 ± 0.001 μg/g with concentrations on day 5 and day 28 of 0.89 ± 0.2 μg/g and 0.35 ± 0.5 μg/g, respectively. The bismuth concentration in the lymph nodes of G4 animals was similar to that in G3 animals with values of 0.88 ± 1.3 μg/g, 0.34 ± 0.07 μg/g, and 0.64 ± 0.7 μg/g on days 2, 5, and 28, respectively. G6 animals received only BSNRs and the bismuth concentration in lymph nodes was 0.31 ± 0.3 μg/g on day 2, 0.89 ± 0.7 μg/g on day 5, and 0.36 ± 0.1 μg/g, on day 28. Overall the concentration of bismuth in the lymph nodes of all animals remained relatively constant over the 4 weeks post-treatment. Bismuth concentration was also measured in the kidneys of G3, G4, and G6 animals by ICP-MS **(Figure 7E, left panel)**. Again, all groups followed a similar pattern of Bismuth concentration over time. Between days 2 and 5, the concentration of bismuth changed non-significantly from 3.34 ± 0.35 μg/g to 3.65 ± 0.21 μg/g, 4.35 ± 0.3 μg/g to 2.82 ± 0.15 μg/g, and 6.17 ± 0.2 μg/g to 9.24 ± 0.05 μg/g for G3, G4, and G6 animals respectively. From day 5 to day 28 all groups saw a significant accumulation of bismuth in the kidneys with concentrations of 9.60 ± 1.2 μg/g, 8.09 ± 1.07 μg/g, and 9.24 ± 0.70 μg/g for G3, G4, and G6, respectively.

RPV concentrations were assessed over time for groups that received NRPV (G1, G2, G3) or BSNRs-RPV (G4) **(Figure 7E, center and right panels)**. On day 1 RPV concentration in G1 plasma was 520.0 ng/mL and declined rapidly to 76.7 ng/mL by day 7, then remained fairly constant until day 28 at 61.9 ng/mL **(Figure 7E, center panel)**. Plasma concentration of RPV in G3 animals followed a similar pattern over time. On day 1, RPV concentration in G3 plasma was 1229.1 ng/mL and then decreased to 5.25 ng/ml at day 7. From day 7 to day 28 there was a very slow decrease in plasma concentration to 1.4 ng/mL.

For G2 animals, RPV concentration was 137.6 ng/mL at day 1 and declined to 17.8 ng/mL by day 5 **(Figure 7E, right panel)**. From day 5 until day 28, plasma RPV concentration slowly declined to 10.8 ng/mL. For G4 animals treated with BSNRs-RPV, plasma RPV concentration on day 1 was relatively low, 3.1 ng/mL, but increased to 18.4 ng/mL by day 14. From day 14 to day 28 the plasma RPV concentration remained constant and on day 28 plasma RPV was 10.2 ng/mL. These results suggest a slower release of RPV into the plasma from BSNRs-RPV than from NRPV alone, potentially prolonging its effects in the body. *In vivo* treatment with BSNRs, BSNRs-RPV, NRPV, and combinations of BSNRs plus NRPV produced no observed toxicity in liver, kidney, or pancreas as determined by serum chemistry and histological analysis results **(Supplementary figures 20 and 21).**

### Metadata analysis

**Figure 8:**
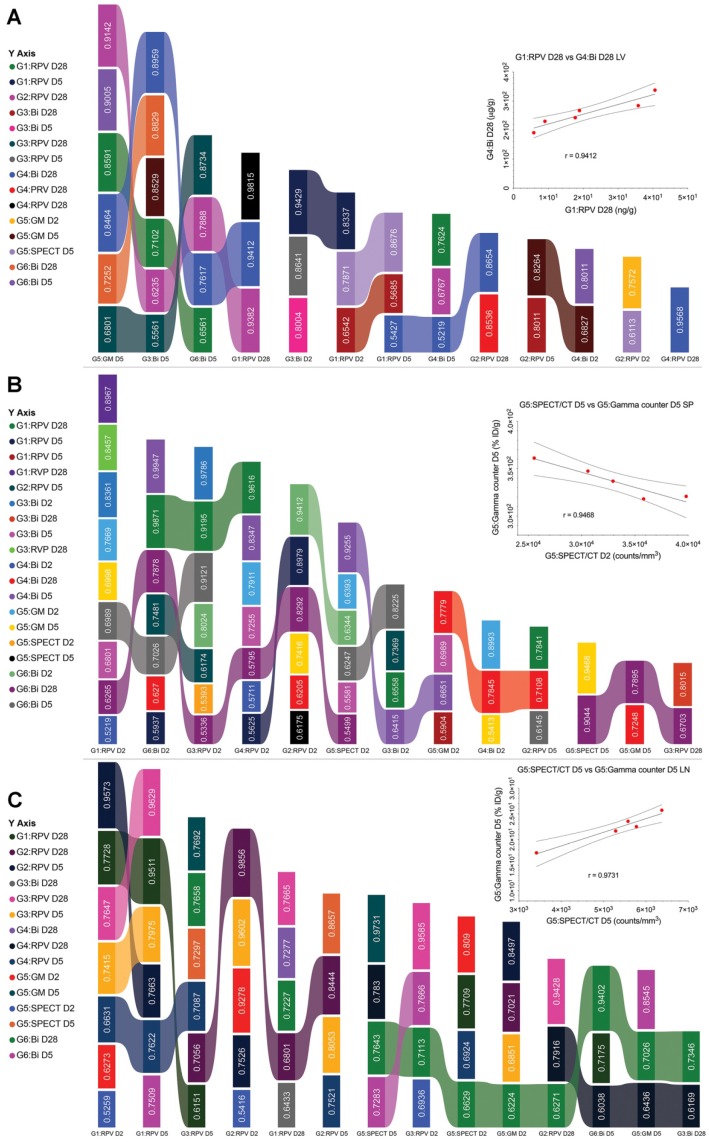
** Multiplex statistical data analyses.** Pearson's correlation coefficients metadata were generated from SPECT, UPLC-MS/MS (RPV), ICP-MS (Bismuth) and gamma scintillation analysis of liver (A), spleen (B), lymph node tissues (C). Key components calculated across groups with fitting Pearson's correlation coefficients using a p-value ≤ 0.05 are shown as multiplane ribbon plots (**Supplementary Figure S17-19**)**.**

LA ARVs could overcome limitations in HIV/AIDS therapeutic regimens that limit optimal clinical outcomes.^14^ The ability to sustain plasma and tissue drug levels beyond the plasma 90% inhibitory concentration (IC_90_) for months would reduce pill burdens, increase regimen adherence and reduce adverse events that plague current medications. This would also lift the psychological stigma for strict daily regimens reminding patients of disease and impacting mental health.^16^, ^39^, ^40^ To simplify measurements of LA ARV efficacy by pharmacokinetic (PK) and biodistribution (BD) tests and optimize dosing, theranostics was introduced.^16^ The traditional PK and biodistribution assessments which require end-point animal sacrifice and organ removal could be exchanged by bioimaging-based measurements.^24^ To determine whether rod-shaped particles were reflective of the PK and biodistribution of LA NRPV, biodegradable BSNRs formulations were created. These newly synthesized particles were then deployed as a predictor of RPV long-term biodistribution. ^177^LuBSNRs were used as a theranostic imaging platform to compare six treatment regimens. After group data was acquired across time, predictive values were determined for particle drug biodistribution. Pearson's correlation coefficients were calculated for group comparisons of RPV concentrations, bismuth concentrations, SPECT ROI **(Supplementary figure S13),** and gamma scintillation counts from excised organs. Means of absolute “r” values with p ≤ 0.05 were used to create the “multiple ribbon bar graph” (**Figure 8**).

From imaging analyses, biodistribution and PK studies data for various parameters were measured in plasma and tissues for 6 treatment groups with the analysis parameter depending on the treatment **(supplementary figure S12)** as shown in **Table 3**. These parameters included RPV concentration (UPLC-MS/MS), bismuth concentration (ICP-MS), SPECT signal, and gamma counts for excised organs. To assess the predictive value of the BSNRs to estimate long-term biodistribution of LA ARVs such as NRPV, we used Pearson's correlation analysis for 2 parameter comparison on averaged data collected from different groups on the same day. Correlation “r” values were generated for each comparison and statistically significant correlations with r > 0.5 were plotted. For same day comparisons in the liver, the highest degree of correlation was between RPV and bismuth on day 2 (r = 0.9796). Additionally, G1 RPV concentration in the liver correlated significantly with bismuth concentration in G4 animals treated with BSNRs-RPV (r = 0.9412). Interestingly, the lower dose of NRPV (G2) correlated extremely well with our BSNRs-RPV (G4) in terms of RPV concentration even to 28 day with r = 0.8536. These data demonstrated that RPV loaded BSNRs reflected the biodistribution of NRPV. Gamma scintillation analysis of G5 livers excised 5 days post-treatment correlated with bismuth concentration (r = 0.9005). Furthermore, gamma counts of excised day 5 livers correlated with RPV concentration on the same day (r = 0.8264). Most importantly, we observed that SPECT signal and RPV concentration on day 5 were strongly correlated (r = 0.8676). In spleen, RPV concentration (G2) correlated with bismuth concentration, gamma scintillation counts, and SPECT signal (r = 0.9412, 0.8361, and 0.5593, respectively). SPECT signal (G5) in spleen also significantly correlated with bismuth concentration on day 2 (r = 0.8993) for animals treated with BSNRs-RPV (G4). In lymph nodes, SPECT signal (G5) correlated with RPV concentration on day 2 from multiple groups including G1, G2, and G3 with r = 0.5259, 0.5416, and 0.6936, respectively **(Supplementary figure 19 graphs A, C and E)**. We observed an even stronger correlation between SPECT signal (G5) and RPV concentration (G2) on day 5 (r = 0.9278) **(Supplementary figure 19 graph D)**. Day 5 lymph node SPECT signal also correlated with bismuth concentration (r = 0.7283), providing evidence that the SPECT signal could be used to predict RPV concentration *in vivo*. Gamma scintillation counting was also closely associated with bismuth and SPECT signal (r = 0.8545 and 0.9731 respectively).

The data, *in toto*, demonstrates that SPECT signal reflects RPV drug concentrations. We also looked at the associations between various different data combinations (e.g., RPV vs Bismuth) acquired at different times to determine the predictive value of the BSNRs platform. In the lymph nodes, we observed that SPECT signal at 2 days post-treatment was predictive for RPV concentration on day 28, for both high dose NRPV group (G1) and RPV-BSNRs (G4) with strong correlation values (r = 0.7709 and 0.7830, respectively). SPECT signal from day 2 was also predictive for day 28 bismuth concentration in the lymph node (r = 0.7643). In the spleen, SPECT signal from days 2 and 5 could be predict bismuth concentration on day 28 (r = 0.9255 and 0.9044, respectively), while day 2 bismuth concentrations correlated with day 28 RPV concentration (r = 0.987). In liver, gamma scintillation counts from day 5 provided the best correlative values in terms of RPV and bismuth content on day 28. Day 5 gamma scintillation counts correlated with RPV concentrations from G1, G2, and G3 (r = 0.8591, 0.9142, and 0.6801, respectively). Group correlations for spleen parameters were also determined and are illustrated in **supplementary figures S17-19**. Overall the correlative data obtained from the tissues provides evidence that bioimaging by SPECT accurately predicts LA RPV drug levels in lymphoid tissues.

## Discussion

While drug-encased theranostic nanoparticles are developed for cancer and inflammatory disease 41-44 little has yet to be achieved for HIV diagnostics and treatments. Thus, an immediate need warrants the creation of theranostic ARV particles optimized for size, shape, and structure to ensure proper distribution to CD4^+^T cells and macrophages. All will ensure improved PK as the drug's apparent half-life is extended. To achieve these goals and to simplify assessments of LA ART for PK, biodistribution and dosing theranostics was employed. Traditional PK requires end-point animal sacrifice and organ removal. These we posit can be exchanged by bioimaging tests. To determine whether rod-shaped particles are reflective of LA RPV size and shape and as such could predict drug biodistribution biodegradable bismuth and sulfur formulations were created. We assessed, in real time, the biodistribution of drug loaded multimodal radiolabeled particles (^177^LuBSNRs) and used them to predict LA RPV pharmacological properties. The BSNRs-RPV particles were used successfully to determine ARV effectiveness through measures of drug distribution into HIV tissue and cell reservoirs by noninvasive measurements.

Over the past two decades our laboratories investigated the role of particle size and shape for their distribution.^24^, ^35^, ^45^-^47^ The results showed that rod-shaped nanoparticles were efficient and rapidly taken up in macrophages.^33^, ^48^-^50^ The combinatorial effect of particle size and shape reflects Fc receptor mediated macrophage engulfment and internalization. Indeed, nanoformulations with a rod shape were known to be taken up by macrophages at faster rates than spherical particles.^48^ Indeed, prior research into the shape of mesoporous silica nanoparticles demonstrated that those particles with larger aspect ratios were internalized faster.^51^ These results supported other previously published reports.^52^, ^53^ Based on need coupled with prior studies demonstrating a “rod-like” particle shape is an efficient entry into macrophages as the principal depot, we developed theranostic ARV formulations to reflect the shapes of native LA drugs.^54^-^57^ Moreover, coating the surface of nanoparticles with polyethylene glycol (PEG), termed “PEGylation”, was shown to improve the efficiency of drug and gene delivery.^58^, ^59^ Firstly, PEGylation reduces aggregation of particles by diminishing the association with nontargeted serum and tissue proteins and resulting `stealth' behavior.^60^ The PEG polymers reduces charge-based contact typical of proteins and small-molecule interactions.^61^ Second, PEGylation increases the circulation time of nanoparticles by preventing non-specific uptake by the liver and spleen.^62^ Third, PEGylated nanoparticles have increased solubility in buffer and serum due to the hydrophilic ethylene glycol repeats. Fourth, PEGylation reduces systemic and cellular toxicities of nanoparticles.^63^ In all, these properties underlie the properties of the PEG polymer formed by repeating ethylene glycol monomers. PEG polymers of varying lengths exist and some reports number of ethylene glycol monomers in a chain of up to 40. PEG molecular weights are up to 20,000 Da and the thickness of any grafted PEG correlates with its molecular weight and density as present on the nanoparticle surface.^64^ In our studies, using PEG2000 was enough to consistently generate nanorods of the same size as our nanoformulated RPV. PEGylation also results in a tighter size distribution of produced particles by decreasing aggregation. In terms of increasing therapeutic efficacy, PEG coatings increase the circulation half-life of nanoparticles but also improve the penetration of particles into “biological barriers”, including reducing interactions with tissue extracellular matrix, cellular barriers, and biological fluids such as mucus, especially when delivering poorly water-soluble drugs.^65^, ^66^ Overall, it is a combination of the density of PEG polymers and the number of polyethylene glycol repeats/polymer that affect therapeutic efficacy and particle size.

LA ART is a means to overcome limitations in HIV therapeutic regimens that often preclude best clinical outcomes.^14^-^16^, ^67^ The ability to sustain plasma and tissue drug levels above effective plasma concentrations for months would have a real impact on the care and prevention of viral infection. Moreover, it would reduce pill burden, increase regimen adherence, and limit adverse events that plague current drug regimens. This would also lift the stigma for strict daily regimens that remind patients of their disease.^16^, ^39^, ^40^

As a result, we hypothesized that BSNRs with a clear-cut, rod shape morphology that matches the shape and size of our long acting nanoformulated therapeutic drug nanoparticles provide a platform to accurately and efficiently predict long-term therapeutic drug biodistribution and that these tests could be performed by SPECT and LA-ICP-MS. To achieve this goal, theranostic particles were made of identical size and morphology (“rod-shaped”) as LA ARVs that are used currently in phase III treatment paradigms.^68^ This was achieved in step-wise fashion. *First*, we created lipid coated ^177^LuBSNRs as a multimodal imaging particle for non-invasive and real-time imaging. *Second*, LA RPV (NRPV) was deployed to model drug biodistribution. Imaging enabled particle tracking in real time and specifically tracked the distribution into “putative” tissue reservoirs of infectious virus. *Third*, the particles allowed direct and real-time visualization for the abilities of drug to distribute to lymphoid organs. *Fourth*, state of the art characterizations that included surface modification of imaging particles with a thin lipid layer provided high cell uptake and aqueous solubility, biocompatibility and stability. *Fifth*, cell-based experiments demonstrated rapid particle uptake, retention, and antiretroviral efficacy in target macrophages. Particle uptake remained constant from 4 to 8 hours possiblely due to cells reaching their capacity or exocytosis at later times. Macrophages rapidly internalized rod-shaped nanoparticles compared to spherical particles.^48^, ^69^, ^70^ Because the uptake mechanism used by the macrophages was phagocytosis, shape, size, surface properties as well as quantity of particles played a significant part in uptake.^48^, ^50^, ^69^, ^70^ The eight days of antiretroviral efficacy exhibited by BSNR-RPV accords with similar studies that found between 5 and 20 days of protection with NRPV.^71^ Provided that on average more than 1 microgram was retained per million cells, which positively correlated with bismuth intracellular concentrations, it can be surmised that RPV depots were maintained until our assay's terminus. Numerous reports characterize RPV's potency in the sub-nanomolar range. 71, 72 Therefore, the equivalent levels of long-term protection regardless of treatment concentration in our work may be explained in that macrophages treated with low- and high-dose BSNR-RPV maintained drug concentrations above RPV's 50% maximal effective concentration (EC_50_) for at least eight days. *Sixth*, particles' use as a multi-modality agent was confirmed by both SPECT, LA-ICP-MS, ICP-MS and UPLC-MS/MS in mice. Such imaging allowed, for the first time, sub-organ localizations in live animals with a high degree of sensitivity and specificity, exceeding those acquired by conventional magnetic resonance imaging (MRI).

In recent years, the development of bismuth nanorods, nanoparticles, nanodots, and nanoplates has been described for a wide range of biomedical and bioimaging applications.^28^, ^73^, ^74^ These include therapeutics and drug delivery for metabolic, inflammatory, and cancerous diseases and use as biosensors, photothermal and contrast agents.^75^-^78^ While the field of BSNRs theranostics remains in its infancy, the primary challenge in next step applications rests in enhancing radiolabeling efficiency and drug loading. Notably, non-radiolabeled BSNRs have been successfully used as imaging agents as “bismuth” has an ultrahigh X-ray attenuation coefficient (bismuth, 5.74 cm^2^/^g-1^ at 100 keV).^27^, ^79^ High blood and renal concentrations of bismuth ions are considered safe.^80^-^83^ Bismuth is considered one of the most biocompatible metals having been widely employed as an anti-diarrheal (bismuth subsalicylate); an antimicrobial (for eradication *Helicobacter pylori*, *Pseudomonas aeruginosa*, *Burkholderia multivorans* and *B. cenocepacia*); and as an antidote for cadmium poisoning.^84^ The precursor chemicals of BSNRs synthesis are comparatively inexpensive compared to other metals, such as gold and platinum.^85^ Bismuth and sulfur components easily undergo oxidative decomposition to soluble bismuth (III) species under physiological conditions and show considerable lipophilicity and membrane permeability, as such, differ substantively from other metal particles.^86^, ^87^ Our results demonstrate that particle shape influences particle distribution in regional zones of spleen. This indicates that ^177^LuBSNRs readily accumulate in lymphoid organs, including gut, indicating the rapid migration and deposition of particles in these organs where maximum number of HIV-1 are present. This observation matched SPECT results that indicated highly particle uptake and deposition in tissues with large numbers of macrophages and other immune cells. Such multifunctional imaging (^177^LuBSNRs) and therapeutic particles (NRPV) will facilitate rapid screening of antiviral drug tissue distribution.

Each imaging technology that was or can be employed in future research has its own inherent limitations.^88^ Information attained from single imaging modality tracking nanoparticles typically cannot satisfy the requirement to directly visualize infection sites and quantify the amount of drug at those sites. Hence, combination of more than two imaging modalities into a single particle, known as an “all-in-one” nanostructure, allows for spatial temporal assessments of virus replication sites. SPECT generates real-time, high-resolution 3D reconstructions.^89^, ^90^ The SPECT bimodality imaging of lymphoid organs with lipid/calcium/phosphate (LCP) nanoparticles grafted with ^111^In has been reported.^91^ Similarly, recent reports demonstrate that bismuth nanocrystals encapsulated in PLGA particles can be used effectively as a CT contrast agent.^92^ Lipid coating on the BSNRs facilitates such use as it prevents aggregation in physiologic conditions.^93^-^95^ Considering these advantages, SPECT imaging through radiolabeled BSNRs allows direct visualization of tissue sites of viral replication, providing a notable advantage for finding where residual virus may hide from immune surveillance mechanisms.^96^, ^97^, ^98^ Interestingly, BSNRs concentrations were found in spleen at higher levels than in the liver. The extensive metabolism in liver may facilitate more rapid organ clearance for BSNRs compared to spleen.^99^ Finally, the decomposition of BSNRs in liver due to plasma proteins on the surface of particles may also facilitate clearance.^100^, ^101^

Liver metabolism of our BSNRs particles could result in the leaching of particle components that include free ^177^Lu. This concern coupled with signals observed in or near bones plates in later evaluated time points could affect experimental results. 102 Nonetheless, we affirmed the specificity of the SPECT data as popliteal and axillary lymph nodes and bone were independently dissected and subjected to gamma scintillation analysis. These data sets further confirmed the SPECT specifically and both assays were concordant. Thus, while release of ^177^Lu ions may occur its effects on the overall interpretation of particle-drug biodistribution is limited.

Our analyses revealed that BSNR-RPVs could serve as a predictor for RPV drug levels and effectiveness*.* In fact, 5 days after injection, SPECT signal and RPV concentration were correlated strongly, one with the other (r = 0.8676). In spleen, RPV correlated with bismuth concentration, gamma scintillation counts, and SPECT signal (r = 0.9412, 0.8361, and 0.5593, respectively). In lymph nodes, SPECT signal correlated with RPV concentration on day 2 (r = 0.6936). We observed an even stronger correlation between SPECT signal and RPV concentration on day 5 (r = 0.9278). Day 5 lymph node SPECT signal also correlated with bismuth concentration (r = 0.7283), providing evidence that the SPECT signal could be used to predict RPV or bismuth biodistribution in a treated animal*.*

LA-ICP-MS imaging is a powerful tool for providing sub-organ distribution information, enabling greater insight into the biological fate of nanoparticles. The quantitative capability of such imaging assays also facilitates delivery optimization by providing comparative formulation metrics.^103^, ^104^, ^105^ The use of laser ablation allows monitoring of each tissue type with resolutions between 25 to 50 µm demonstrating cell nanoparticle distributions. When properly configured, LA-ICP-MS can image the distributions of nanoparticles with core diameters of 2 nm present at low parts-per-billion concentrations.^106^ Because bismuth has a very low biological background, the BSNRs are an almost ideal nanomaterial for fully exploiting the resolution capabilities of LA-ICP-MS.

In conclusion, we have demonstrated the feasibility of theranostics for studies of LA ARV biodistribution and activity. Using both SPECT and LA-ICP-MS analyses ^177^LuBSNRs were shown to be a viable tool to monitor the distribution and transport or ARVs and in particular RPV to macrophage rich lymphoid tissues and liver. The PK and biodistribution patterns obtained with particle are comparable to therapeutic particles now being employed in treatment paradigms for HIV-1 infection.^24^, ^33^ Moreover, the results were cross validated by statistical models providing a framework for predictive assessments of LA ARVs distribution and activities in an infected human host.

## Experimental section

### Reagents and chemicals

Bismuth neodecanoate (Bi(OCOC(CH_3_)_2_(CH_2_)_5_CH_3_)_3_), thioacetamide (CH_3_CSNH_2_), oleic acid, oleylamine, L-α-phosphatidylcholine (PC) (from egg yolk), 3-(4,5-dimethylthiazol-2-yl)-2,5-diphenyltetrazolium bromide (MTT), were obtained from Sigma Aldrich, St. Louis, MO, USA. Corden Pharma International (Plankstadt, Germany) supplied the 1,20-distearoyl-phosphatidylethanolaminemethyl-polyethyleneglycol conjugate 2000 (DSPE-PEG_2000_). Hangzhou Bingo Chemical Co., Ltd. (Hangzhou, ZJ, China) provided the rilpivirine (RPV). Lutetium (III) chloride, anhydrous, 99.9% (LuCl_3_) was received from Alfa Aesar, Haverhill, MA, USA. ^177^Lutetium chloride (^177^LuCl_3_) in 0.05 M HCl, mass = 3.0 µg was requested and delivered from Oak Ridge National Laboratory, Oak Ridge, TN, USA. Pluronic^®^ F 108 Pastille (P338) was bought from BASF, Florham Park, NJ, USA. Agilent Technologies, (Santa Clara, CA, USA) was the source of the monoclonal mouse HIV-1 p24 (Clone Kal-1) and Dako EnVision+ detection system HRP labeled polymer anti-mouse antibodies used in this paper. 33, 35, 71 Leukapheresis was performed on HIV-1,2 and hepatitis B seronegative donors and human peripheral blood monocytes were obtained and purified by countercurrent centrifugal elutriation. Monocytes were differentiated into macrophages according to our previously published protocols. 24, 35, 33, 71

### Animals

Male Balb/cJ mice (6-7 weeks old) were purchased from Jackson Laboratories (Bar Harbor, Maine, USA). Animals were housed in the University of Nebraska Medical Center (UNMC) Comparative Medicine animal facility according to the Association for Assessment and Accreditation of Laboratory Animal Care guidance. The UNMC Institutional Animal Care and Use Committee approved all protocols related to animal experiments and were certified to have met the requirements and ethical guidelines set forth by the National Institutes of Health in handling laboratory animals for research.

### Preparation of rilpivirine (RPV) freebase and manufacture of nanoformulated RPV (NRPV)

RPV freebase was prepared then nanoformulated as previously described. 33, 71 Nanoformulated RPV (NRPV) was manufactured using an Avestin EmulsiFlex-C3 high-pressure homogenizer. Briefly, Pluronic 338 (P338) and RPV free-base were dispersed in water at concentrations of 0.5% and 1% (w/v), respectively, and mixed (400-600 rpm) overnight at room temperature. Presuspensions were homogenized at 20,000 psi until homogeneous nanocrystals in terms of size (nm) and polydispersity (PDI) were produced. NRPV was purified and concentrated by centrifugation. Specifically, nanoformulations were centrifuged at 5,000 x g for 5 minutes, at which point supernatants were subject to a second spin of 10,000 x g for 5 minutes. Resultant pellets were resuspended in endotoxin free water. A final centrifugation step of 200 x g for 3 minutes removed any aggregated particles. NRPV (size: 341.8 ± 4 nm, PDI: 0.22 ± 0.03, zeta potential: -9.06 ± 0.4 mV) was characterized by dynamic light scattering and assayed for drug content (56.8 mg/ml) by UPLC-UV/Vis using previously described methods. 33, 71 To test drug loading content, NRPV was frozen at -80˚C and lyophilized to yield a dry powder. Samples were weighed, dissolved in methanol, and analyzed by UPLC-UV/Vis. Drug loading was calculated by the following equation. Drug loaded (%) = (Weight of drug in lyophilized formulation/weight of lyophilized formulation) X100.

### Preparation of intrinsically radiolabeled ^177^LuBSNR particles

Intrinsically radiolabeled ^177^LuBSNRs particles, were prepared by a solvothermal technique.^26^, ^28^ A typical reaction scheme is available in the **Supplementary Figure S1 top panel** (imaging particles). Bismuth neodecanoate (290 mg, ~400 µM) and 4 mL of oleic acid (OA) were mixed in a glass vial and stirred for 10 min. Subsequently, ~ 1665 MBq (~45 mCi) of ^177^LuCl_3_ and 4 mL ethanol were mixed in a separate vial and transferred to the bismuth-oleic acid (Bi-OA) mixture. In a 1.5 mL microcentrifuge tube, thioacetamide (30 mg, ~ 400 µM) was quickly added to 800 µL of oleyl amine (OAm) and the mixture was sonicated for 15 minutes to completely dissolve the mixtures. After vigorous stirring for 30 minutes, the colloidal dark black solution was transferred into a 250 mL high safety Teflon-lined reactor that was placed in a stainless-steel autoclave reactor, sealed airtight, and heated at 150°C for 10 hours. The systems were then allowed to naturally cool to room temperature (around 12 hours). After reaching room temperature, the reactor was opened in a controlled, radioisotope work station. Fifty milliliters of ethanol were added followed by vortexing and sonication. The solution was then centrifuged at 976 xg for 30 minutes at 15°C. ^177^LuBSNR particles were collected and purified by repeating the same ethanol washing process three times to remove non-reacted components. Radioactivity of ^177^LuBSNRs was measured by gamma counter (CRC^®^-25R dose calibrator, Capintec, Inc, Florham Park, NJ, USA). Lipid coated ^177^LuBSNR particles were prepared by the thin-film dispersion technique. First, 100 mg L-α-phosphatidylcholine and 50 mg DSPE-PEG_2000_ were dissolved in a chloroform/hexane mixture (5:1 v/v) contained within a round-bottomed flask and evaporated to form a thin film using a rotavap (Heidolph, Hei-VAP Precision; G3B Vertical, USA). The films were dried under vacuum overnight (18 hours) to ensure the complete removal of organic solvents. Second, ^177^LuBSNRs (50 mg particles; ~ 647.5 MBq = ~ 17.5 mCi) were dispersed in cyclohexane (2 mL) followed by bath sonication for 10 minutes to achieve a uniform dispersion of the particles in the solvent. The ^177^LuBSNRs-cyclohexane dispersion was mixed with 2 mL of 2% (v/v) Tween-80 in a 25 mL round-bottom flask and sonicated for 5 minutes to form a turbid metalosome emulsion. Third, cyclohexane was removed from the ^177^LuBSNRs/cyclohexane/Tween-80 emulsion by rotavaporation. Lastly, the ^177^LuBSNRs particle solution was transferred to the lipid film coated round-bottomed flask and dispersed in the lipid film through gentle rotation at 45°C with continuous bath sonication. The end product was collected by centrifugation at 270 xg for 10 minutes. ^177^LuBSNRs in the supernatant was used for particle size and radioactivity analyses. Non-radiolabeled LuBSNRs and drug loaded particles were synthesized by the same technique except that non-radioactive ^175^LuCl_3_ was used instead of ^177^LuCl_3_. BSNRs-RPV particles were prepared with 10 mg of RPV drug in the lipid film. NRPV suspensions were prepared as described previously **(supplementary Figure S1 bottom panel and SM2).**^33^

### Particle characterizations

The morphology, lattice crystal structure, elemental composition, and chemical color mapping of the synthesized BSNRs and ^177^LuBSNRs particles were determined by bright field high-resolution transmission electron microscopy (HR-TEM), selected area electron diffraction (SAED), energy-dispersive x-ray spectroscopy (EDX), and scanning transmission electron microscopy (STEM) with high-angle annular dark-field (HAADF) (FEI Tecnai Osiris S/TEM operated at 200 kV). The TEM samples were prepared in cyclohexane and dried on a copper grid (FCF-400-CU, Electron Microscopy Sciences-EMS, Hatfield, PA, USA) at room temperature. Bright field images were taken with exposure times of 0.5 s. No staining of particles was used, since the heavy metal contrast was adequate for imaging. The additional gain in speed can also be used to collect EDX elemental mappings from a larger field-of-view. The lattice fringes of the obtained samples and the corresponding SAED patterns were examined using HR-TEM at 200 kV. The experimental SAED patterns were analyzed using PCED2 software (Landyne Software, Lincoln, NE, USA).

To determine the shape and size of NRPV particles, the nanosuspension was vortexed and the samples were placed onto a Whatman Nuclepore Track-Etch membrane filter, mounted to the conductive adhesive tape on the scanning electron microscope (SEM) stub, and air-dried overnight. After sputter-coating with a thin layer (~5 nm thick) of chromium using Denton Desk V sputter, the sample was examined with a Hitachi S4700 field-emission SEM. For transmission electron microscopy (TEM), the NRPV particles suspensions were collected using carbon-formvar coated grids and stained with 1% phosphotungstic acid. Samples were examined and imaged using a Hitachi H7500 TEM. The particles were also characterized by Wavelength Dispersive X-ray Fluorescence (WDXRF) analysis using a Rigaku WDXRF (Supermini200) spectrometer with high resolution and lower detection limits for elemental analysis. A 200 W, air cooled, Pd X-ray source was operated at 50 kV and 4 mA to produce the excitation spectrum with faster elemental detection capability. A three-crystal analyzing unit was equipped in the system to support the standard LiF (200), PET, and RX25 crystals for the detection of the elements ranging from ultrafiltration (UF). Particle surface chemistry analysis was performed with X-ray photoelectron spectroscopy (XPS) and measurements were carried out using monochromatic Al Kalpha x-ray with an energy of 1486.6 eV by Thermo Scientific K-alpha+XPS (Thermo fisher scientific, Waltham, MA, USA). Powder X-ray diffraction (XRD) analyses of BSNRs, ^177^LuBSNRs and NRPV were performed in the 2θ range of 2-70° using a PANalytical Empyrean diffractometer (PANalytical Inc.; Westborough, MA, USA) with Cu-Kα radiation (1.5418 Å) at 40 kV, 45 mA settings. A mask of 20 mm and divergence slit of 1/32° were used on the incident beam path. A thin layer of the particle powder sample was placed on a zero-background silicon plate and the sample holder was continuously spun at the rate of 22.5 deg/s during all measurements. The PIXcel3D detector, equipped with a beam monochromator, (PANalytical Inc.; Westborough, MA, USA) was scanned at a rate of 0.053 deg/s. Atomic Force Microscopy (AFM) images were collected with a Nanoscope IV system (Bruker Nano Surfaces, Santa Barbara, CA, USA) in Tapping Mode at ambient conditions. Silicon probes RTESPA-300 with a resonance frequency of ~300 kHz and a spring constant of ~40 N/m were used for imaging at a scanning rate of 2.0 Hz. Image processing and particle size analyses were performed using Femtoscan software (Advanced Technologies Center, Moscow, Russia). Particle sizes of the BSNRs and NRPV was determined by measuring hydrodynamic diameter and particle size distribution in water on a Malvern Nano-Series Zetasizer system (Malvern Instruments Ltd., Malvern, UK). Excitation and emission spectra of BSNRs were determined using UV spectrophotometry (SpectraMax® M3, Molecular Devices, LLC, San Jose, CA, USA). RPV content of BSNRs-RPV and NRPV was determined by reverse-phase ultra-performance liquid chromatography (UPLC) using a Waters Acquity UPLC H-Class System with TUV detector and Empower 3 software (Waters, Milford, MA, USA) as previously described.^33^, ^71^ Bismuth and sulfur quantifications were performed by inductively coupled plasma mass spectrometry (ICP-MS) at the University of Nebraska-Lincoln's Spectroscopy and Biophysics Core Facility, using an Agilent 7500cx ICP-MS (Santa Clara, CA, USA) coupled with a 96-well plate autosampler Model SC/DX4 from Elemental Scientific, Inc. (Omaha, NE, USA), operating in Mix-Gas collision/reaction mode (3.5 mL H2 and 1.5 mL He per minute). Other conditions were: plasma power, 1500 W; carrier gas flow, 1 L/minute; makeup gas flow, 0.15 L/minute; sample depth, 8 mm; plasma gas, 15 L/minute. The concentrations were calculated against an external calibration curve with 50 μg/L of Ga used as internal standard (IS) throughout (^71^Ga isotope). Tissue samples were suspended in 4 times the volume of metal-grade nitric acid, incubated at room temperature for up to 2 hours followed by overnight digestion at 65^o^C. The samples were cooled and diluted 20-fold into the autosampler at a final concentration of 10 mg/mL.

### Drug loading for BSNRs

BSNRs-RPV formulations were prepared by using a similar technique as for lipid coating of ^177^LuBSNRs. First, 10 mg of RPV free base was added to a glass vial and completely dissolved in methanol with sonication for 10-15 minutes. Second, 100 mg L-α-Phosphatidylcholine and 50 mg of DSPE-PEG_2000_ were placed in a round bottom flask, chloroform and hexane were added along with RPV in same flask. The solvent was removed by rotavaporation to create a uniform thin lipid film. The film was left to dry in a desiccator vacuum overnight to ensure the removal of any trace solvent. Third, 100 mg of ^177^Lu/BSNRs particles were dispersed in cyclohexane and sonicated for 30 min until completely mixed. Then, 10 mL of 2% (v/v) tween 80 was added and the mixture was sonicated to form a turbid emulsion. Fourth, cyclohexane was removed by rotavaporation and afterwards, particles were transferred into the round bottom flask with RPV-lipid film for coating process. The flask was steadily rotated in warm water with gentle sonication to ensured complete dissolution and coating of lipid film. The BSNRs-RPV particles were purified by centrifugation of the suspension at 270 xg (500 rpm) for 10 minutes. Supernatant containing particles was used for particle size, drug loading quantitation and radioactivity analysis. Metal content was analyzed by ICP-MS. For drug quantification from nanoparticles, ~50 mg of lyophilized nanoparticles was dissolved in 10 mL of DCM: methanol (1:1 v/v) mixture. The mixture was then bath sonicated for 30 minutes followed by centrifugation at 35,1315× g for 30 minutes. The supernatant was collected and DCM was evaporated at room temperature. The drug in methanol was quantified by UPLC-UV/Vis.^24^

### Radiolabeled particle stability

The *in vitro* stability of ^177^LuBSNRs was determined in phosphate-buffered saline (PBS) and rat plasma. Briefly, 100 μL of ^177^LuBSNRs (1.85 Mbq = ~50 μCi) were incubated in 1.5 mL of PBS or rat plasma at 4ºC or 37ºC for up to 72 hours. After 0, 24, 48, and 72 hours, the samples were centrifuged at 2,0817 xg for 30 minutes at 10ºC (Refrigerated Centrifuge 5417R, Eppendorf North America, Hauppauge, NY, USA). The supernatant was collected into a separate microcentrifuge tube. The radioactivity in the pellet and the supernatant were measured by gamma counter. To determine percent stability the following equation was used; Radiolabeling stability (%) = radioactivity in pellet/total radioactivity (pellet plus supernatant).^33^

### Macrophage uptake and retention of particles

The uptake and retention of BSNRs-RPV particles were evaluated in human monocyte-derived macrophages (MDM) following previously published protocols. 24, 33, 35 Briefly, 1.5 x 10^6^ human monocytes were seeded into 12-well polystyrene tissue culture plates and incubated in Dulbecco's Modified Eagles Medium containing macrophage colony stimulating factor for seven days at 37ºC to promote cell differentiation into macrophages (MDM).^24^, ^33^, ^35^ To determine uptake, MDM were treated with BSNRs-RPV at a concentration of either 12.5 μg/mL or 25 μg/mL (bismuth content) for 2, 4, 6, or 8 hours. To determine cell retention, MDM were treated with either 12.5 μg/mL or 25 μg/mL BSNRs-RPV for 8 hours. The treatment media was removed, cells were washed three times with PBS, and treatment-free media was added. Bismuth and RPV content in cells were determined at 1, 2, 3, and 5 days following treatment with half media exchanges every other day to maintain cell viability. For both uptake and retention, at the specified time points, media was removed, cells were washed three times with PBS, harvested, and counted, following centrifugation at 450 xg for 10 minutes. Cell pellets were stored at -20ºC until analysis. For metal analysis, the cell pellet was dissolved in fresh diluted nitric acid. Bismuth concentration was determined by ICP-MS and reported as µg/10^6^ cells. For RPV analysis, 100 μL of methanol was added to the cell pellet followed by 15 seconds of sonication. The mixture was then centrifuged at 450 xg for 10 minutes and the supernatant collected and analyzed by UPLC-MS/MS for RPV content.^24^, ^33^, ^35^

Cell changes associated with uptake of BSNRs were assessed by TEM, AFM and confocal microscopy, taking advantage of BSNRs intrinsic near infrared (NIR) fluorescence properties. For confocal studies, MDM were cultured on glass coverslips in 12 well plates at a density of 1.5 x 10^6^ cells/well. MDM were then treated with 25 μg/mL BSNRs (as bismuth) for 8 hours. After particle incubation, treatment media was removed and cells were washed three times in cold PBS. After washing, the cells were fixed with ice-cold 4% paraformaldehyde (PFA) in PBS at room temperature for 30 minutes. Glass coverslips with fixed cells were then transferred to glass slides. Cell nuclei were stained with ProLong Gold AntiFade and 4'6-diamidino-2-phenylindole (DAPI, Thermo-Fischer Scientific, Waltham MA, USA) and images acquired using a 63x oil objective on an LSM 710 confocal microscope (Carl Zeiss Microimaging, Inc., Dublin, CA, USA) interfaced with Image Browser AIM software (version 4.2). NIR imaging of particle uptake by MDM was performed on an Olympus IX73 inverted microscope with a xenon excitation source and equipped with an Olympus DP80 Digital Camera and cellSens Dimension software. Unstained slides were imaged for autofluorescence with a FITC filter cube, and NIR fluorescence with an ICG filter cube. Emission wavelengths were; DAPI: 455 nm; TexasRED: 615 nm; ICG: 767 nm. For uptake analysis by TEM, cells were scraped, collected from wells, and fixed in 0.1 M Sorenson's phosphate buffer (pH 6.2) containing 2% glutaraldehyde and 2% w/v PFA for 24 hours at 4ºC. TEM sample preparation, staining, and imaging were performed according to previously published protocols.^24^, ^35^

Visualization of the surface topography of BSNR-loaded MDM was evaluated using AFM. Coverslips with fixed cells were removed from wells and mounted on a slide. Images were acquired in the air using an MFP-3DTM system (Asylum Research, Santa Barbara, CA, USA) operating in tapping mode. AFM probes MSNL-F with a nominal spring constant of ∼0.6N/m were used for imaging. Image processing was performed using Femtoscan software (Advanced Technologies Center, Moscow, Russia).^33^

### Assessment of antiretroviral activities

Antiretroviral activities of BSNRs-RPV were evaluated by measures of HIV reverse transcriptase (RT) activity in cell culture medium and HIV-1 p24 antigen staining of cells and compared to the antiretroviral activity of NRPV as previously described with the following modifications.^33^ First, MDM were treated with 12.5 µg/mL or 25 µg/mL (bismuth concentration) for 8 hours. Second, cells were challenged with HIV-1_ADA_ at a multiplicity of infection (MOI) of 0.1 infectious virions per cell for 16 hours on day 1, 3, 5, or 8 after treatment removal. After an additional 10 days, culture supernatant was removed from cells and assessed for progeny virion production by RT activity.^33^ The cells were fixed with 4% PFA for 15 minutes at room temperature and then stained with antibodies against HIV-1 p24 as previously described.^13^, ^20^, ^24^, ^33^ Images were analyzed using a Nikon TE300 microscope with a 20 X magnification objective.

### Tissue biodistribution of particles

To determine whether BSNRs could predict ARV biodistribution, we designed an *in vivo* experiment comprised of six different treatment groups of 15 Balb/c mice per group (10 mice for group 5). Each group received treatment as a single intravenous injection on the first day of the experiment. As shown in **Table 3** and **supplementary Figure S12**, the first and second groups received NRPV at a dose of 45 mg/kg (G1) and 5 mg/kg (G2), respectively. Group 3 received both BSNRs (80 mg/kg, bismuth) and NRPV (45 mg/kg) (G3). RPV loaded BSNRs (BSNRs-RPV) were administered to the fourth group (G4) at a dose of 80 mg/kg bismuth, (~5 mg/kg RPV). Group 5 (G5) received 80 mg/kg bismuth as ^177^LuBSNRs (37 MBq = ~ 1000 µCi) for SPECT imaging. The sixth group (G6) received non-radioactive BSNRs at a dose of 80 mg/kg bismuth.

For G1 and G2, which received only NRPV, plasma was collected on days 1, 2, 3, 4, 7, 14, 21, and 28 and analyzed by UPLC-MS/MS for RPV content. Out of 15 mice, 5 each were sacrificed and internal organs collected on days 2, 5, and 28. These tissues were analyzed by UPLC-MS/MS for RPV content. G3 and G4, which received BSNRs plus NRPV or BSNRs-RPV, respectively, underwent similar plasma and tissue collection and RPV analysis as G1 and G2, however, plasma and tissue were also analyzed by ICP-MS for bismuth content. G5 received ^177^LuBSNRs and SPECT imaging was performed on these animals at 6, 12, 24, 48, and 120 hours (5 days) post-injection. Acquired images were reconstructed to 3D renditions and regions of interest (ROIs) were drawn over various organs to determine radioactivity concentration in counts per minute (CPM)/mm^3^ tissue. On days 2 and 5 post-injection, 5 animals were sacrificed and liver, spleen, heart, lungs, brain, muscle, blood, bone, lymph nodes, stomach, pancreas, small intestine, large intestine, bladder, testes, kidneys, and tail (injection site) were collected. These organs along with the animals' bedding (excreted radioactivity) and carcasses were subjected to gamma counting (gamma scintillation spectrometry (Ortec NaI(Ti) Scintillation Radiation Detector, Ametek, Oak Ridge, TN, USA) and percent injected dose per gram of tissue (%ID/g) was determined. G6, which received non-radioactive BSNRs, had tissues collected on days 2, 5, and 28. Samples were analyzed for bismuth content by ICP-MS.

Various parameters were assessed across groups and time to determine the predicative value for long-term biodistribution. Pearson's correlation coefficients for two parameter comparisons were determined for RPV concentration, bismuth concentration, ROIs (SPECT) measurements, as well as gamma counter radioactivity measures in liver, spleen and lymph nodes. Means of absolute “r” values with p ≤ 0.05 were used to create the “multiple ribbon bar graph” to summarize the closed correlation groups by including time, dose, treatments, and measurements of particle components.

### SPECT and gamma scintillation determinations biodistribution

SPECT imaging was performed on G5 mice to assess the *in vivo* biodistribution of ^177^LuBSNRs. Each animal received ~1000 μCi of ^177^LuBSNRs (~ 80 mg/kg bismuth). Images were acquired at 6, 12, 24, 48, and 120 hours post injection using a SPECT/CT system (Flex Triumph, TriFoil Imaging, Northridge, CA, USA) fitted with a five pinhole (1.0 mm per pinhole) collimator. First, CT images were acquired using 360 projections over 360° with an X-ray tube current of 140 mA and voltage of 75 kilovoltage peak (kVp) at a magnification of 2.0 (field of view = 59.2 mm^2^). Immediately after, SPECT image acquisition was performed with the following parameters; 64 projections at 15 second per projection over 360° using a radius of rotation of 48 mm (field of view = 59 mm^2^). Co-registration of anatomical CT images and functional SPECT was performed by 3D visualization and analysis software, VivoQuant 3.5 (Invicro Boston, MA, USA). ROIs were drawn over various organs and radioactivity content and organ volume were determined to calculate counts per cubic millimeter (mm^3^). After days 2 and 5 scans, mice were sacrificed and various organs including heart, lungs, liver, pancreas, stomach, spleen, small intestine, large intestine, kidneys, bladder, lymph nodes, muscle, bone, brain, testes, injection site (tail), remaining carcass and blood were collected, weighed, and analyzed *ex-vivo* for radioactivity using gamma counter scintillation spectrometry (ORTEC NaI(Ti) Scintillation Radiation Detector, Ametek, Oak Ridge, TN, USA). Additionally, animals were individually caged so that excreted signal could be measured from the bedding. Signal measured was back-calculated to the time of injection to account for radioactive decay of ^177^Lu (t_1/2_ = 6.64 days).^33^ The signal from each tissue was normalized to the sum of total counts from all sources and then divided by the weight of the organ to obtain % ID/g for each tissue.

### Laser ablation inductively coupled plasma mass spectrometry (LA-ICP-MS)

After G3, G4 and G6 animals were sacrificed, spleens were collected for LA-ICP-MS analysis. Spleen processing and imaging analysis were completed as described previously.^38^, ^107^ In brief, frozen spleens were sliced to a thickness of 20 μm using a LEICA CM1850 cryostat and placed on glass slides for either LA-ICP-MS imaging or hematoxylin and eosin staining. The staining was performed using a rapid Chrome frozen section staining kit (Thermo Fisher Scientific, Waltham, MA, USA). For LA-ICP-MS, a CETAC LSX-213 G2 laser system and a Perkin Elmer Nexion 300X ICP-MS were used. The parameters used in the laser ablation system were the following: spot size = 50 μm, scan rate of 15 μm/s, 10 Hz laser frequency, 10 s of shutter delay, and a carrier He gas flow of 0.6 L/minute. The ICP-MS parameters were the following: nebulizer argon flow rate = 0.7 L/minute, plasma argon flow rate = 16.5 L/minute, auxiliary argon flow rate = 1.4 L/minute, analog stage voltage = -1650 V, and pulse stage voltage = 1000 V. Three elements were detected, including bismuth, iron, and zinc. Images were reconstructed and analyzed in a custom script written in Python that uses multiple metals to identify sub-organ regions of interest. In short, the script uses statistical k-mean clustering to distinguish the red pulp, white pulp, and marginal zones in the spleen, enabling a relative measure of bismuth in each sub-organ region.

### Autoradiography

Livers and spleens were excised at 2 and 5 days post-injection from mice treated with ^177^LuBSNRs (G5). Collected tissues were washed with deionized water and immediately embedded into optimal cutting temperature (O.C.T) compound (Fisher HealthCare, Waltham, MA, USA). Cryostat sections (10 μm) of tissue samples were prepared and exposed to a phosphor screen overnight in the dark (Leica CM1850, Leica Biosystems Inc, Buffalo Grove, IL, USA). The phosphor screen was subsequently imaged by a Typhoon FLA 9500 variable mode imager (GE Lifesciences, Pittsburg, PA, USA) at 25 μm resolution.

### UPLC-MS/MS analysis of drug in plasma and tissues

Plasma samples were prepared for UPLC-MS/MS analysis for RPV content according to our previously published work. 33, 71 In brief, 10 µL of internal standard (IS) solution (250 ng/mL indinavir and 500 ng/ml lopinavir; final concentration 25 ng/ml indinavir, 50 ng/ml lopinavir) was added to 25 µl of plasma along with 1 mL of ice-cold MS -grade acetonitrile. After vortexing and centrifugation, supernatants were evaporated and then reconstituted in 100 µL 50% (v/v) methanol in water. For tissue analyses, 100 mg of tissue was homogenized in 4 volumes of 90% (v/v) methanol in water. Standard curves were prepared in blank mouse plasma or tissue in the range of 0.2-2000 ng/mL RPV with 10 µL of IS added. An ACQUITY UPLC-BEH Shield RP18 column (1.7 µm, 2.1 mm x 100 mm) was used for chromatographic separation of 10 µL of plasma or tissue samples using a 7-minute gradient of mobile phase A (7.5 mM ammonium bicarbonate in Optima-grade water adjusted to pH 7 using acetic acid) and mobile phase B (100% Optima-grade methanol) at a flow rate of 0.25 mL/minute. For more exact details please see our previously published work. 33, 71

### Bismuth quantitation

Bismuth concentrations in plasma and tissues were determined in mouse plasma and tissues by ICP-MS metal at the University of Nebraska-Lincoln's Spectroscopy and Biophysics core facility.^33^

### Tissue localization and toxicity

Particle distribution within the spleen and liver from mice treated with BSNRs (G6) was evaluated via TEM. For TEM examination, tissue samples were fixed in TEM buffer at room temperature. Tissue processing and sectioning were performed as previously described.^24^ *In vivo* toxicity of BSNRs-RPV was determined by histological examination. Here, 5 μm sections of paraffin-embedded tissues were fixed on glass slides and stained with H&E. Images were captured using a Nuance EX multispectral imaging system affixed to a Nikon Eclipse E800 microscope (Nikon Instruments, Melville, NY, USA) fitted with a 20X objective. Histopathological assessment was conducted in accordance with the guidelines of the Society of Toxicologic Pathology. Serum chemistry was assessed using a VetScan comprehensive diagnostic profile disc and a VetScan VS-2 instrument (Abaxis Veterinary Diagnostics, Union City, CA, USA).^24^, ^33^, ^35^

### Statistical analyses

For all studies, data were analyzed using GraphPad Prism 8.2 software (GraphPad, La Jolla, CA, USA) and are presented as the mean ± standard error of the mean (SEM). Experiments with multiple time points were analyzed using two-way factorial ANOVA and Bonferroni's post hoc tests for multiple comparisons. Pearson's correlation analyses were performed for multiple tissues, doses, and data acquisition times from SPECT, ICP-MS and UPLC-MS/MS. Two-parameter comparisons yielding a Pearson coefficient greater than 0.5 (r > 0.5) were considered of predictive value.

## Supplementary Material

Supplementary figures and tables.

Supplementary videos.

## Figures and Tables

**Table 1:** Physicochemical characteristics of NRPV and BSNRs-RPV particles.

Characteristics	Formulations			
	NRPV		BSNR-RPV	
Size, nm (DLS)		341.1	205.3	
PDI (DLS)		0.22	0.31	
Core size, nm (TEM)		~287.14 (L) X ~158.75 (W)	~70 (L) X ~12 (W)	
Drug content (%)		~83.0	~ 3.50	

**Table 2:** Radiolabeling stability of ^177^LuBSNR in rat plasma and PBS at 4ºC and 37ºC.

Time points (hours)	^177^LuBSNRs labeling efficiency (%)			
	4°C		37°C	
	PBS	Plasma	PBS	Plasma
0	100	100	100	100
24	88.8 ± 1.0	81.8 ± 1.0	86.9 ± 2.8	80.7 ± 1.3
48	87.1 ± 4.3	60.3 ± 6.1	72.2 ± 3.2	64.7 ± 5.0
72	72.6 ± 1.6	51.2 ± 7.8	66.3 ± 0.6	56.2 ± 0.9

**Table 3:** Real time *iv vivo* drug biodistribution measurement by design of multimodal theranostic and therapeutic nanoparticle study.

Groups	Formulations	*In vivo* test					
		Dose mg/kg		SEPCT/CT	Gamma Counter	UPLC-MS/MS	ICP-MS
		RPV	Bi	Time (hour)	Time (day)	Time (day)	Time (day)
G1	NRPV-High	45	--	--	--	1,2,3,5,7,14,21,28	
G2	NRPV-Low	5	--	--	--	1,2,3,5,7,14,21,28	
G3	BSNRs + NRPV	45	80	--	--	2,5,28	2,5,28
G4	BSNRs-PRV	5	80	--	--	2,5,28	2,5,28
G5	^177^LuBSNRs	~1000 µCi	~80	6,12,24,48, 120	2, 5	--	--
G6	BSNRs	--	80	--	--	--	2,5,28

## Abbreviation

^177^Lu: ^177^Lutetium
^177^LuBSNRs: ^177^Lutetium-bismuth sulfide nanorods
AFM: atomic force microscopy
ART: antiretroviral therapy
ARV: antiretroviral drugs
Bq: becquerel
BSA: bovine serum albumin
BSNRs: bismuth sulfide nanorods
BSNRs-RPV: bismuth sulfide nanorods rilpivirine
Ci: curie
DAPI: 4'6-diamidino-2-phenylindole
DCM: dichloromethane
DMSO: dimethyl sulfoxide
DSPE-PEG2000: 1,2-distearoyl-sn-glycero-3-phosphoethanolamine-N-[amino(polyethylene glycol)-2000]
EC50: 50% maximal effective concentration
GALT: gut-associated lymphoid tissue
HIV-1: human immunodeficiency virus type 1
HR-TEM: high-resolution transmission electron microscopy
IV: intravenous
IM: intramuscular
kVp: kilovoltage peak
LA ARV: long acting antiretroviral drugs
LA-ICP-MS: laser ablation inductively coupled plasma mass spectrometry
LA NRPV: long acting nanoformulated rilpivirine
LASER ART: long acting slow effective release antiretroviral therapy
MBq: mega becquerel
MDM: human monocyte-derived macrophage
MOI: multiplicity of infection
MTT: 3-(4,5-dimethylthiazol-2-yl)-2,5-diphenyltetrazolium bromide
NanoART: nanoformulated antiretroviral therapy
NIR: near Infrared
NRPV: nanoformulated rilpivirine
OA: oleic acid
OAm: oleyl amine
PBS: phosphate-buffered saline
PC: phosphatidylcholine
PD: pharmacodynamics
PDI: polydispersity index
PFA: paraformaldehyde
PK: pharmacokinetic
RB: round-bottom flask
ROI: region of interest
RPV: rilpivirine
RT: reverse transcriptase
SAED: selected area diffraction
SEM: scanning electron microscope
SPECT: single photon emission computed tomography
STEM: Scanning transmission electron microscopy
TEM: transmission electron microscopy
μCi: microcurie
UPLC-MS/MS: ultra-performance liquid chromategraphy tandem mass spectrometry
UPLC: liquid chromatography
XRD: x-ray diffraction
XRF: x-ray fluorescence
XPS: x-ray photoelectron spectroscopy
