# Supplementary material for: Rod-shape theranostic nanoparticles facilitate antiretroviral drug biodistribution and activity in human immunodeficiency virus susceptible cells and tissues
Source: Theranostics. 2020 Jan 1;10(2):630–56. doi: 10.7150/thno.39847 (PMC6929995; doi:10.7150/thno.39847)
Supplement: Supplementary file 1 — Supplementary figures and tables. [file thnov10p0630s1.pdf]

<u>*Supporting Data*</u>

Rod-shape theranostic nanoparticles facilitate antiretroviral drug biodistribution and activity in human immunodeficiency virus susceptible cells and tissues

Bhavesh D. Kevadiya[1], Brendan Ottemann[1], Insiya Z. Mukadam[2], Laura Castellanos[3], Kristen Sikora[3], James R. Hilaire[1], Jatin Machhi[1], Jonathan Herskovitz[1,4], Dhruvkumar Soni[2], Mahmudul Hasan[2], Wenting Zhang[2], Sarella Anandakumar[5], Jered Garrison[2], JoEllyn McMillan[1], Benson Edagwa[1], R. Lee Mosley[1], Richard W. Vachet[3] and Howard E. Gendelman[1,2,4]✉

1. Department of Pharmacology and Experimental Neuroscience, College of Medicine, University of Nebraska Medical Center, NE, USA
2. Department of Pharmaceutical Sciences, College of Pharmacy, University of Nebraska Medical Center, NE, USA
3. Department of Chemistry, University of Massachusetts, Amherst, MA, USA
4. Department of Pathology and Microbiology, College of Medicine, University of Nebraska Medical Center, NE, USA
5. Nebraska Center for Materials and Nanoscience, University of Nebraska-Lincoln, NE, USA

✉ Corresponding author: Howard E. Gendelman, M.D., Department of Pharmacology and Experimental Neuroscience, University of Nebraska Medical Center, Omaha, NE 68198; phone 402 559 8920; FAX: 402 559 3744; Email: hegendel@unmc.edu

Results

SR1: XPS Analysis

BSNRs and LuBSNRs particles were analysis by XPS and data were shown in the survey plot **(supplementary Figure S5A, E)** exhibiting the present of key elements, such as O, C, Bi, and S. This shows that the particle surface is of the highest quality in terms of chemistry and stability, free from unreacted end precursors. The presence of the O peak is due to the adsorbed oxides on the particles surface.[1] C1s with standard binding energy value of ~ 285 eV is used as a reference.[2] In the Bi region, the high resolution XPS spectra **(supplementary Figure S5C)** validations two characteristic peaks at 158.3 and 163.6 eV, which can be exchangeable, respectively, to the binding energies of Bi 4f7/2 and Bi 4f5/2 in BSNRs particles.[2, 3] The broad peak at around 225.43 eV **(supplementary Figure S5B)** corresponds to the binding energy of S2s. The experimental values are analyzed to be in nearby with the data reported previously. [4] LuBSNRs particles' most intense peak occurs at 157.8 eV (Bi 4f7/2). This peak and corresponding doublet at 163.1 eV (Bi 4f5/2) indicates that the Bi/S ratio in the compound is greater at the surface or is indicative of BiS coordination. Lu signals appeared but did not rise above the level of the background signal which indicates Lu was not available on the surface of particles **(supplementary Figure S5H)**.

SR2: Morphology and particle size characterization

The surface morphology of BSNR particles was studied using AFM. The size and size distribution of these BSNRs demonstrated uniform size and distribution (**Figure S6A-B**). Surface morphology of the NRPV nanocrystals was determined using AFM, SEM and

TEM. The particles were uniform and monodisperse with a characteristic rectangular shape. SEM images of pure NRPV nanocrystals are shown in **Figure S6C-E**.

***SR3: In vitro* bismuth and RPV correlation study in macrophage**

Figure S9: Panels A-D show various two parameter comparisons for data obtained for bismuth and RPV concentrations in macrophages treated with BSNRs-RPV particles at 12.5 µg/mL or 25.0 µg/mL concentrations. **Panel A** shows the correlation, between the uptake of bismuth, after macrophages were treated with BSNRs-RPV particles at a low concentration (12.5 µg/mL) or at a higher concentration (25 µg/mL). As expected, this correlation was very good with r = 0.9982. We also examined the relationship between the uptake of RPV in macrophages treated with a low (12.5 µg/mL) or high (25 µg/mL) concentration of BSNRs-RPV, and found a correlation with r = 0.8350, as shown in **panel B. Panel C** shows the correlation between bismuth and RPV uptake over time in macrophages treated with either 12.5 µg/mL or 12.5 µg/mL BSNRs-RPV particles, while **panel D** displays the correlation in cells treated with 25 µg/mL BSNRs-RPV particles. The bismuth and RPV data from the lower concentration of BSNRs-RPV particles showed a positive correlation with r = 0.7570 while the higher concentration showed a positive correlation with r = 0.9690. **Panels E-H** show the various two parameter comparisons for data obtained during retention experiments. Macrophages were treated with either a high (25 µg/mL) or low (12.5 µg/mL) concentration of BSNRs-RPV particles for 8 hours before treatment was removed and replaced by treatment free media. The retention of bismuth in macrophages treated with 12.5 µg/mL BSNRs-RPV positively correlated with the amount of RPV retained in cells over time in macrophages treated with 25 µg/mL BSNRs-RPV particles, with r = 0.9721. The comparison between bismuth

concentrations retained in macrophages treated with the high concentration of BSNRs-RPV particles and bismuth concentration retained in cells treated with the lower BSNRs-RPV particles dose yielded an “r” value of 0.9353. **Panel G** shows the relationship between RPV and bismuth retention in cells treated with a low dose of BSNRs-RPV particles. This also demonstrated a significant, positive correlation with r = 0.8093. The final **panel, H,** shows the positive correlation between the two parameters in cells treated with a high concentration of BSNRs-RPV particles (r = 0.8200).

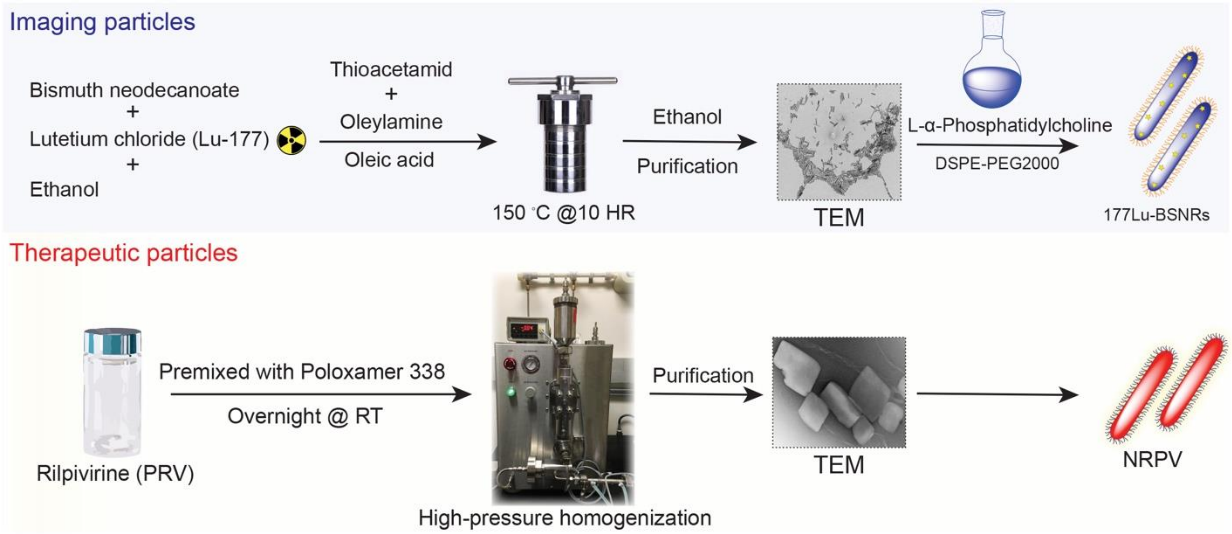

Figure S1. Schematic of production of imaging and therapeutic particles. Schematic illustration of the two-step production, synthesis, and lipid functionalization of BSNRs imaging particles via solvothermal technique. Therapeutic NRPV particles were prepared by a top-down high-pressure homogenization approach using poloxamer 338 as a surfactant and stabilizing agent. Representative transmission electron microscopy (TEM) images of both types of particles are depicted in this schematic.

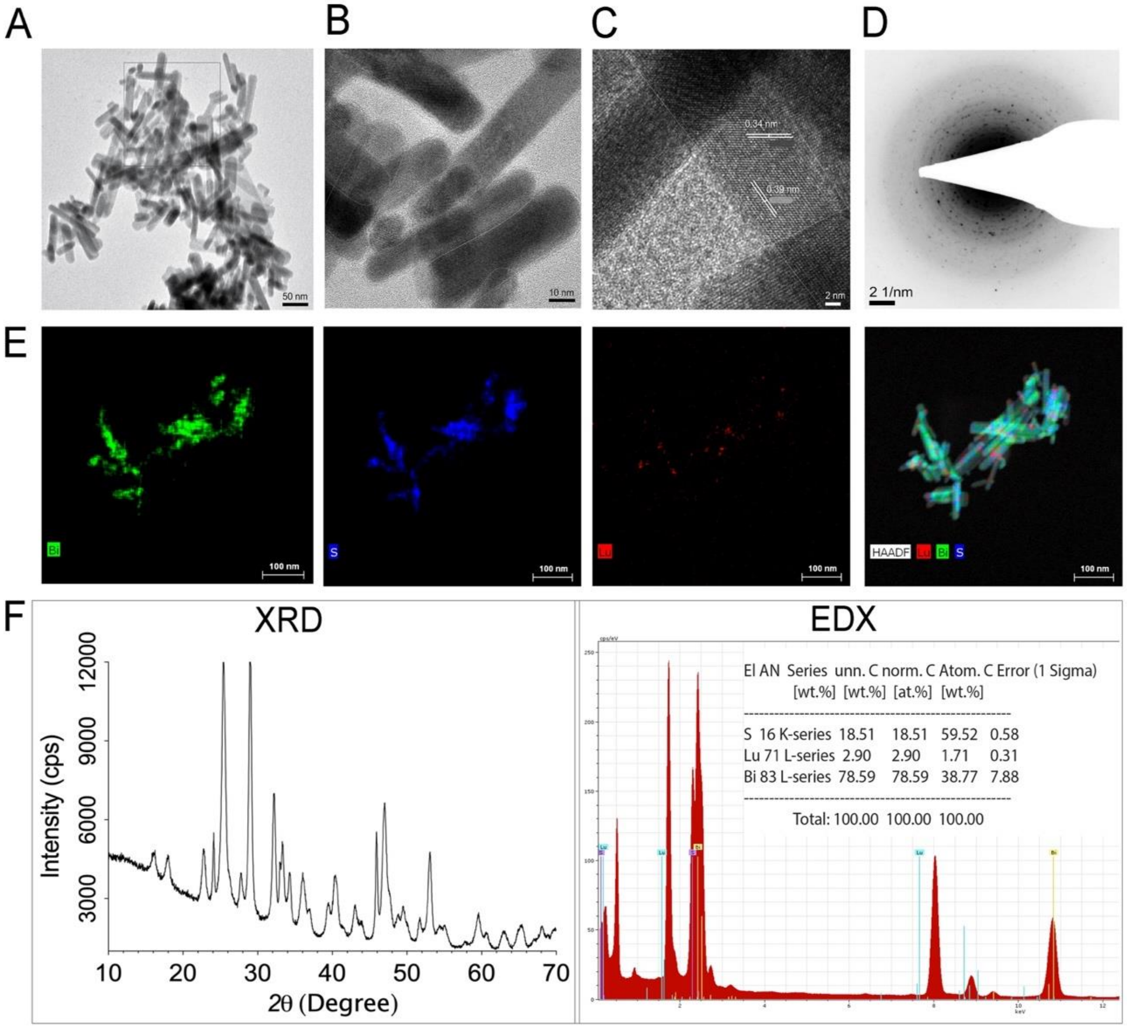

Figure S2. Synthesis and characterization of LuBSNRs. Low resolution TEM images of LuBSNRs are illustrated in (A). High resolution TEM images of twin LuBSNRs particles with lattice planes are shown in (B-C) with corresponding electron diffraction patterns. The selected area electron diffraction (SAED) pattern of LuBSNRs are shown in panel (D). High-angle annular dark-field electron microscopy showed element localization within the particles. Bismuth (green), sulfur (blue) and lutetium (red) (E). XRD patterns (F left panel) and quantitative measurements of particle element distribution were made by energy dispersive X-ray spectroscopy (F, right panel). Chemical composition of LuBSNRs was assessed by X-ray fluorescence. Typical particles consisted of 87.72% bismuth, 11.29% sulfur and 0.975 % lutetium by mass (**XRF data: Supplementary figure S4**). Molar ratio of synthesis was 1:1:0.1 (Bismuth neodecanoate: thioacetamide: lutetium (III) chloride, anhydrous, $LuCl_3$) with a typical synthesis involving, 8 mM (5.8g) bismuth neodecanoate, 8 mM (600 mg) thioacetamide, and 0.8 mM (224 mg) $LuCl_3$.

2019- 6-28 12:04

SQX Calculation Result

Sample : BiS
Date analyzed : 2019- 6-28 11:36
Application : F-U_Solid_N_021
Sample type : Metal & Alloy
Balance :

Matching library :
Sample film corr. :
Impurity corr. : CupFiCot
File : BiS

No.	Component	Result	Unit	Det. limit	El. line	Intensity	w/o normal
1	S	11.3866	mass%	0.06993	S -KA	33.1299	11.6919
2	Bi	88.6134	mass%	0.07634	Bi-LA	152.8823	72.7142

Rigaku

Figure S3. XRF (X-ray fluorescence) analysis of BSNRs.

2019- 6-28 12:03

SQX Calculation Result

Sample : LuBiS
Application : F-U_Solid_N_020　Sample type : Metal & Alloy
Date analyzed : 2019- 6-28 11:15
Balance :

Matching library :
Sample film corr. :
File : LuBiS
Impurity corr. : CupFiCot

No.	Component	Result	Unit	Det. limit	El. line	Intensity	w/o normal
1	S	11.2982	mass%	0.07030	S -KA	33.9524	12.0977
2	Lu	0.9756	mass%	0.07762	Lu-LB1	0.7009	0.8335
3	Bi	87.7262	mass%	0.08037	Bi-LA	156.2487	74.9502

Rigaku

Figure S4. XRF (X-ray fluorescence) analysis of LuBSNRs.

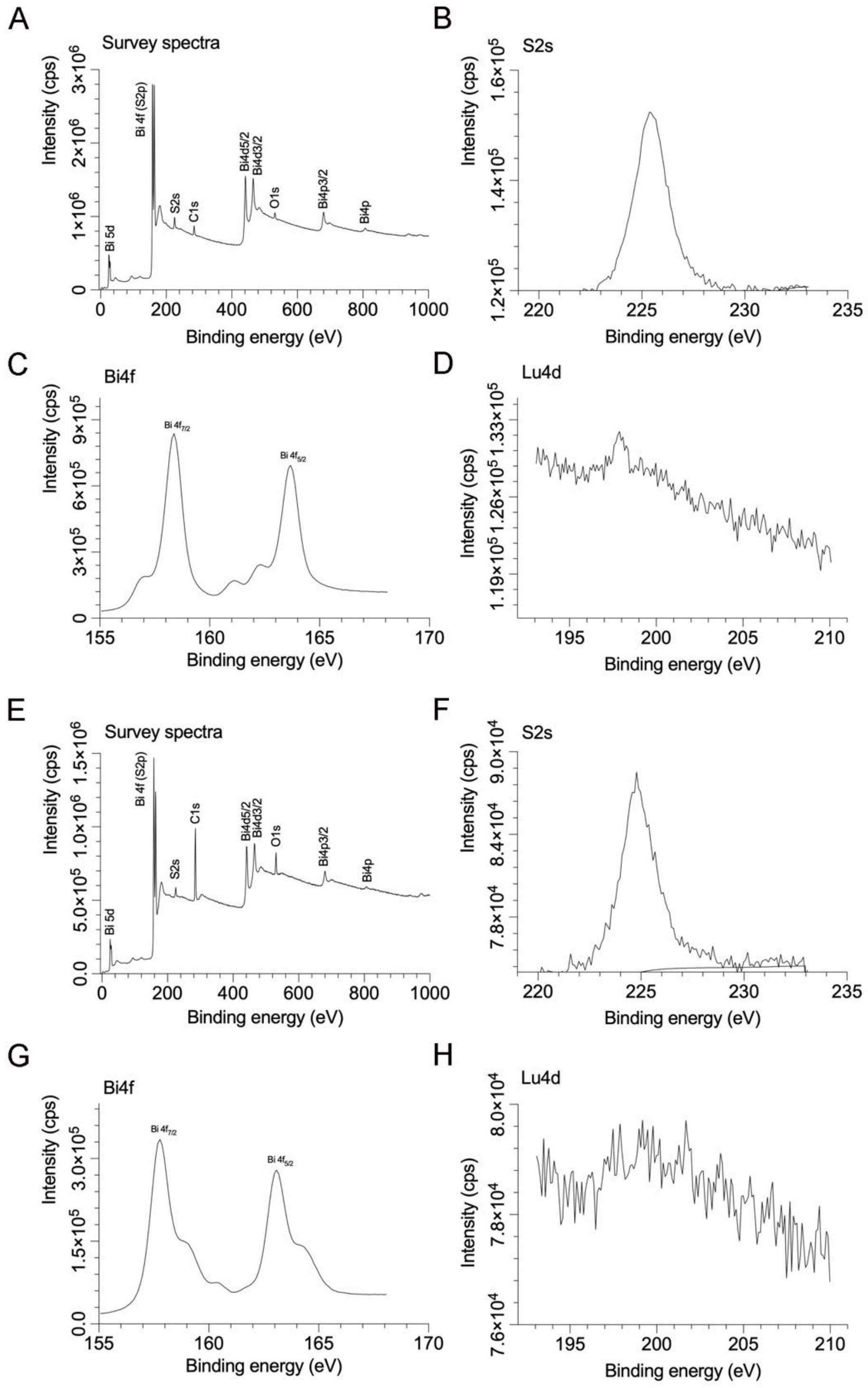

Figure S5. X-Ray Photoelectron Spectroscopy (XPS) analysis of Lu/BSNRs. Survey spectra (A, E), S region, (B, F), Bi (B, F) and Lu regions (D, H) of BSNRs and LuBSNRs particles, respectively are illustrated.

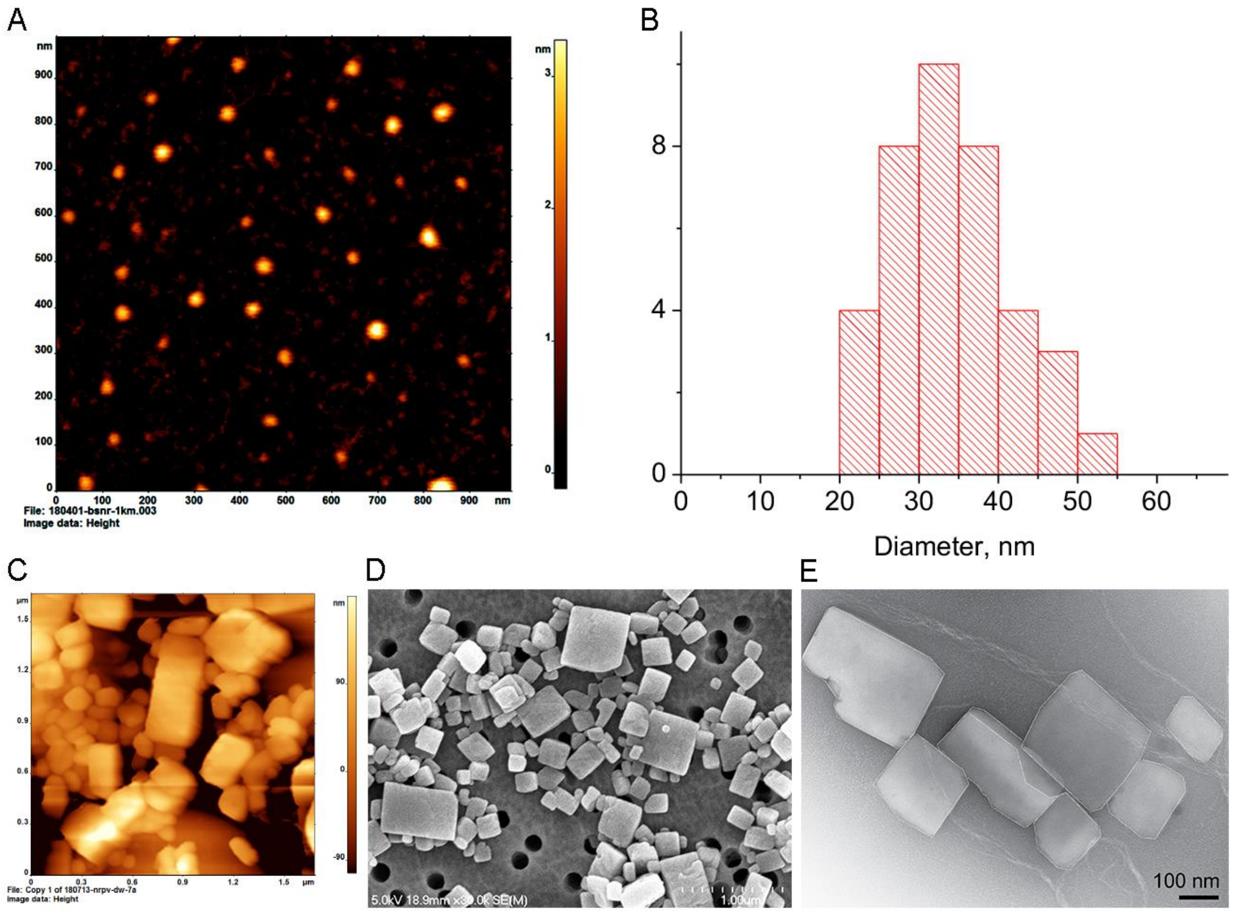

Figure S6. Morphological analysis of particles. AFM image of prepared BSNRs dispersed in cyclohexane (A). Corresponding size distribution histogram of BSNRs particles (B) and NRPV particles (C). SEM (D), and TEM (E) images of the NRPV particles are shown.

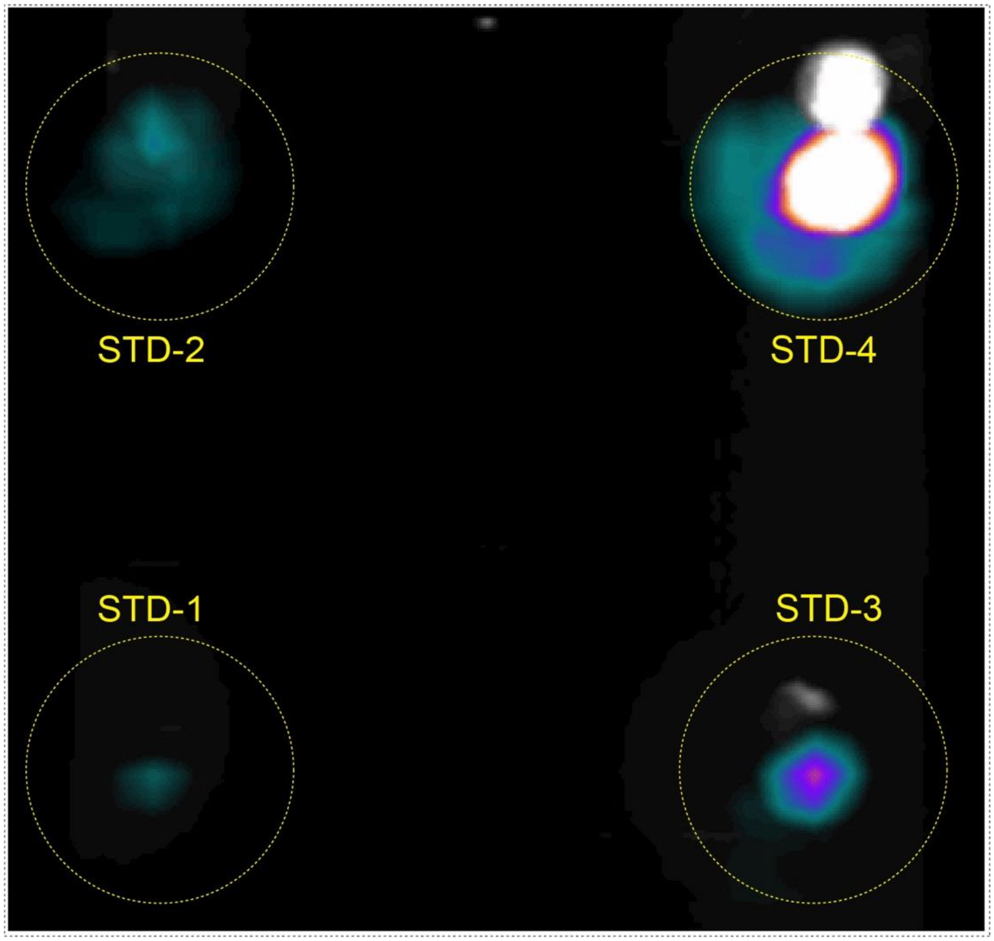

Figure S7. Phantom preparation. SPECT/CT phantom experiment using four known concentrations of ^{177}LuBSNRs particles are illustrated (STD 1 (~12.5 µCi); STD -2 (~ 25 µCi); STD-3 (~30 µCi); STD-4 (~50 µCi).

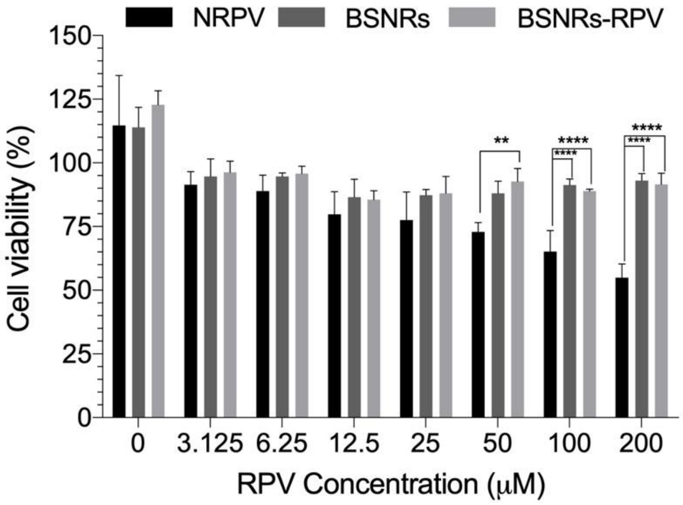

Figure S8. Cell viability. The analyses were performed with 3-(4,5-dimethylthiazol-2-yl)-2,5-diphenyltetrazolium bromide (MTT); a cell viability assay performed in macrophages after treatment with NRPV, BSNRs-RPV or BSNRs particles at various concentrations (RPV). The total time of analysis was 8 hours. Significant differences were determined by a 2-way ANOVA followed by the Tukey's post hoc analysis. Stars denote significant differences compared to NRPV. **$p < 0.01$; ***$p < 0.001$; ****$p < 0.0001$.

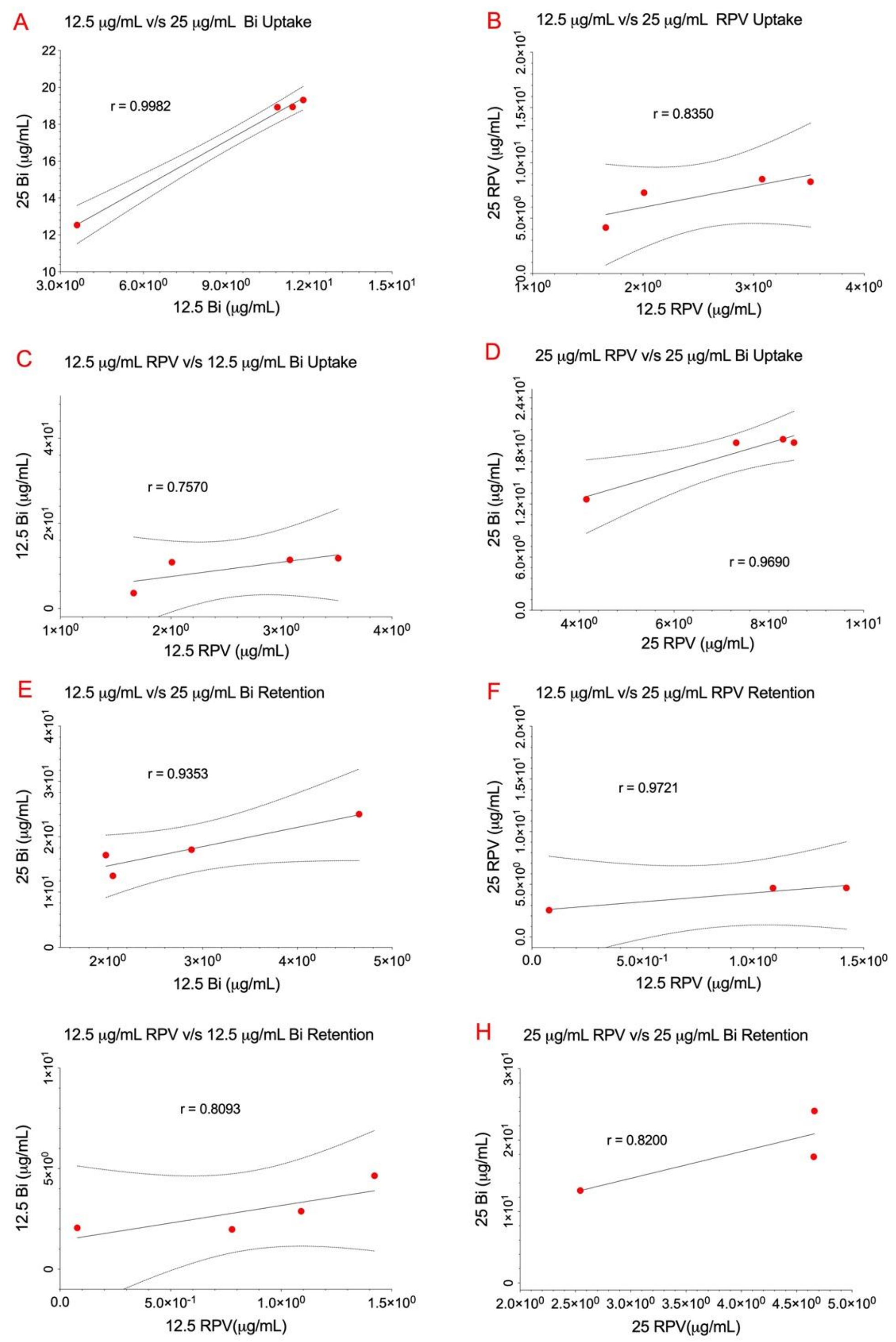

Figure S9. *In vitro* uptake and retention. Cross validation of metal and drug contents in macrophages by Pearson correlation analysis (Note: Panel "A" and "G" were also presented in Figure 2 of the manuscript).

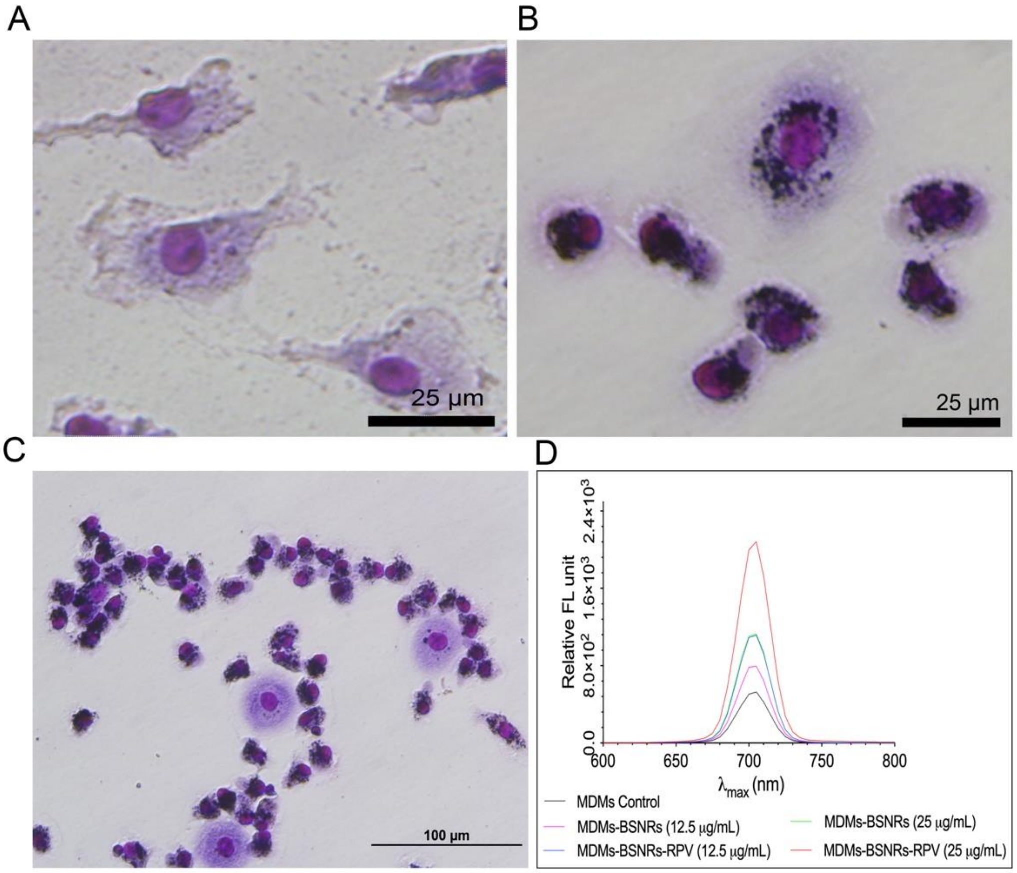

Figure S10. Light microscopy images of BSNR particles in macrophages (Giemsa stain). The black dots observed in the purple (Giemsa) stained cytoplasm are BSNRs particles. Untreated macrophages (A) and particle treated macrophages (25 μg/mL based on bismuth content) (B). Low magnification image of particle treated cells (C). Staining was carried out according to the manufacturer's instructions (Sigma Aldrich, May-Grünwald, MG500-500 mL and Giemsa stain Modified Solution, 48900-100ML-F). Uptake in macrophages was reconfirmed by fluorescence spectra analysis of particle-loaded cells (D).

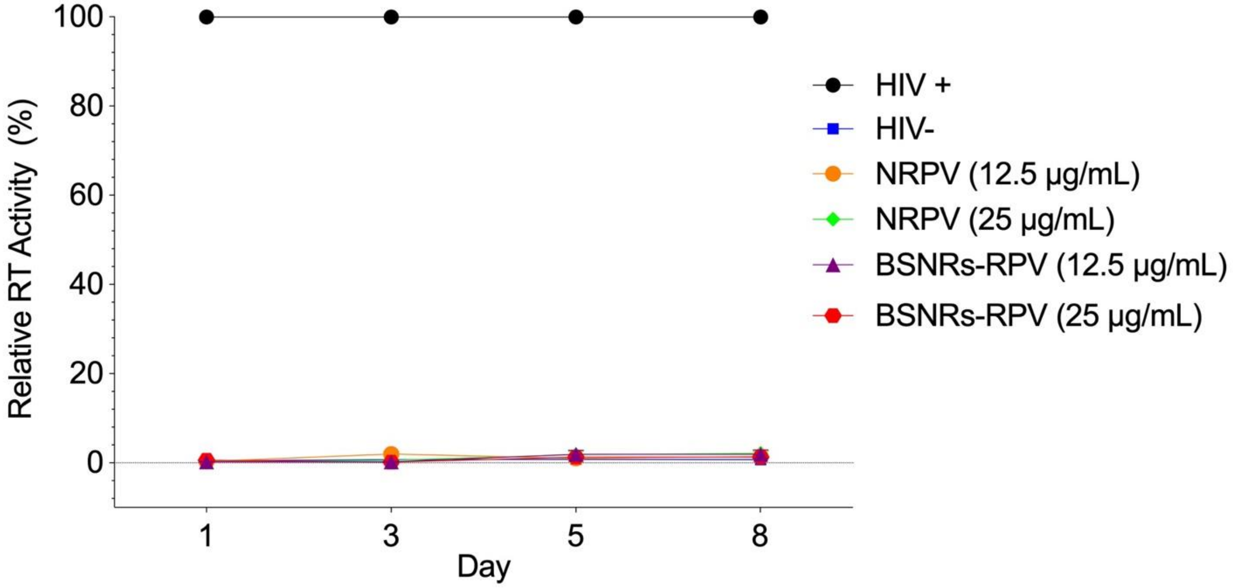

Figure S11. Antiretroviral activity as determined by HIV-1 reverse transcriptase. Antiretroviral activity was determined in macrophages treated for 8 hours with NRPV or BSNRs-RPV particles (12.5 and 25 µg/mL based on Bismuth contents) and then infected with HIV-1_{ADA} 1, 3, 5 or 8 days after particles treatment at a multiplicity of infection (MOI) of 0.1. At 10 days after infection, the media was collected from wells and progeny HIV virion production was determined by HIV reverse transcriptase (RT) activity using beta scintillation spectroscopy as previously described.[5]

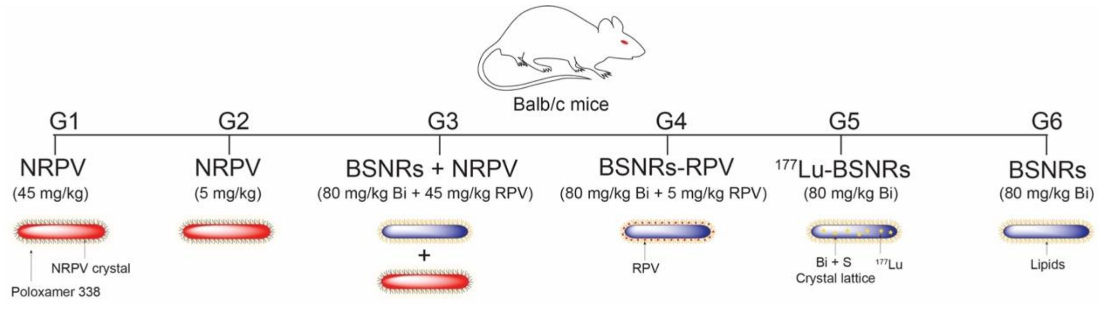

Figure S12. Design of animal experiments with different treatment groups.

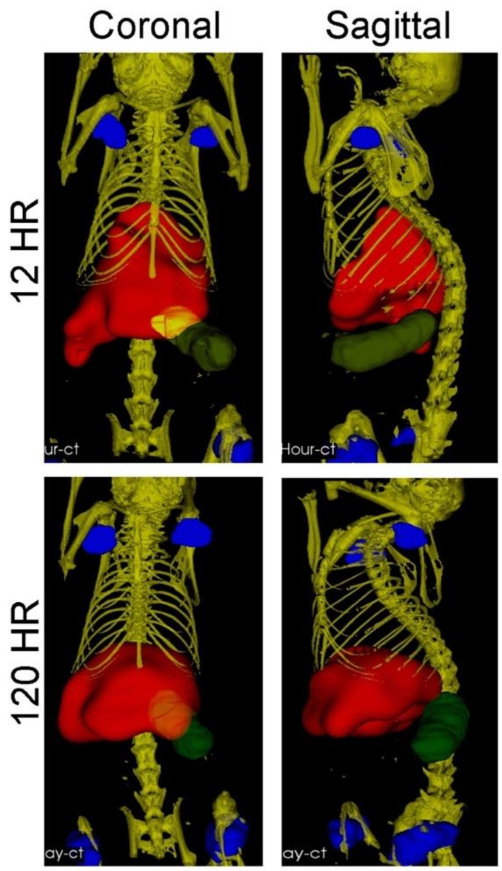

Figure S13. SPECT regions of interest (ROIs). SPECT 3D images of ^{177}LuBSNRs particles injected into Balb/c mice were quantified by drawing 3D regions-of-interest (ROI) to obtain radioactivity counts per volume of tissue. Shown here are representative ROIs for various organs. Regions of interest were drawn using VivoQuant v3.5 (Invicro Imaging Software) based on anatomy. Color code; liver = red, spleen = green, lymph nodes = blue and skeleton = yellow.

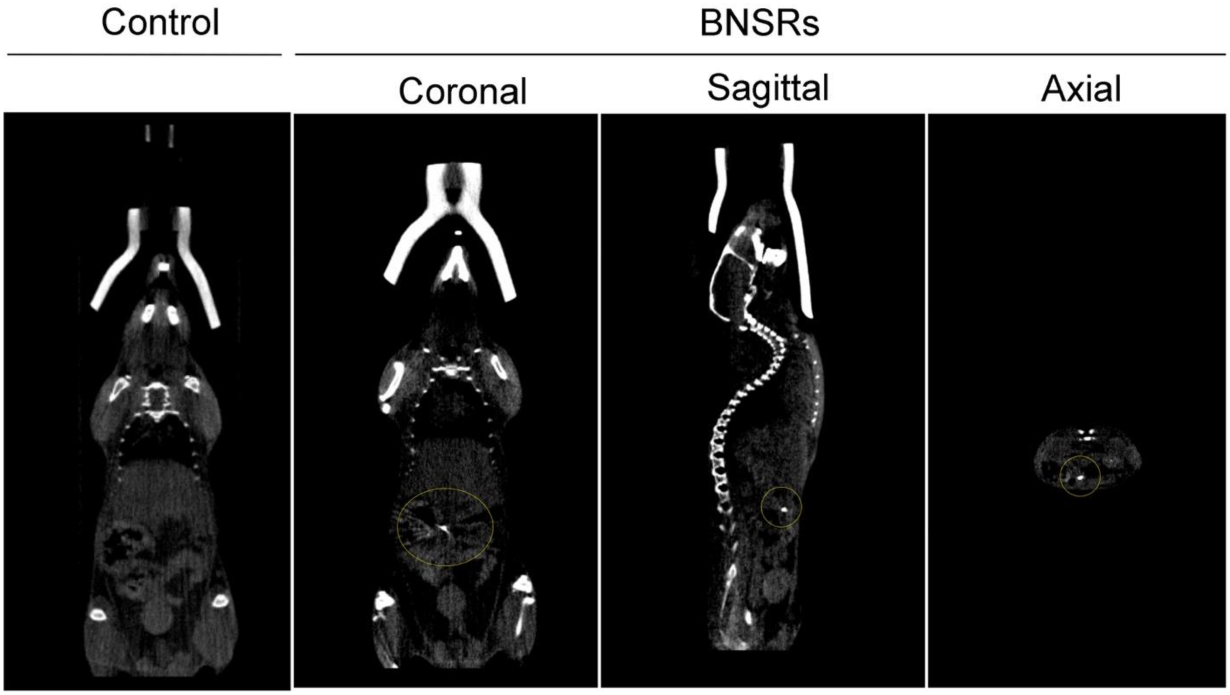

Figure S14. CT imaging using Xstrahl small animal radiation research platform (sarrp). BSNRs provide CT contrast in the system as represented by the images. CT coronal, sagittal and axial views of a mouse following intravenous injection of BSNRs solution (80 mg/kg) are shown. BSNRs treated mouse were shown the small signal for gut area highlighted in yellow ring. Two animals (One injected BSNRs solution at a dose of 80 mg/kg and other untreated) were analyzed 2 days after treatment using Xstrahl Inc. (Xstrahl, Suwanee, GA, USA) small animal radiation research platform (sarrp). Micro CT image is acquired in a pancake geometry. [6] The X-ray tube operates at a focal spot of 1 mm with tube voltage of 60 kV which gives an output of 1cGy of radiation over 1 minute. The images are acquired at a frame rate of 30 fps. After that the images are analyzed using (3D) Slicer with the DICOM module (https://www.slicer.org).

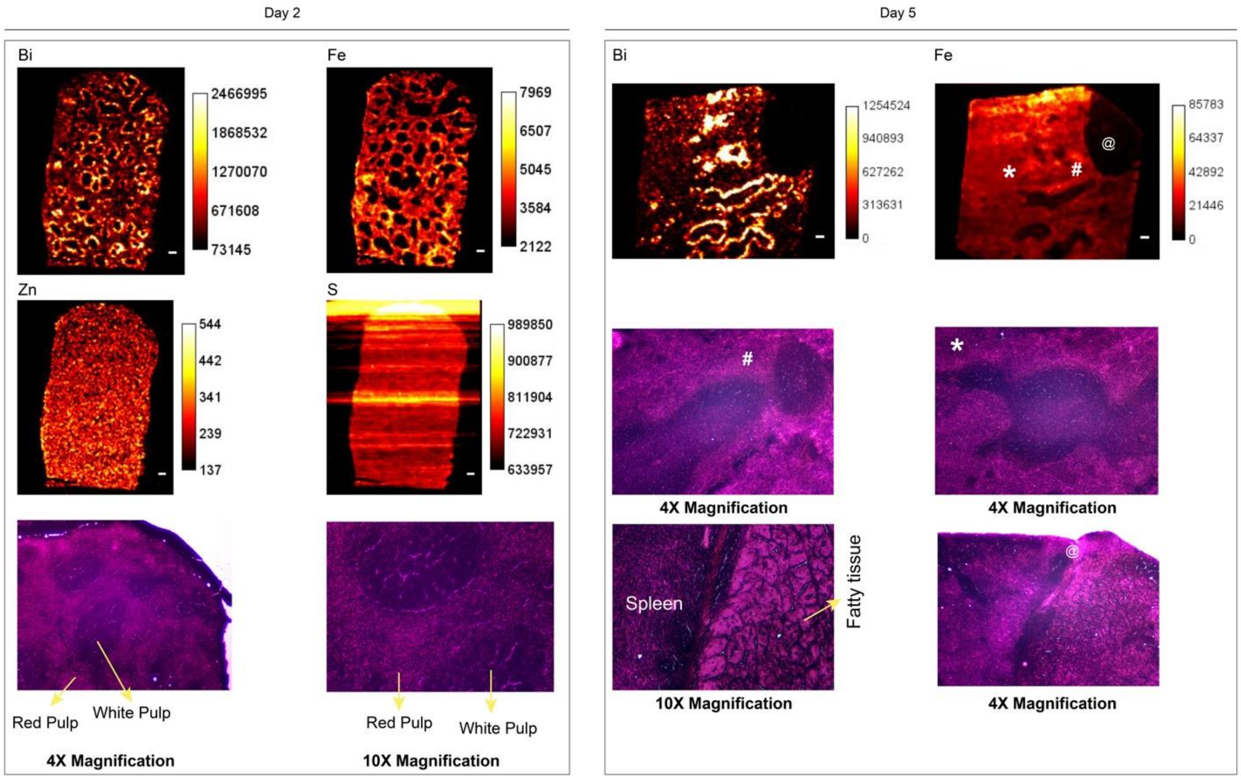

Figure S15. LA-ICP-MS images of BSNRs (G6) in spleen tissue. Spleen tissue from animals treated with BSNRs (G6) was collected on days 2 and 5 post-injection. Tissue sections were stained with H&E and analyzed by LA-ICP-MS. Bi, Fe, Zn and S levels were compared with background levels. Signal to background ratio for Bi was found to be highest in the macrophage rich marginal zone of the spleen tissue and the red pulp region with higher levels seen at day 5 as compared to day 2. Fe allows for distinction between the red and white pulp. Zn is used as an internal standard for signal quality. The white bar corresponds to 10 pixels (500 µm).

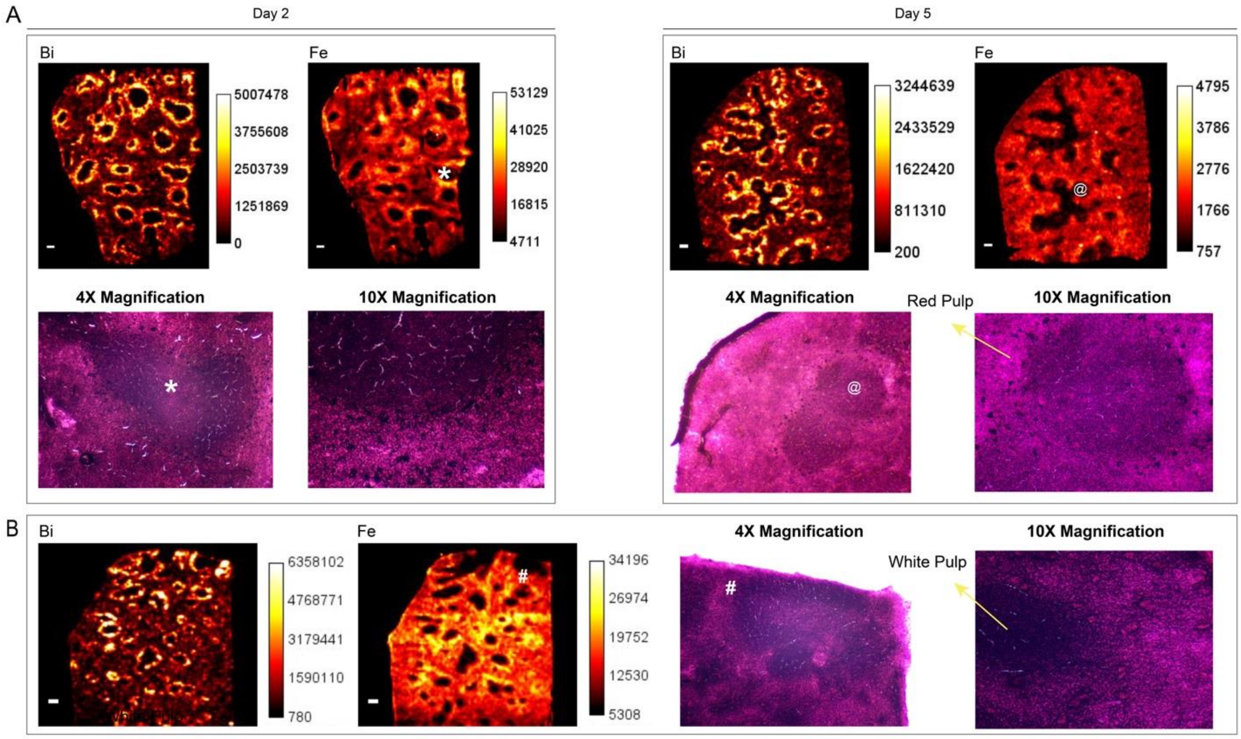

Figure S16. LA-ICP-MS images of BSNRs-RPV (G4) and BSNRs and NRPV(G3) treated spleen tissue. Spleens from animals treated with BSNRs-RPV(G4) were collected on days 2 and 5 after injection (A). Spleen tissue from animals treated with BSNRs plus NRPV(G3) was collected on Day 2 post-injection (B). Tissue sections were stained with H&E and analyzed by LA-ICP-MS. Bi and Fe levels were compared with background levels. Signal to background ratio for Bi was found to be highest in the macrophage rich marginal zone of the spleen tissue and the red pulp region with higher levels seen at day 5 as compared to day 2. Fe allows for distinction between the red and white pulp. The white bar corresponds to 10 pixels (500 µm).

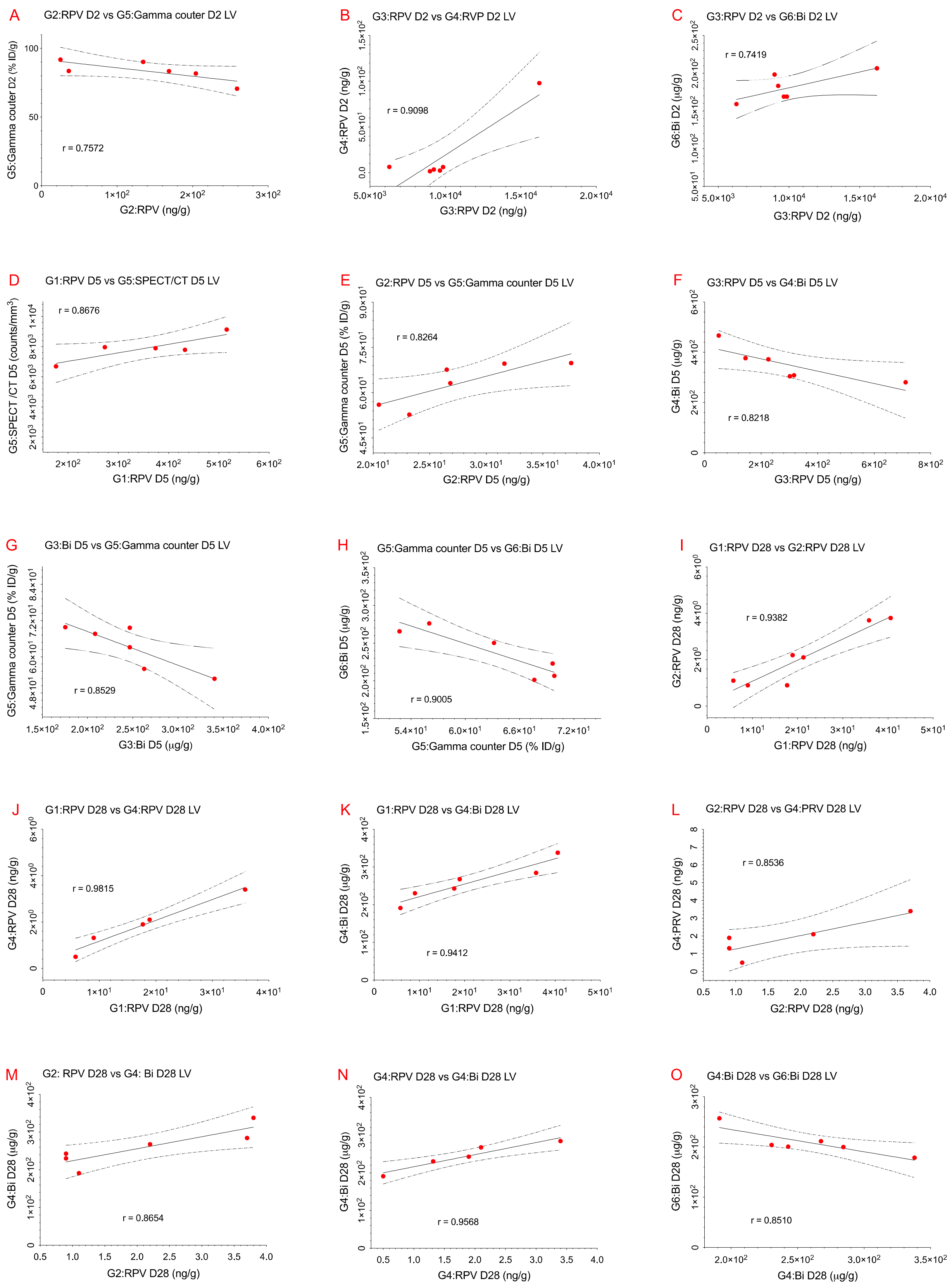
A
G2:RPV D2 vs G5:Gamma couter D2 LV
G5:Gamma couter D2 (% ID/g)
G2:RPV (ng/g)
r = 0.7572
B
G3:RPV D2 vs G4:RVP D2 LV
G4:RPV D2 (ng/g)
G3:RPV D2 (ng/g)
r = 0.9098
C
G3:RPV D2 vs G6:Bi D2 LV
G6:Bi D2 (µg/g)
G3:RPV D2 (ng/g)
r = 0.7419
D
G1:RPV D5 vs G5:SPECT/CT D5 LV
G5:SPECT /CT D5 (counts/mm³)
G1:RPV D5 (ng/g)
r = 0.8676
E
G2:RPV D5 vs G5:Gamma counter D5 LV
G5:Gamma counter D5 (% ID/g)
G2:RPV D5 (ng/g)
r = 0.8264
F
G3:RPV D5 vs G4:Bi D5 LV
G4:Bi D5 (µg/g)
G3:RPV D5 (ng/g)
r = 0.8218
G
G3:Bi D5 vs G5:Gamma counter D5 LV
G5:Gamma counter D5 (% ID/g)
G3:Bi D5 (µg/g)
r = 0.8529
H
G5:Gamma counter D5 vs G6:Bi D5 LV
G6:Bi D5 (µg/g)
G5:Gamma counter D5 (% ID/g)
r = 0.9005
I
G1:RPV D28 vs G2:RPV D28 LV
G2:RPV D28 (ng/g)
G1:RPV D28 (ng/g)
r = 0.9382
J
G1:RPV D28 vs G4:RPV D28 LV
G4:RPV D28 (ng/g)
G1:RPV D28 (ng/g)
r = 0.9815
K
G1:RPV D28 vs G4:Bi D28 LV
G4:Bi D28 (µg/g)
G1:RPV D28 (ng/g)
r = 0.9412
L
G2:RPV D28 vs G4:PRV D28 LV
G4:PRV D28 (ng/g)
G2:RPV D28 (ng/g)
r = 0.8536
M
G2: RPV D28 vs G4: Bi D28 LV
G4:Bi D28 (µg/g)
G2:RPV D28 (ng/g)
r = 0.8654
N
G4:RPV D28 vs G4:Bi D28 LV
G4:Bi D28 (µg/g)
G4:RPV D28 (ng/g)
r = 0.9568
O
G4:Bi D28 vs G6:Bi D28 LV
G6:Bi D28 (µg/g)
G4:Bi D28 (µg/g)
r = 0.8510

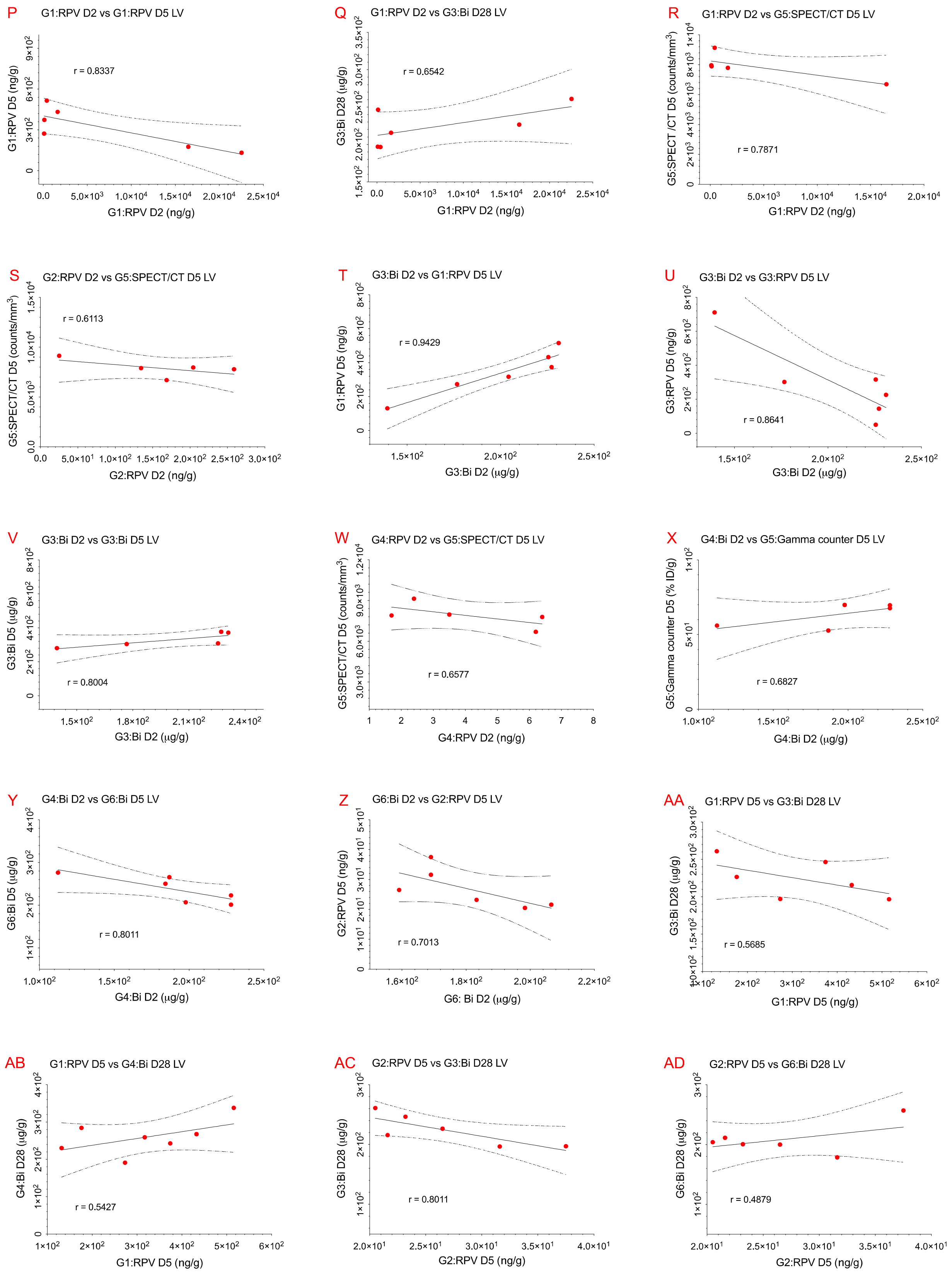
P
G1:RPV D2 vs G1:RPV D5 LV
r = 0.8337
G1:RPV D5 (ng/g)
G1:RPV D2 (ng/g)
Q
G1:RPV D2 vs G3:Bi D28 LV
r = 0.6542
G3:Bi D28 (μg/g)
G1:RPV D2 (ng/g)
R
G1:RPV D2 vs G5:SPECT/CT D5 LV
r = 0.7871
G5:SPECT /CT D5 (counts/mm3)
G1:RPV D2 (ng/g)
S
G2:RPV D2 vs G5:SPECT/CT D5 LV
r = 0.6113
G5:SPECT/CT D5 (counts/mm3)
G2:RPV D2 (ng/g)
T
G3:Bi D2 vs G1:RPV D5 LV
r = 0.9429
G1:RPV D5 (ng/g)
G3:Bi D2 (μg/g)
U
G3:Bi D2 vs G3:RPV D5 LV
r = 0.8641
G3:RPV D5 (ng/g)
G3:Bi D2 (μg/g)
V
G3:Bi D2 vs G3:Bi D5 LV
r = 0.8004
G3:Bi D5 (μg/g)
G3:Bi D2 (μg/g)
W
G4:RPV D2 vs G5:SPECT/CT D5 LV
r = 0.6577
G5:SPECT/CT D5 (counts/mm3)
G4:RPV D2 (ng/g)
X
G4:Bi D2 vs G5:Gamma counter D5 LV
r = 0.6827
G5:Gamma counter D5 (% ID/g)
G4:Bi D2 (μg/g)
Y
G4:Bi D2 vs G6:Bi D5 LV
r = 0.8011
G6:Bi D5 (μg/g)
G4:Bi D2 (μg/g)
Z
G6:Bi D2 vs G2:RPV D5 LV
r = 0.7013
G2:RPV D5 (ng/g)
G6: Bi D2 (μg/g)
AA
G1:RPV D5 vs G3:Bi D28 LV
r = 0.5685
G3:Bi D28 (μg/g)
G1:RPV D5 (ng/g)
AB
G1:RPV D5 vs G4:Bi D28 LV
r = 0.5427
G4:Bi D28 (μg/g)
G1:RPV D5 (ng/g)
AC
G2:RPV D5 vs G3:Bi D28 LV
r = 0.8011
G3:Bi D28 (μg/g)
G2:RPV D5 (ng/g)
AD
G2:RPV D5 vs G6:Bi D28 LV
r = 0.4879
G6:Bi D28 (μg/g)
G2:RPV D5 (ng/g)

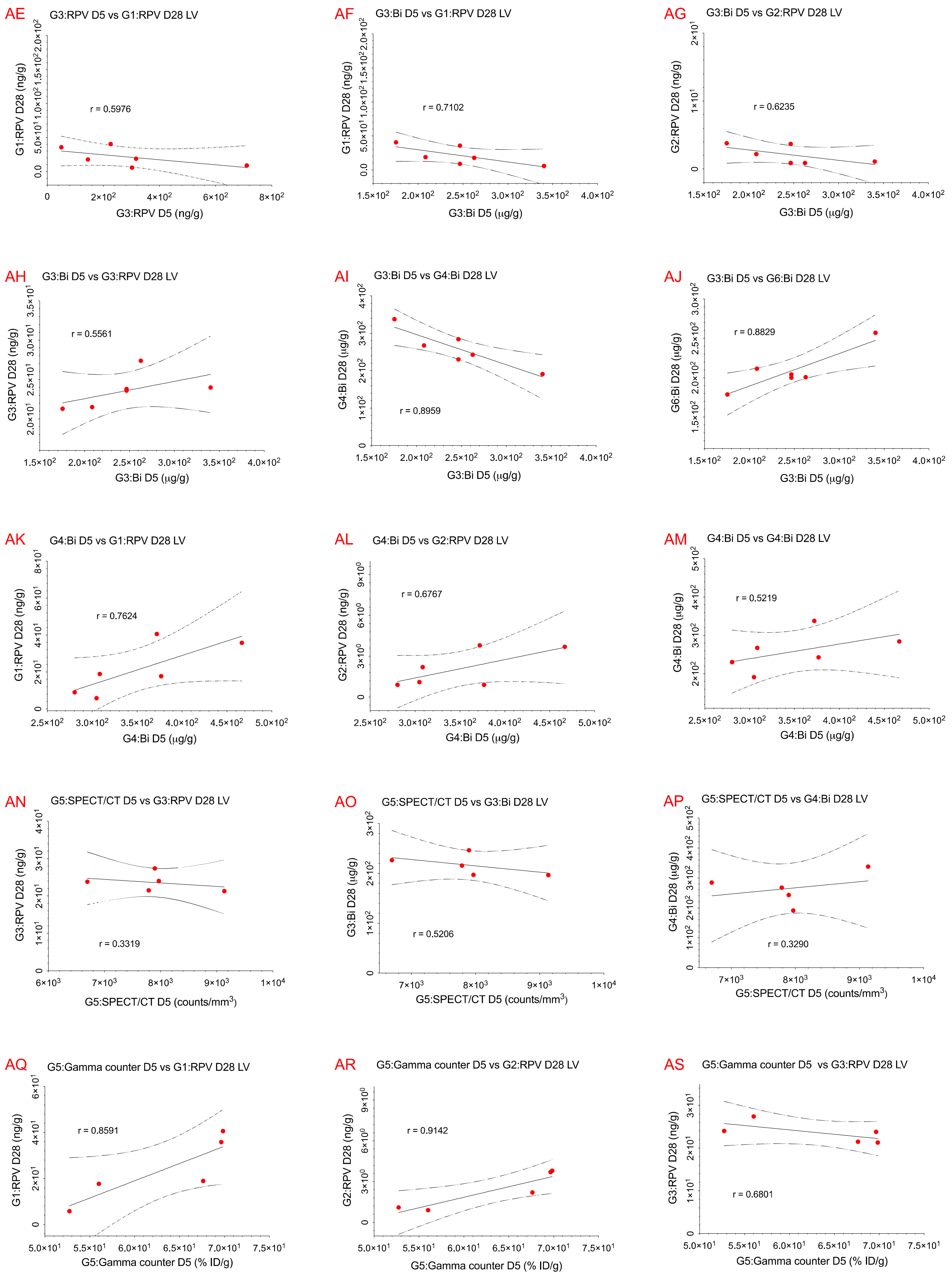
AE
G3:RPV D5 vs G1:RPV D28 LV
r = 0.5976
G1:RPV D28 (ng/g)
G3:RPV D5 (ng/g)
AF
G3:Bi D5 vs G1:RPV D28 LV
r = 0.7102
G1:RPV D28 (ng/g)
G3:Bi D5 (μg/g)
AG
G3:Bi D5 vs G2:RPV D28 LV
r = 0.6235
G2:RPV D28 (ng/g)
G3:Bi D5 (μg/g)
AH
G3:Bi D5 vs G3:RPV D28 LV
r = 0.5561
G3:RPV D28 (ng/g)
G3:Bi D5 (μg/g)
AI
G3:Bi D5 vs G4:Bi D28 LV
r = 0.8959
G4:Bi D28 (μg/g)
G3:Bi D5 (μg/g)
AJ
G3:Bi D5 vs G6:Bi D28 LV
r = 0.8829
G6:Bi D28 (μg/g)
G3:Bi D5 (μg/g)
AK
G4:Bi D5 vs G1:RPV D28 LV
r = 0.7624
G1:RPV D28 (ng/g)
G4:Bi D5 (μg/g)
AL
G4:Bi D5 vs G2:RPV D28 LV
r = 0.6767
G2:RPV D28 (ng/g)
G4:Bi D5 (μg/g)
AM
G4:Bi D5 vs G4:Bi D28 LV
r = 0.5219
G4:Bi D28 (μg/g)
G4:Bi D5 (μg/g)
AN
G5:SPECT/CT D5 vs G3:RPV D28 LV
r = 0.3319
G3:RPV D28 (ng/g)
G5:SPECT/CT D5 (counts/mm3)
AO
G5:SPECT/CT D5 vs G3:Bi D28 LV
r = 0.5206
G3:Bi D28 (μg/g)
G5:SPECT/CT D5 (counts/mm3)
AP
G5:SPECT/CT D5 vs G4:Bi D28 LV
r = 0.3290
G4:Bi D28 (μg/g)
G5:SPECT/CT D5 (counts/mm3)
AQ
G5:Gamma counter D5 vs G1:RPV D28 LV
r = 0.8591
G1:RPV D28 (ng/g)
G5:Gamma counter D5 (% ID/g)
AR
G5:Gamma counter D5 vs G2:RPV D28 LV
r = 0.9142
G2:RPV D28 (ng/g)
G5:Gamma counter D5 (% ID/g)
AS
G5:Gamma counter D5 vs G3:RPV D28 LV
r = 0.6801
G3:RPV D28 (ng/g)
G5:Gamma counter D5 (% ID/g)

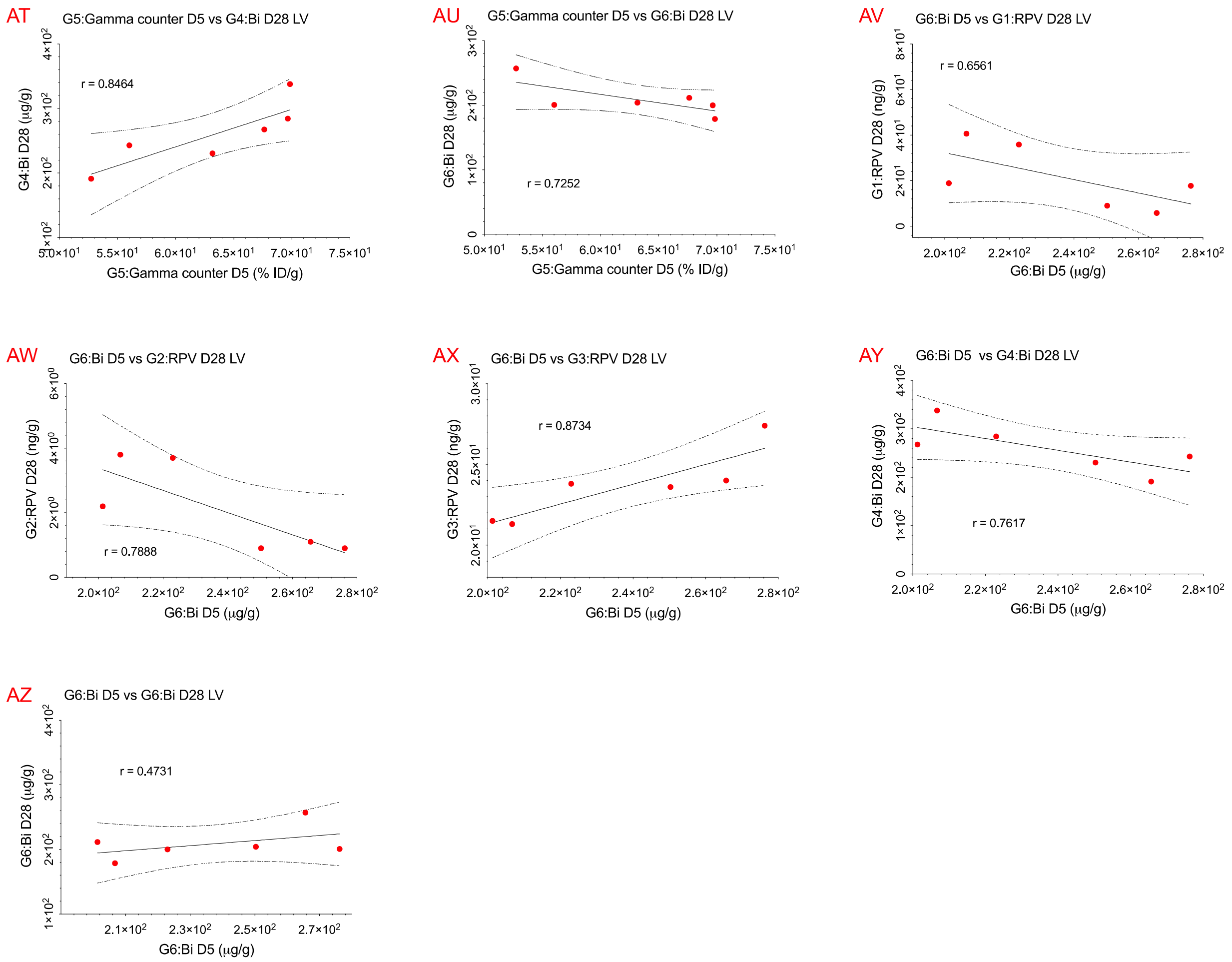

Figure S17. Pearson's correlation analysis of liver tissue. Correlation data were considered from bismuth content (ICP-MS), *ex-vivo* radioactivity (Gamma counter), radioactivity counts per volume of tissue (SPECT/CT ROI), and RPV drug content (UPLC-MS/MS) from days 2, 5 and 28. Only comparisons with a Pearson's coefficient greater than 0.5 (r > 0.5) are shown.

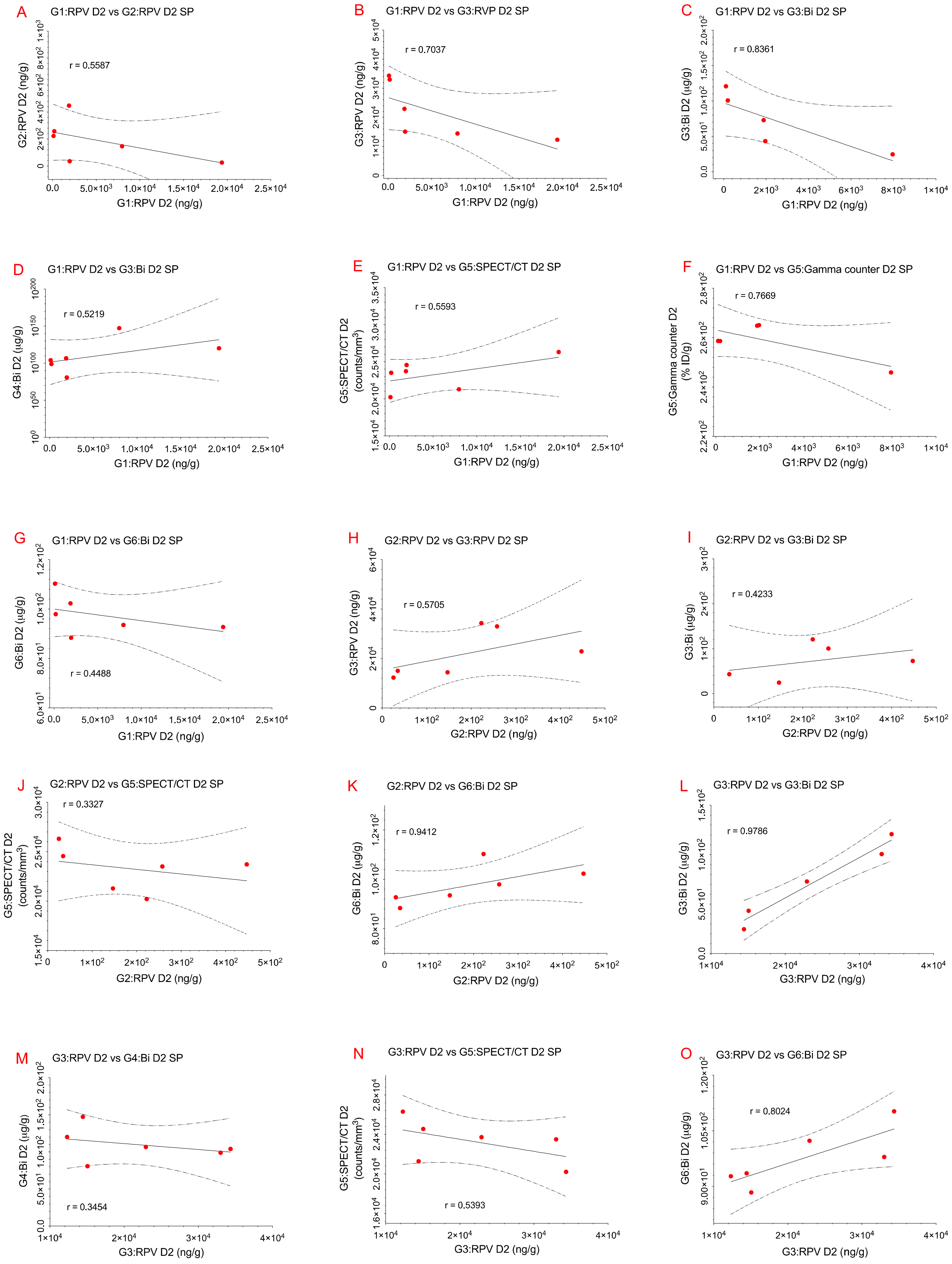
A
G1:RPV D2 vs G2:RPV D2 SP
r = 0.5587
G2:RPV D2 (ng/g)
G1:RPV D2 (ng/g)
B
G1:RPV D2 vs G3:RVP D2 SP
r = 0.7037
G3:RPV D2 (ng/g)
G1:RPV D2 (ng/g)
C
G1:RPV D2 vs G3:Bi D2 SP
r = 0.8361
G3:Bi D2 (µg/g)
G1:RPV D2 (ng/g)
D
G1:RPV D2 vs G3:Bi D2 SP
r = 0.5219
G4:Bi D2 (µg/g)
G1:RPV D2 (ng/g)
E
G1:RPV D2 vs G5:SPECT/CT D2 SP
r = 0.5593
G5:SPECT/CT D2 (counts/mm^3)
G1:RPV D2 (ng/g)
F
G1:RPV D2 vs G5:Gamma counter D2 SP
r = 0.7669
G5:Gamma counter D2 (% ID/g)
G1:RPV D2 (ng/g)
G
G1:RPV D2 vs G6:Bi D2 SP
r = 0.4488
G6:Bi D2 (µg/g)
G1:RPV D2 (ng/g)
H
G2:RPV D2 vs G3:RPV D2 SP
r = 0.5705
G3:RPV D2 (ng/g)
G2:RPV D2 (ng/g)
I
G2:RPV D2 vs G3:Bi D2 SP
r = 0.4233
G3:Bi (µg/g)
G2:RPV D2 (ng/g)
J
G2:RPV D2 vs G5:SPECT/CT D2 SP
r = 0.3327
G5:SPECT/CT D2 (counts/mm^3)
G2:RPV D2 (ng/g)
K
G2:RPV D2 vs G6:Bi D2 SP
r = 0.9412
G6:Bi D2 (µg/g)
G2:RPV D2 (ng/g)
L
G3:RPV D2 vs G3:Bi D2 SP
r = 0.9786
G3:Bi D2 (µg/g)
G3:RPV D2 (ng/g)
M
G3:RPV D2 vs G4:Bi D2 SP
r = 0.3454
G4:Bi D2 (µg/g)
G3:RPV D2 (ng/g)
N
G3:RPV D2 vs G5:SPECT/CT D2 SP
r = 0.5393
G5:SPECT/CT D2 (counts/mm^3)
G3:RPV D2 (ng/g)
O
G3:RPV D2 vs G6:Bi D2 SP
r = 0.8024
G6:Bi D2 (µg/g)
G3:RPV D2 (ng/g)

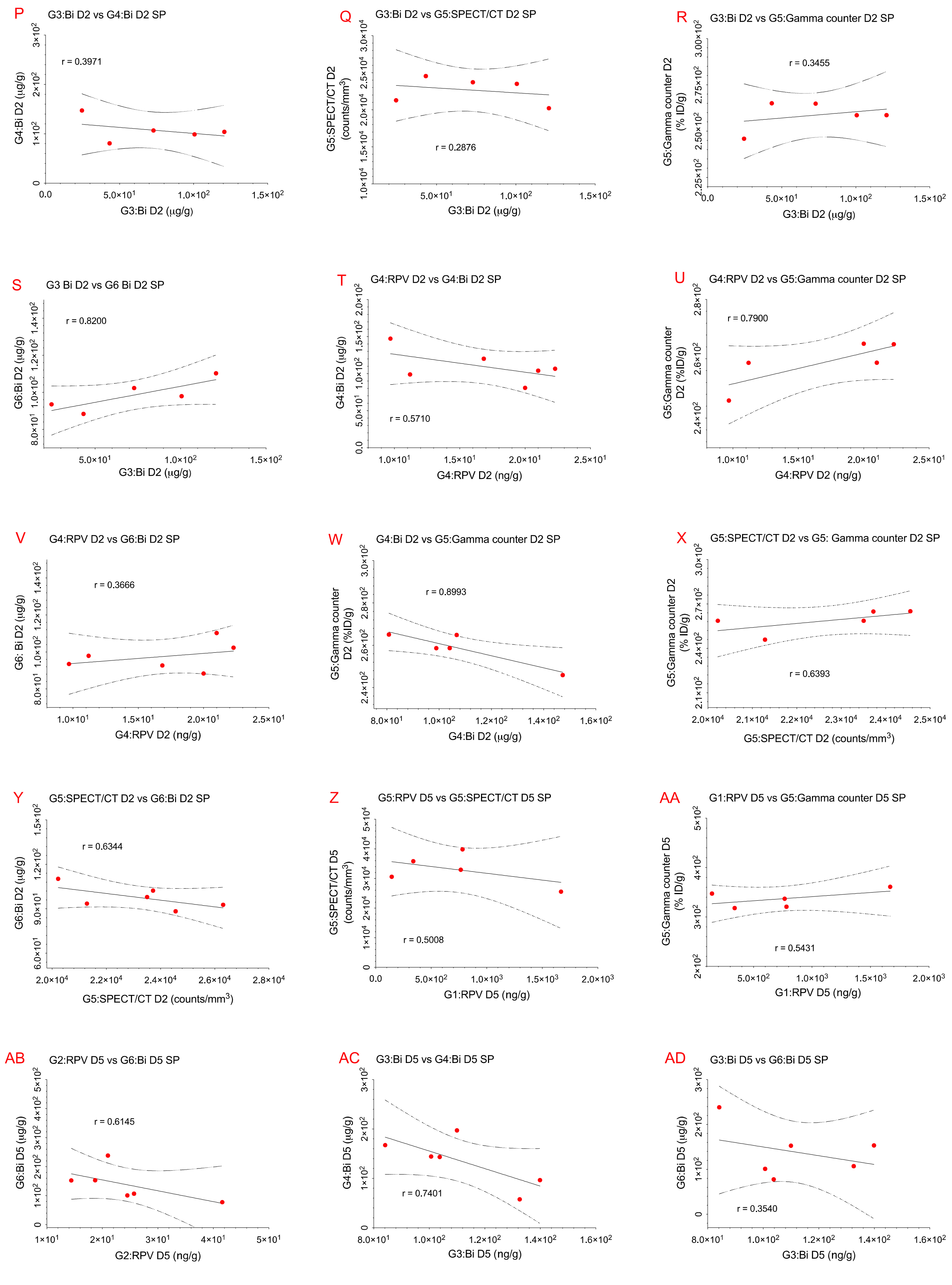
P
G3:Bi D2 vs G4:Bi D2 SP
r = 0.3971
G4:Bi D2 (μg/g)
G3:Bi D2 (μg/g)
Q
G3:Bi D2 vs G5:SPECT/CT D2 SP
r = 0.2876
G5:SPECT/CT D2 (counts/mm3)
G3:Bi D2 (μg/g)
R
G3:Bi D2 vs G5:Gamma counter D2 SP
r = 0.3455
G5:Gamma counter D2 (% ID/g)
G3:Bi D2 (μg/g)
S
G3 Bi D2 vs G6 Bi D2 SP
r = 0.8200
G6:Bi D2 (μg/g)
G3:Bi D2 (μg/g)
T
G4:RPV D2 vs G4:Bi D2 SP
r = 0.5710
G4:Bi D2 (μg/g)
G4:RPV D2 (ng/g)
U
G4:RPV D2 vs G5:Gamma counter D2 SP
r = 0.7900
G5:Gamma counter D2 (%ID/g)
G4:RPV D2 (ng/g)
V
G4:RPV D2 vs G6:Bi D2 SP
r = 0.3666
G6: Bi D2 (μg/g)
G4:RPV D2 (ng/g)
W
G4:Bi D2 vs G5:Gamma counter D2 SP
r = 0.8993
G5:Gamma counter D2 (%ID/g)
G4:Bi D2 (μg/g)
X
G5:SPECT/CT D2 vs G5: Gamma counter D2 SP
r = 0.6393
G5:Gamma counter D2 (% ID/g)
G5:SPECT/CT D2 (counts/mm3)
Y
G5:SPECT/CT D2 vs G6:Bi D2 SP
r = 0.6344
G6:Bi D2 (μg/g)
G5:SPECT/CT D2 (counts/mm3)
Z
G5:RPV D5 vs G5:SPECT/CT D5 SP
r = 0.5008
G5:SPECT/CT D5 (counts/mm3)
G1:RPV D5 (ng/g)
AA
G1:RPV D5 vs G5:Gamma counter D5 SP
r = 0.5431
G5:Gamma counter D5 (% ID/g)
G1:RPV D5 (ng/g)
AB
G2:RPV D5 vs G6:Bi D5 SP
r = 0.6145
G6:Bi D5 (μg/g)
G2:RPV D5 (ng/g)
AC
G3:Bi D5 vs G4:Bi D5 SP
r = 0.7401
G4:Bi D5 (μg/g)
G3:Bi D5 (ng/g)
AD
G3:Bi D5 vs G6:Bi D5 SP
r = 0.3540
G6:Bi D5 (μg/g)
G3:Bi D5 (ng/g)

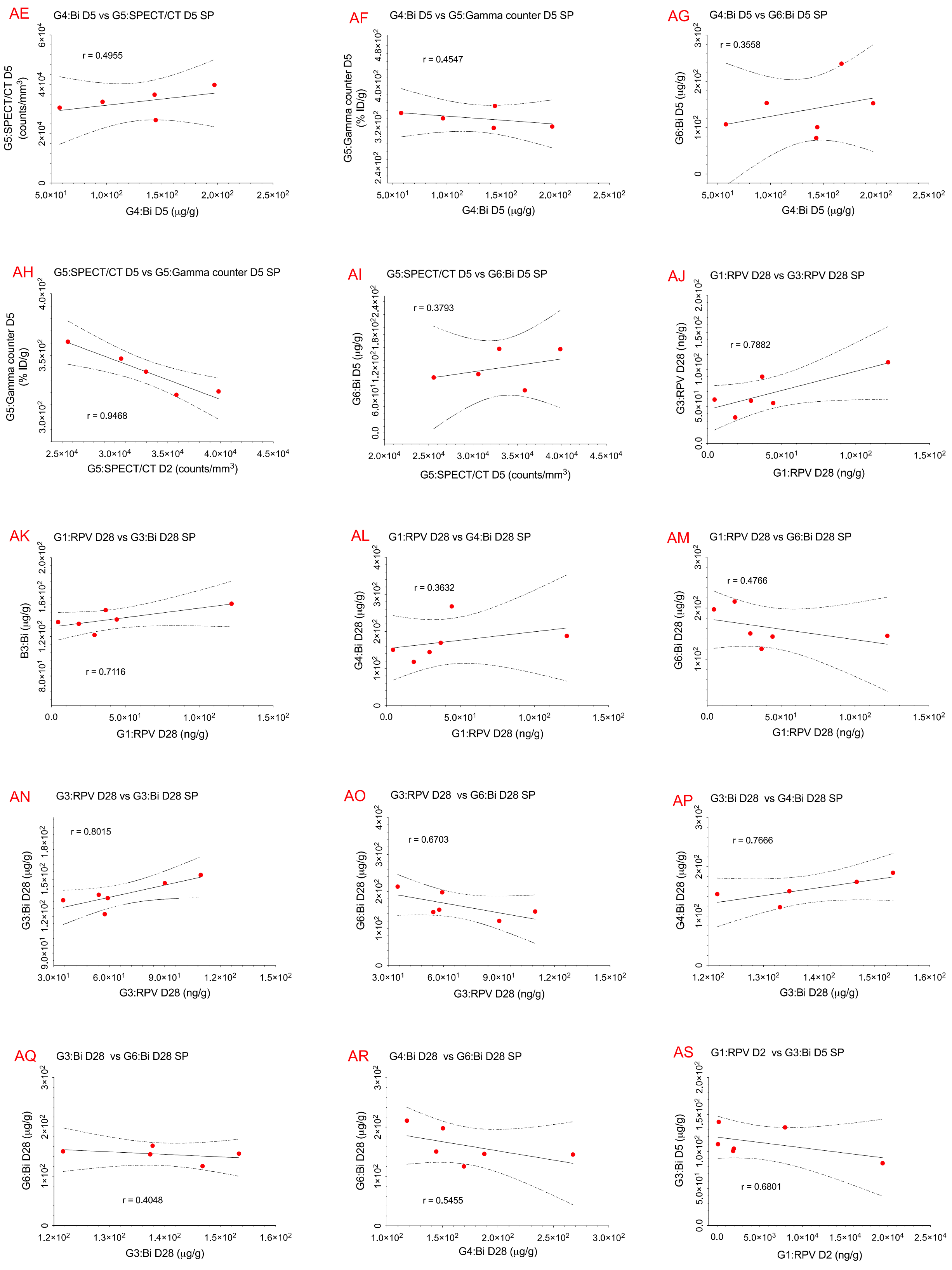
AE
G4:Bi D5 vs G5:SPECT/CT D5 SP
r = 0.4955
G5:SPECT/CT D5 (counts/mm3)
G4:Bi D5 (μg/g)
AF
G4:Bi D5 vs G5:Gamma counter D5 SP
r = 0.4547
G5:Gamma counter D5 (% ID/g)
G4:Bi D5 (μg/g)
AG
G4:Bi D5 vs G6:Bi D5 SP
r = 0.3558
G6:Bi D5 (μg/g)
G4:Bi D5 (μg/g)
AH
G5:SPECT/CT D5 vs G5:Gamma counter D5 SP
r = 0.9468
G5:Gamma counter D5 (% ID/g)
G5:SPECT/CT D2 (counts/mm3)
AI
G5:SPECT/CT D5 vs G6:Bi D5 SP
r = 0.3793
G6:Bi D5 (μg/g)
G5:SPECT/CT D5 (counts/mm3)
AJ
G1:RPV D28 vs G3:RPV D28 SP
r = 0.7882
G3:RPV D28 (ng/g)
G1:RPV D28 (ng/g)
AK
G1:RPV D28 vs G3:Bi D28 SP
r = 0.7116
B3:Bi (μg/g)
G1:RPV D28 (ng/g)
AL
G1:RPV D28 vs G4:Bi D28 SP
r = 0.3632
G4:Bi D28 (μg/g)
G1:RPV D28 (ng/g)
AM
G1:RPV D28 vs G6:Bi D28 SP
r = 0.4766
G6:Bi D28 (μg/g)
G1:RPV D28 (ng/g)
AN
G3:RPV D28 vs G3:Bi D28 SP
r = 0.8015
G3:Bi D28 (μg/g)
G3:RPV D28 (ng/g)
AO
G3:RPV D28 vs G6:Bi D28 SP
r = 0.6703
G6:Bi D28 (μg/g)
G3:RPV D28 (ng/g)
AP
G3:Bi D28 vs G4:Bi D28 SP
r = 0.7666
G4:Bi D28 (μg/g)
G3:Bi D28 (μg/g)
AQ
G3:Bi D28 vs G6:Bi D28 SP
r = 0.4048
G6:Bi D28 (μg/g)
G3:Bi D28 (μg/g)
AR
G4:Bi D28 vs G6:Bi D28 SP
r = 0.5455
G6:Bi D28 (μg/g)
G4:Bi D28 (μg/g)
AS
G1:RPV D2 vs G3:Bi D5 SP
r = 0.6801
G3:Bi D5 (μg/g)
G1:RPV D2 (ng/g)

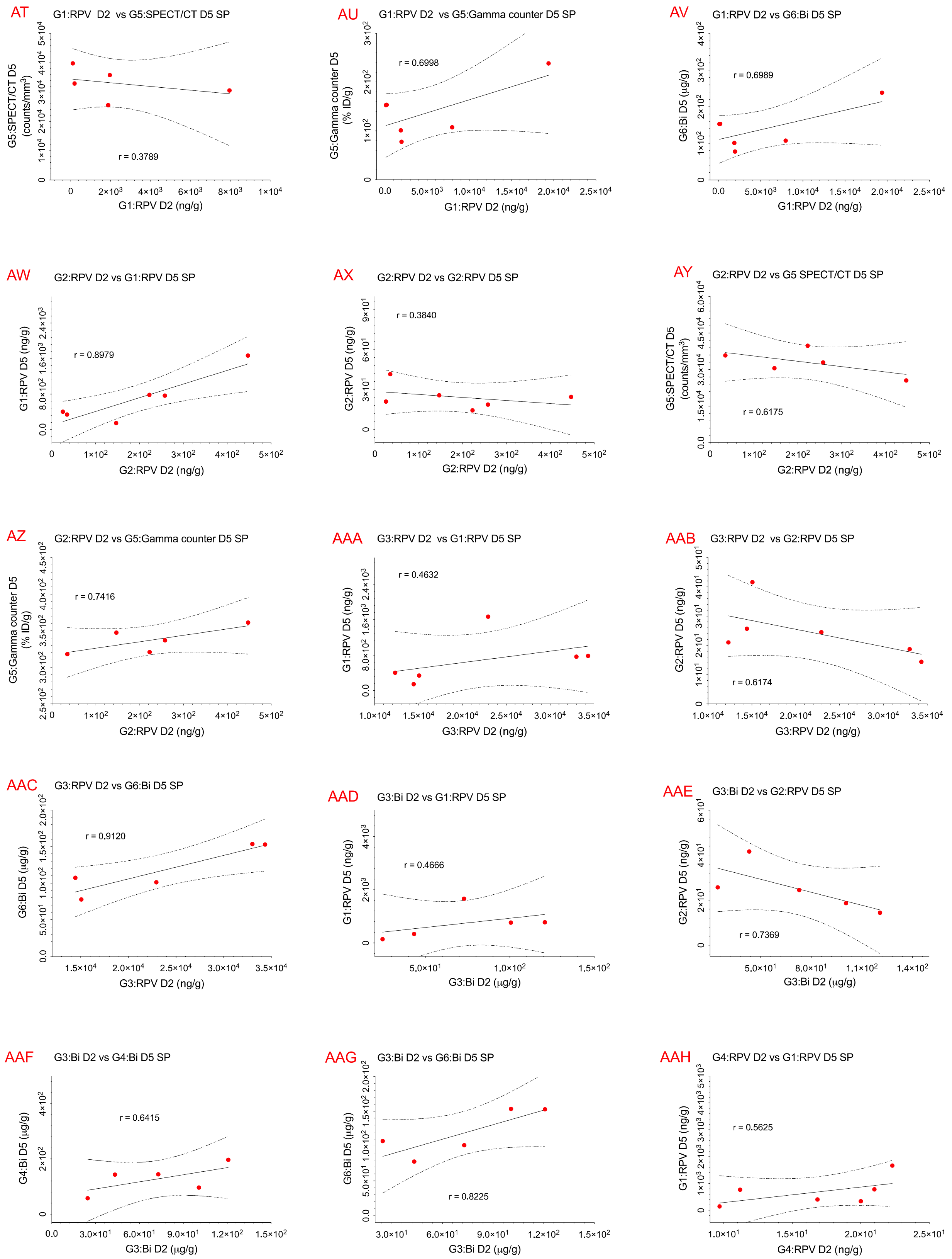
AT
G1:RPV D2 vs G5:SPECT/CT D5 SP
r = 0.3789
G5:SPECT/CT D5 (counts/mm³)
G1:RPV D2 (ng/g)
AU
G1:RPV D2 vs G5:Gamma counter D5 SP
r = 0.6998
G5:Gamma counter D5 (% ID/g)
G1:RPV D2 (ng/g)
AV
G1:RPV D2 vs G6:Bi D5 SP
r = 0.6989
G6:Bi D5 (µg/g)
G1:RPV D2 (ng/g)
AW
G2:RPV D2 vs G1:RPV D5 SP
r = 0.8979
G1:RPV D5 (ng/g)
G2:RPV D2 (ng/g)
AX
G2:RPV D2 vs G2:RPV D5 SP
r = 0.3840
G2:RPV D5 (ng/g)
G2:RPV D2 (ng/g)
AY
G2:RPV D2 vs G5 SPECT/CT D5 SP
r = 0.6175
G5:SPECT/CT D5 (counts/mm³)
G2:RPV D2 (ng/g)
AZ
G2:RPV D2 vs G5:Gamma counter D5 SP
r = 0.7416
G5:Gamma counter D5 (% ID/g)
G2:RPV D2 (ng/g)
AAA
G3:RPV D2 vs G1:RPV D5 SP
r = 0.4632
G1:RPV D5 (ng/g)
G3:RPV D2 (ng/g)
AAB
G3:RPV D2 vs G2:RPV D5 SP
r = 0.6174
G2:RPV D5 (ng/g)
G3:RPV D2 (ng/g)
AAC
G3:RPV D2 vs G6:Bi D5 SP
r = 0.9120
G6:Bi D5 (µg/g)
G3:RPV D2 (ng/g)
AAD
G3:Bi D2 vs G1:RPV D5 SP
r = 0.4666
G1:RPV D5 (ng/g)
G3:Bi D2 (µg/g)
AAE
G3:Bi D2 vs G2:RPV D5 SP
r = 0.7369
G2:RPV D5 (ng/g)
G3:Bi D2 (µg/g)
AAF
G3:Bi D2 vs G4:Bi D5 SP
r = 0.6415
G4:Bi D5 (µg/g)
G3:Bi D2 (µg/g)
AAG
G3:Bi D2 vs G6:Bi D5 SP
r = 0.8225
G6:Bi D5 (µg/g)
G3:Bi D2 (µg/g)
AAH
G4:RPV D2 vs G1:RPV D5 SP
r = 0.5625
G1:RPV D5 (ng/g)
G4:RPV D2 (ng/g)

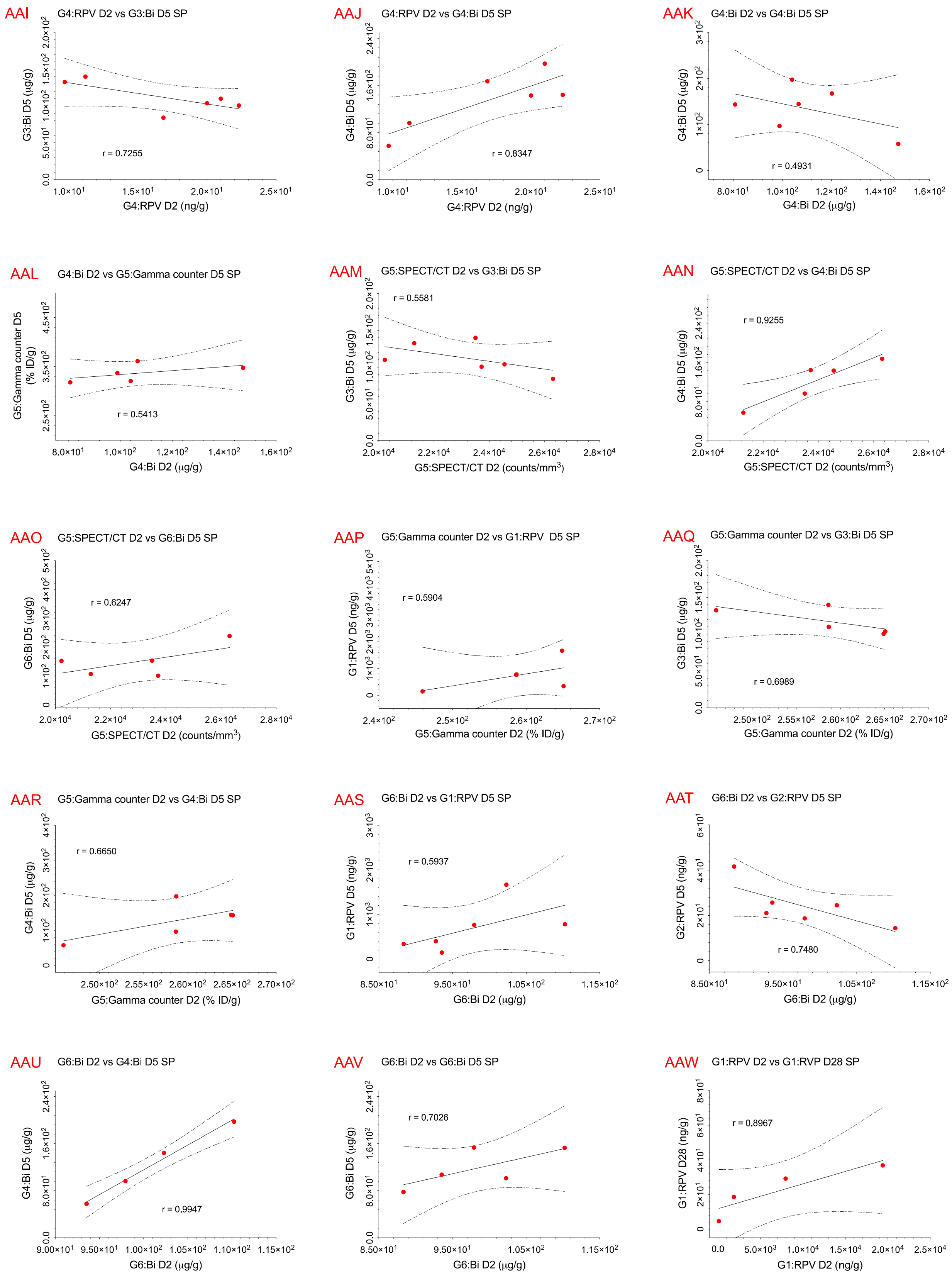
AAI
G4:RPV D2 vs G3:Bi D5 SP
r = 0.7255
G3:Bi D5 (μg/g)
G4:RPV D2 (ng/g)
AAJ
G4:RPV D2 vs G4:Bi D5 SP
r = 0.8347
G4:Bi D5 (μg/g)
G4:RPV D2 (ng/g)
AAK
G4:Bi D2 vs G4:Bi D5 SP
r = 0.4931
G4:Bi D5 (μg/g)
G4:Bi D2 (μg/g)
AAL
G4:Bi D2 vs G5:Gamma counter D5 SP
r = 0.5413
G5:Gamma counter D5 (% ID/g)
G4:Bi D2 (μg/g)
AAM
G5:SPECT/CT D2 vs G3:Bi D5 SP
r = 0.5581
G3:Bi D5 (μg/g)
G5:SPECT/CT D2 (counts/mm3)
AAN
G5:SPECT/CT D2 vs G4:Bi D5 SP
r = 0.9255
G4:Bi D5 (μg/g)
G5:SPECT/CT D2 (counts/mm3)
AAO
G5:SPECT/CT D2 vs G6:Bi D5 SP
r = 0.6247
G6:Bi D5 (μg/g)
G5:SPECT/CT D2 (counts/mm3)
AAP
G5:Gamma counter D2 vs G1:RPV D5 SP
r = 0.5904
G1:RPV D5 (ng/g)
G5:Gamma counter D2 (% ID/g)
AAQ
G5:Gamma counter D2 vs G3:Bi D5 SP
r = 0.6989
G3:Bi D5 (μg/g)
G5:Gamma counter D2 (% ID/g)
AAR
G5:Gamma counter D2 vs G4:Bi D5 SP
r = 0.6650
G4:Bi D5 (μg/g)
G5:Gamma counter D2 (% ID/g)
AAS
G6:Bi D2 vs G1:RPV D5 SP
r = 0.5937
G1:RPV D5 (ng/g)
G6:Bi D2 (μg/g)
AAT
G6:Bi D2 vs G2:RPV D5 SP
r = 0.7480
G2:RPV D5 (ng/g)
G6:Bi D2 (μg/g)
AAU
G6:Bi D2 vs G4:Bi D5 SP
r = 0.9947
G4:Bi D5 (μg/g)
G6:Bi D2 (μg/g)
AAV
G6:Bi D2 vs G6:Bi D5 SP
r = 0.7026
G6:Bi D5 (μg/g)
G6:Bi D2 (μg/g)
AAW
G1:RPV D2 vs G1:RVP D28 SP
r = 0.8967
G1:RPV D28 (ng/g)
G1:RPV D2 (ng/g)

AAX G1:RPV D2 vs G3:RVP D28 SP

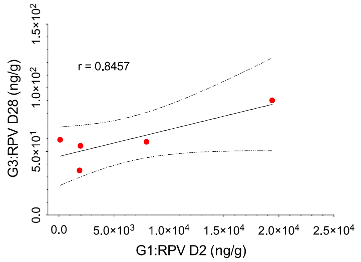

AAY G1:RPV D2 vs G6:Bi D28 SP

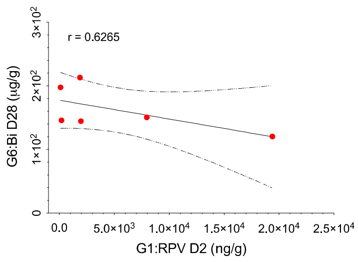

AAZ G2:RPV D2 vs G4:Bi D28 SP

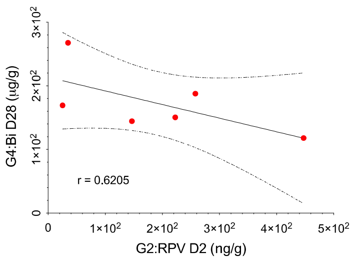

ABA G2:RPV D2 vs G6:Bi D28 SP

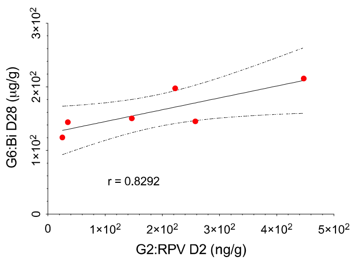

ABB G3:RPV D2 vs G1:RPV D28 SP

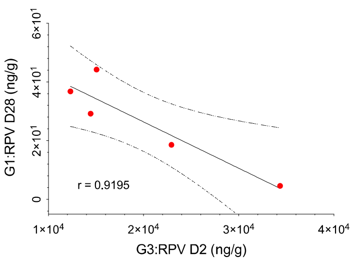

ABC G3:RPV D2 vs G6:Bi D28 SP

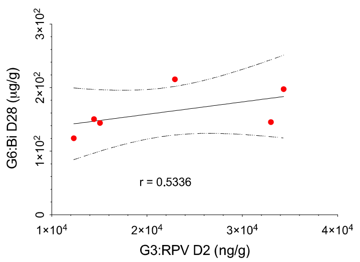

ABD G3:Bi D2 vs G1:RPV D28 SP

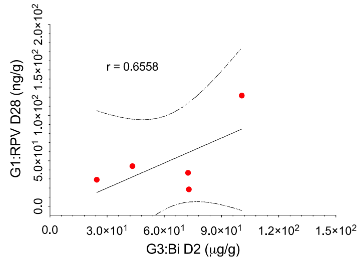

ABE G3:Bi D2 vs G3:RPV D28 SP

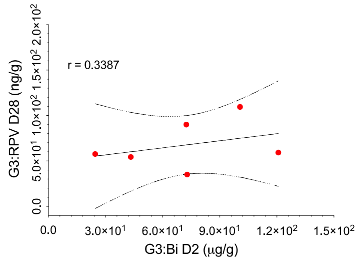

ABF G3:Bi D2 vs G6:Bi D28 SP

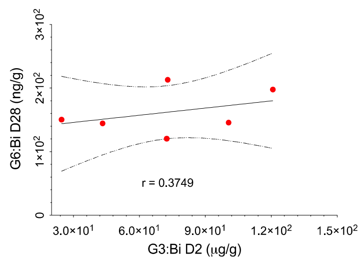

ABG G4:RPV D2 vs G1:RPV D28 SP

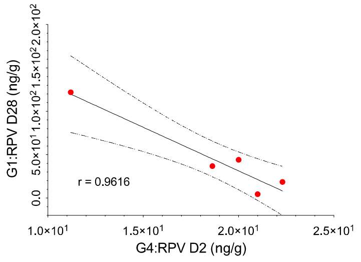

ABH G4:RPV D2 vs G3:RPV D28 SP

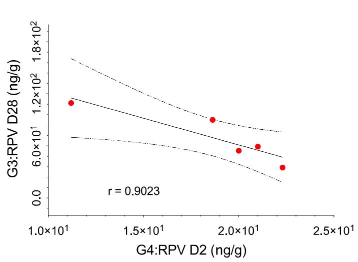

ABI G4:RPV D2 vs G6:Bi D28 SP

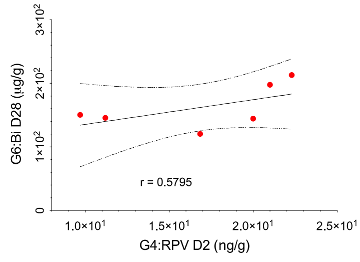

ABJ G4:Bi D2 vs G3:Bi D28 SP

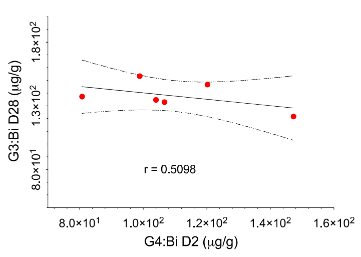

ABK G4:Bi D2 vs G4:Bi D28 SP

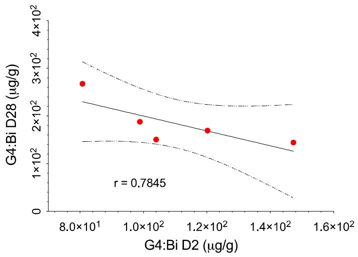

ABL G5:SPECT/CT D2 vs G6:Bi D28 SP

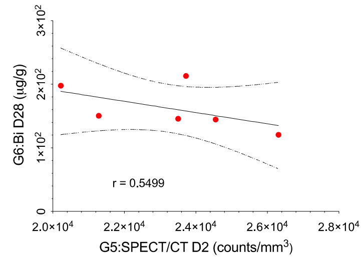

ABM G5:Gamma counter D2 vs G3:Bi D28 SP

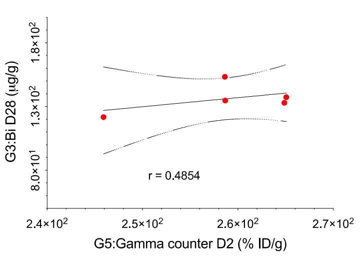

ABN G5:Gamma counter D2 vs G4:Bi D28 SP

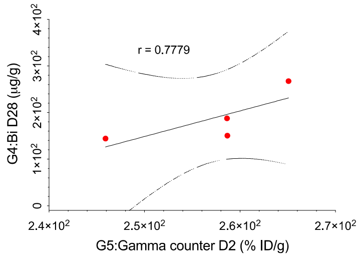

ABO G5:Gamma counter D2 vs G6:Bi D28 SP

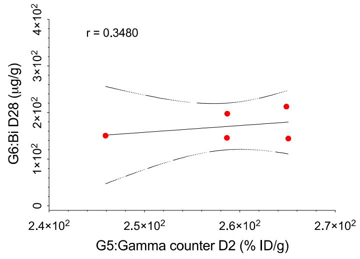

ABP G6:Bi D2 vs G1:RPV D28 SP

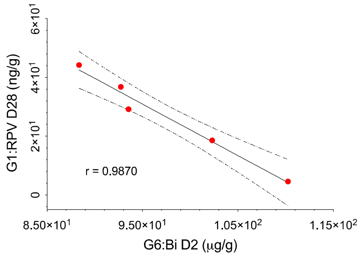

ABQ G6:Bi D2 vs G4:Bi D28 SP

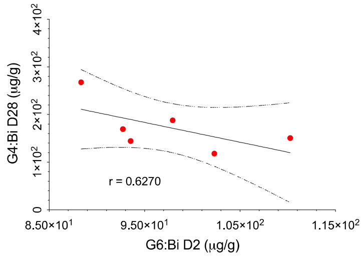

ABR G6:Bi D2 vs G6:Bi D28 SP

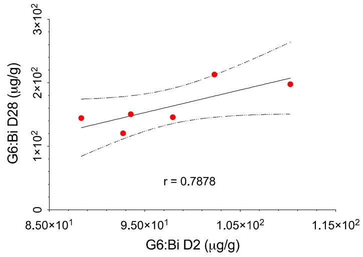

ABS G1:RPV D5 vs G1:RPV D28 SP

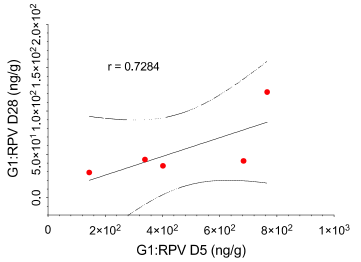

ABT G1:RPV D5 vs G3:RPV D28 SP

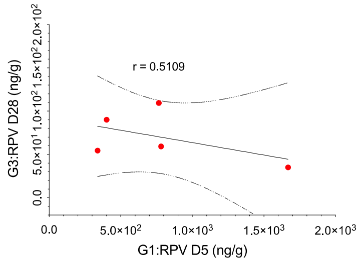

ABU G1:RPV D5 vs G4:Bi D28 SP

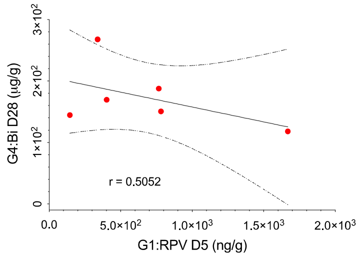

ABV G1:RPV D5 vs G6:Bi D28 SP

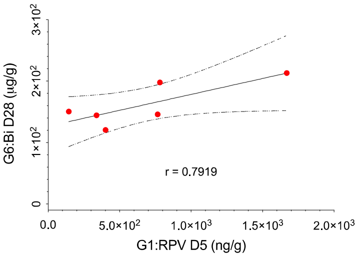

ABW G2:RPV D5 vs G1:RPV D28 SP

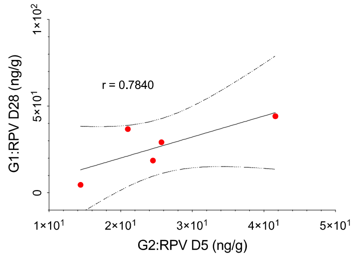

ABX G2:RPV D5 vs G3:RPV D28 SP

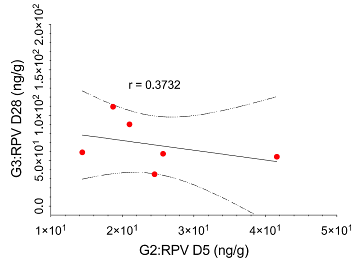

ABY G2:RPV D5 vs G4:Bi D28 SP

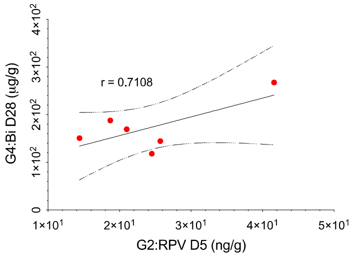

ABZ G2:RPV D5 vs G6:Bi D28 SP

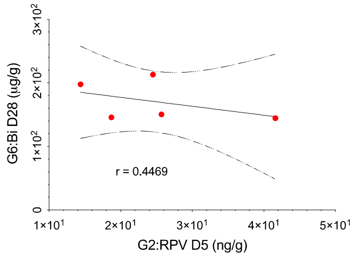

ACA G3:RPV D5 vs G4:Bi D28 SP

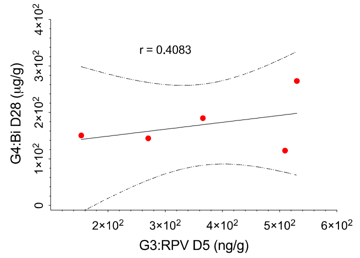

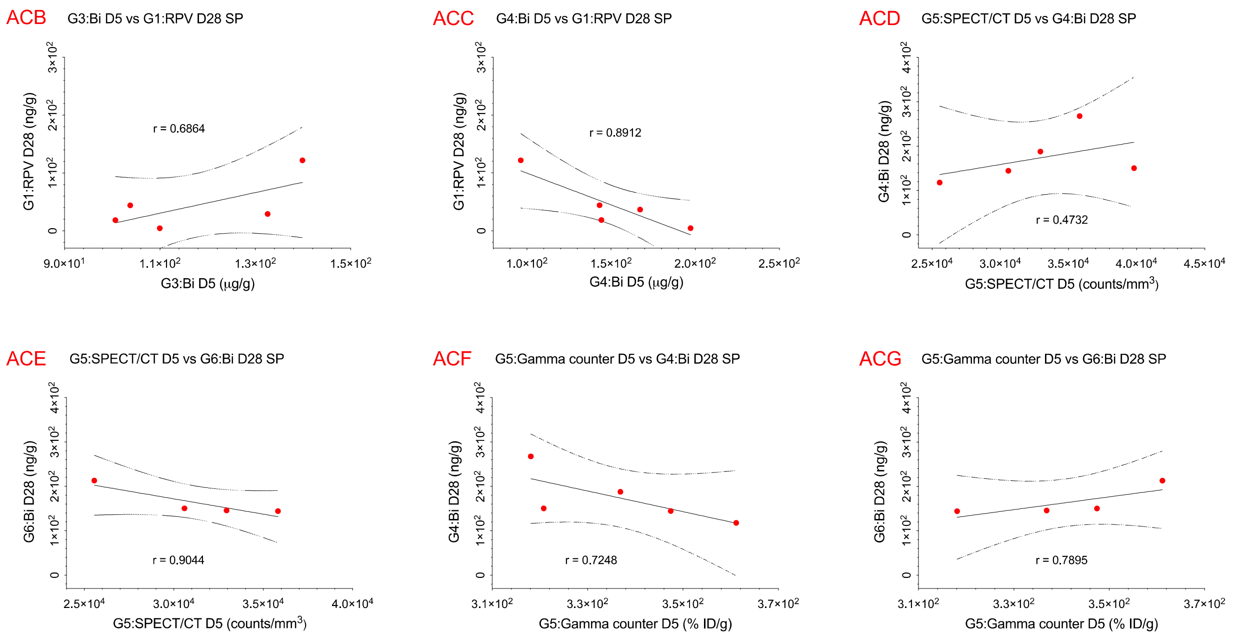

Figure S18. Pearson's correlation analysis of spleen tissue. Correlation data were considered from Bi content (ICP-MS), *ex-vivo* radioactivity (Gamma counter), radioactivity counts per volume of tissue (SPECT/CT, ROI), and RPV drug content (UPLC-MS/MS) from days 2, 5 and 28. Only comparisons with a Pearson's coefficient greater than 0.5 ($r > 0.5$) are shown.

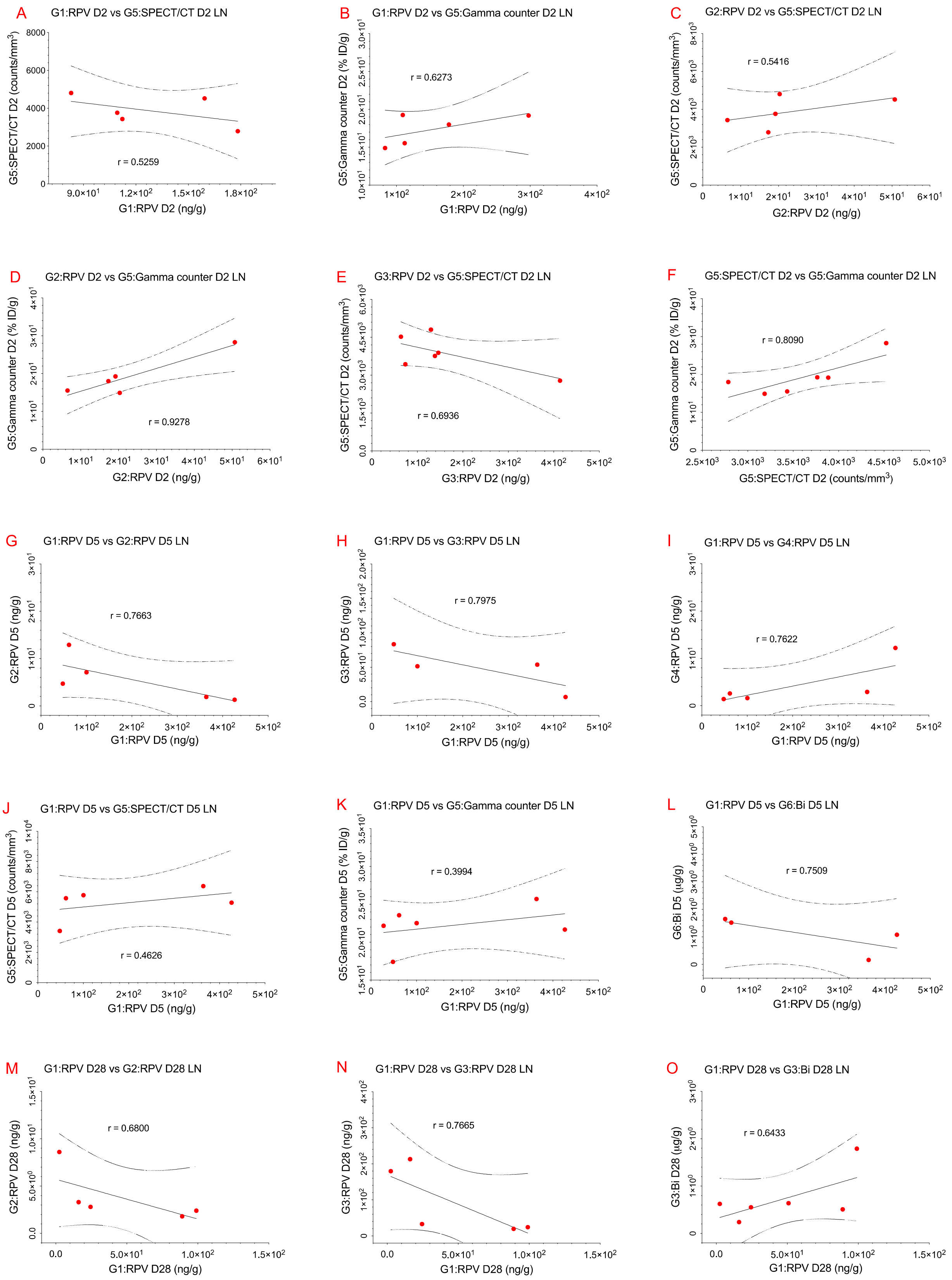
A
G1:RPV D2 vs G5:SPECT/CT D2 LN
r = 0.5259
G5:SPECT/CT D2 (counts/mm^3)
G1:RPV D2 (ng/g)
B
G1:RPV D2 vs G5:Gamma counter D2 LN
r = 0.6273
G5:Gamma counter D2 (% ID/g)
G1:RPV D2 (ng/g)
C
G2:RPV D2 vs G5:SPECT/CT D2 LN
r = 0.5416
G5:SPECT/CT D2 (counts/mm^3)
G2:RPV D2 (ng/g)
D
G2:RPV D2 vs G5:Gamma counter D2 LN
r = 0.9278
G5:Gamma counter D2 (% ID/g)
G2:RPV D2 (ng/g)
E
G3:RPV D2 vs G5:SPECT/CT D2 LN
r = 0.6936
G5:SPECT/CT D2 (counts/mm^3)
G3:RPV D2 (ng/g)
F
G5:SPECT/CT D2 vs G5:Gamma counter D2 LN
r = 0.8090
G5:Gamma counter D2 (% ID/g)
G5:SPECT/CT D2 (counts/mm^3)
G
G1:RPV D5 vs G2:RPV D5 LN
r = 0.7663
G2:RPV D5 (ng/g)
G1:RPV D5 (ng/g)
H
G1:RPV D5 vs G3:RPV D5 LN
r = 0.7975
G3:RPV D5 (ng/g)
G1:RPV D5 (ng/g)
I
G1:RPV D5 vs G4:RPV D5 LN
r = 0.7622
G4:RPV D5 (ng/g)
G1:RPV D5 (ng/g)
J
G1:RPV D5 vs G5:SPECT/CT D5 LN
r = 0.4626
G5:SPECT/CT D5 (counts/mm^3)
G1:RPV D5 (ng/g)
K
G1:RPV D5 vs G5:Gamma counter D5 LN
r = 0.3994
G5:Gamma counter D5 (% ID/g)
G1:RPV D5 (ng/g)
L
G1:RPV D5 vs G6:Bi D5 LN
r = 0.7509
G6:Bi D5 (µg/g)
G1:RPV D5 (ng/g)
M
G1:RPV D28 vs G2:RPV D28 LN
r = 0.6800
G2:RPV D28 (ng/g)
G1:RPV D28 (ng/g)
N
G1:RPV D28 vs G3:RPV D28 LN
r = 0.7665
G3:RPV D28 (ng/g)
G1:RPV D28 (ng/g)
O
G1:RPV D28 vs G3:Bi D28 LN
r = 0.6433
G3:Bi D28 (µg/g)
G1:RPV D28 (ng/g)

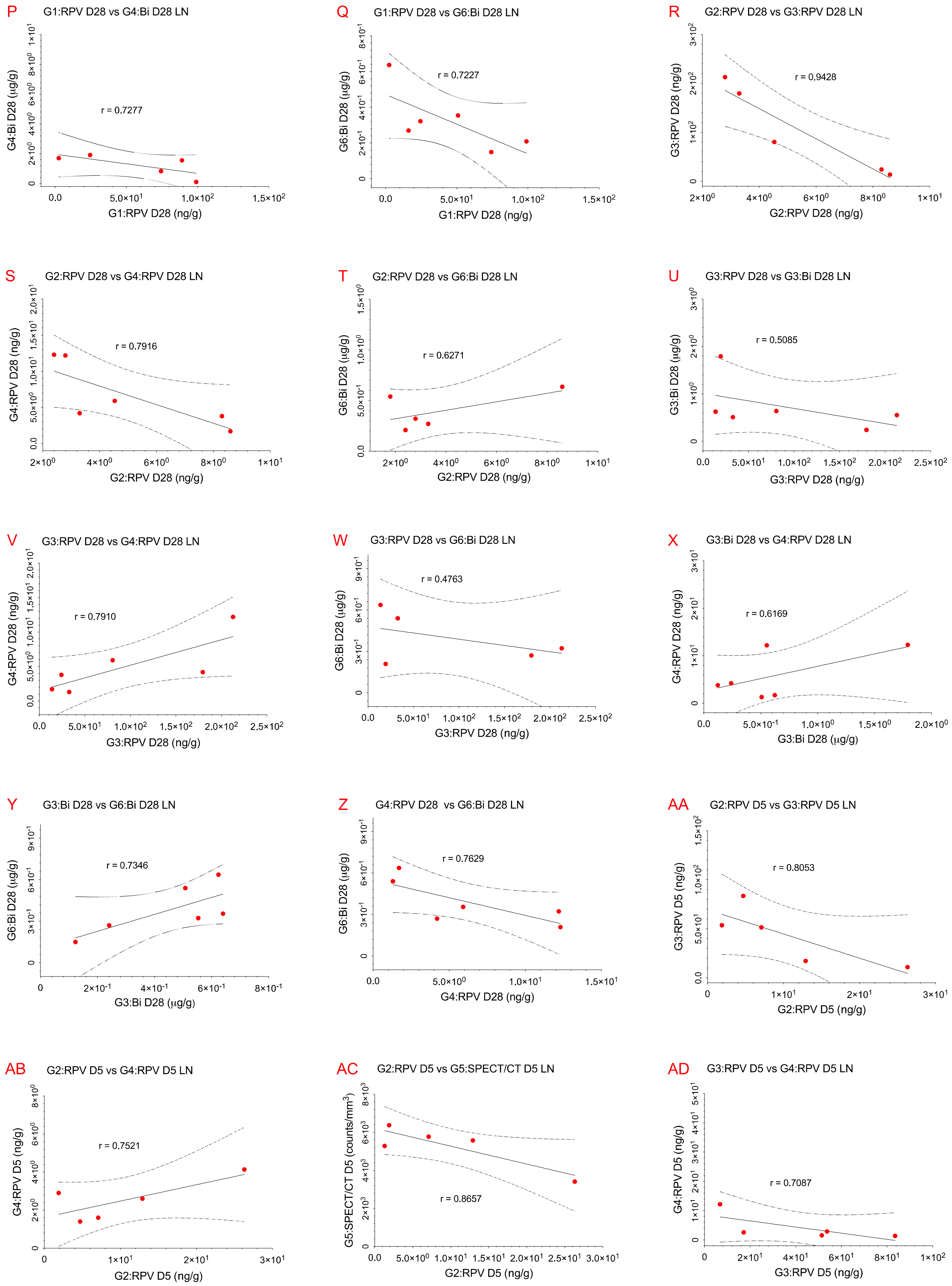

P
G1:RPV D28 vs G4:Bi D28 LN
r = 0.7277
G4:Bi D28 (μg/g)
G1:RPV D28 (ng/g)
Q
G1:RPV D28 vs G6:Bi D28 LN
r = 0.7227
G6:Bi D28 (μg/g)
G1:RPV D28 (ng/g)
R
G2:RPV D28 vs G3:RPV D28 LN
r = 0.9428
G3:RPV D28 (ng/g)
G2:RPV D28 (ng/g)
S
G2:RPV D28 vs G4:RPV D28 LN
r = 0.7916
G4:RPV D28 (ng/g)
G2:RPV D28 (ng/g)
T
G2:RPV D28 vs G6:Bi D28 LN
r = 0.6271
G6:Bi D28 (μg/g)
G2:RPV D28 (ng/g)
U
G3:RPV D28 vs G3:Bi D28 LN
r = 0.5085
G3:Bi D28 (μg/g)
G3:RPV D28 (ng/g)
V
G3:RPV D28 vs G4:RPV D28 LN
r = 0.7910
G4:RPV D28 (ng/g)
G3:RPV D28 (ng/g)
W
G3:RPV D28 vs G6:Bi D28 LN
r = 0.4763
G6:Bi D28 (μg/g)
G3:RPV D28 (ng/g)
X
G3:Bi D28 vs G4:RPV D28 LN
r = 0.6169
G4:RPV D28 (ng/g)
G3:Bi D28 (μg/g)
Y
G3:Bi D28 vs G6:Bi D28 LN
r = 0.7346
G6:Bi D28 (μg/g)
G3:Bi D28 (μg/g)
Z
G4:RPV D28 vs G6:Bi D28 LN
r = 0.7629
G6:Bi D28 (μg/g)
G4:RPV D28 (ng/g)
AA
G2:RPV D5 vs G3:RPV D5 LN
r = 0.8053
G3:RPV D5 (ng/g)
G2:RPV D5 (ng/g)
AB
G2:RPV D5 vs G4:RPV D5 LN
r = 0.7521
G4:RPV D5 (ng/g)
G2:RPV D5 (ng/g)
AC
G2:RPV D5 vs G5:SPECT/CT D5 LN
r = 0.8657
G5:SPECT/CT D5 (counts/mm3)
G2:RPV D5 (ng/g)
AD
G3:RPV D5 vs G4:RPV D5 LN
r = 0.7087
G4:RPV D5 (ng/g)
G3:RPV D5 (ng/g)

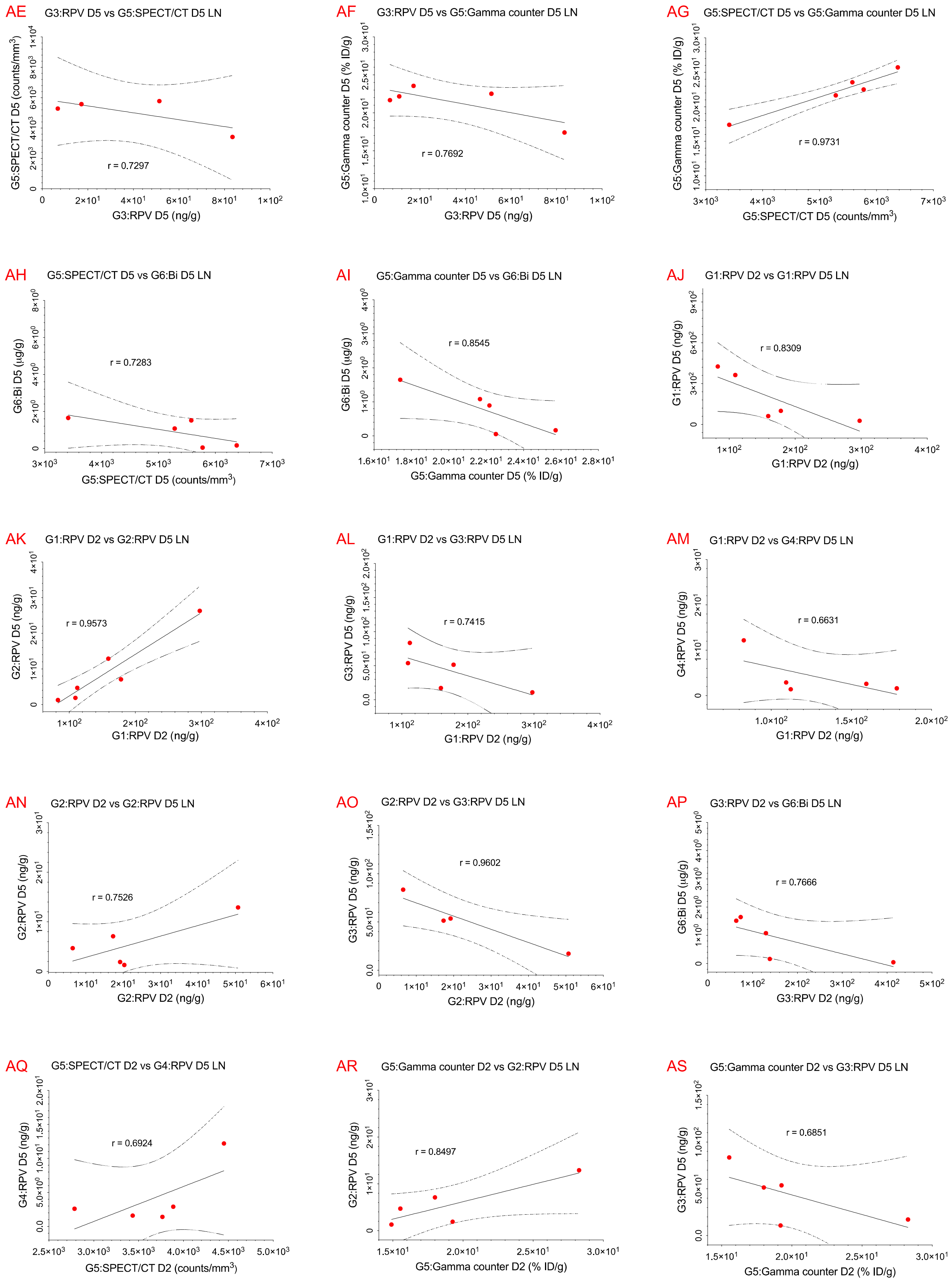
AE
G3:RPV D5 vs G5:SPECT/CT D5 LN
r = 0.7297
G5:SPECT/CT D5 (counts/mm³)
G3:RPV D5 (ng/g)
AF
G3:RPV D5 vs G5:Gamma counter D5 LN
r = 0.7692
G5:Gamma counter D5 (% ID/g)
G3:RPV D5 (ng/g)
AG
G5:SPECT/CT D5 vs G5:Gamma counter D5 LN
r = 0.9731
G5:Gamma counter D5 (% ID/g)
G5:SPECT/CT D5 (counts/mm³)
AH
G5:SPECT/CT D5 vs G6:Bi D5 LN
r = 0.7283
G6:Bi D5 (µg/g)
G5:SPECT/CT D5 (counts/mm³)
AI
G5:Gamma counter D5 vs G6:Bi D5 LN
r = 0.8545
G6:Bi D5 (µg/g)
G5:Gamma counter D5 (% ID/g)
AJ
G1:RPV D2 vs G1:RPV D5 LN
r = 0.8309
G1:RPV D5 (ng/g)
G1:RPV D2 (ng/g)
AK
G1:RPV D2 vs G2:RPV D5 LN
r = 0.9573
G2:RPV D5 (ng/g)
G1:RPV D2 (ng/g)
AL
G1:RPV D2 vs G3:RPV D5 LN
r = 0.7415
G3:RPV D5 (ng/g)
G1:RPV D2 (ng/g)
AM
G1:RPV D2 vs G4:RPV D5 LN
r = 0.6631
G4:RPV D5 (ng/g)
G1:RPV D2 (ng/g)
AN
G2:RPV D2 vs G2:RPV D5 LN
r = 0.7526
G2:RPV D5 (ng/g)
G2:RPV D2 (ng/g)
AO
G2:RPV D2 vs G3:RPV D5 LN
r = 0.9602
G3:RPV D5 (ng/g)
G2:RPV D2 (ng/g)
AP
G3:RPV D2 vs G6:Bi D5 LN
r = 0.7666
G6:Bi D5 (µg/g)
G3:RPV D2 (ng/g)
AQ
G5:SPECT/CT D2 vs G4:RPV D5 LN
r = 0.6924
G4:RPV D5 (ng/g)
G5:SPECT/CT D2 (counts/mm³)
AR
G5:Gamma counter D2 vs G2:RPV D5 LN
r = 0.8497
G2:RPV D5 (ng/g)
G5:Gamma counter D2 (% ID/g)
AS
G5:Gamma counter D2 vs G3:RPV D5 LN
r = 0.6851
G3:RPV D5 (ng/g)
G5:Gamma counter D2 (% ID/g)

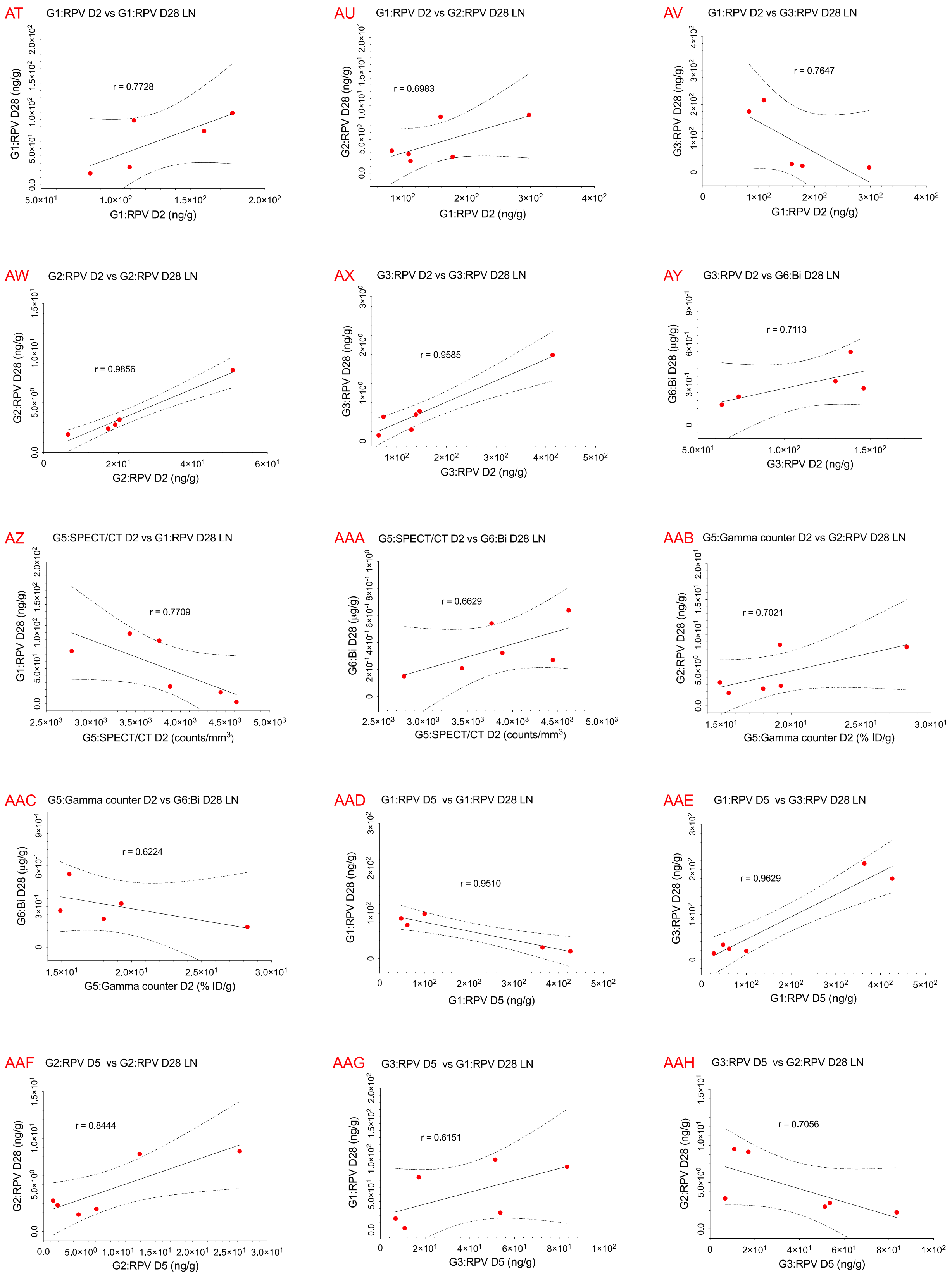

AT
G1:RPV D2 vs G1:RPV D28 LN
r = 0.7728
G1:RPV D28 (ng/g)
G1:RPV D2 (ng/g)
AU
G1:RPV D2 vs G2:RPV D28 LN
r = 0.6983
G2:RPV D28 (ng/g)
G1:RPV D2 (ng/g)
AV
G1:RPV D2 vs G3:RPV D28 LN
r = 0.7647
G3:RPV D28 (ng/g)
G1:RPV D2 (ng/g)
AW
G2:RPV D2 vs G2:RPV D28 LN
r = 0.9856
G2:RPV D28 (ng/g)
G2:RPV D2 (ng/g)
AX
G3:RPV D2 vs G3:RPV D28 LN
r = 0.9585
G3:RPV D28 (ng/g)
G3:RPV D2 (ng/g)
AY
G3:RPV D2 vs G6:Bi D28 LN
r = 0.7113
G6:Bi D28 (μg/g)
G3:RPV D2 (ng/g)
AZ
G5:SPECT/CT D2 vs G1:RPV D28 LN
r = 0.7709
G1:RPV D28 (ng/g)
G5:SPECT/CT D2 (counts/mm3)
AAA
G5:SPECT/CT D2 vs G6:Bi D28 LN
r = 0.6629
G6:Bi D28 (μg/g)
G5:SPECT/CT D2 (counts/mm3)
AAB
G5:Gamma counter D2 vs G2:RPV D28 LN
r = 0.7021
G2:RPV D28 (ng/g)
G5:Gamma counter D2 (% ID/g)
AAC
G5:Gamma counter D2 vs G6:Bi D28 LN
r = 0.6224
G6:Bi D28 (μg/g)
G5:Gamma counter D2 (% ID/g)
AAD
G1:RPV D5 vs G1:RPV D28 LN
r = 0.9510
G1:RPV D28 (ng/g)
G1:RPV D5 (ng/g)
AAE
G1:RPV D5 vs G3:RPV D28 LN
r = 0.9629
G3:RPV D28 (ng/g)
G1:RPV D5 (ng/g)
AAF
G2:RPV D5 vs G2:RPV D28 LN
r = 0.8444
G2:RPV D28 (ng/g)
G2:RPV D5 (ng/g)
AAG
G3:RPV D5 vs G1:RPV D28 LN
r = 0.6151
G1:RPV D28 (ng/g)
G3:RPV D5 (ng/g)
AAH
G3:RPV D5 vs G2:RPV D28 LN
r = 0.7056
G2:RPV D28 (ng/g)
G3:RPV D5 (ng/g)

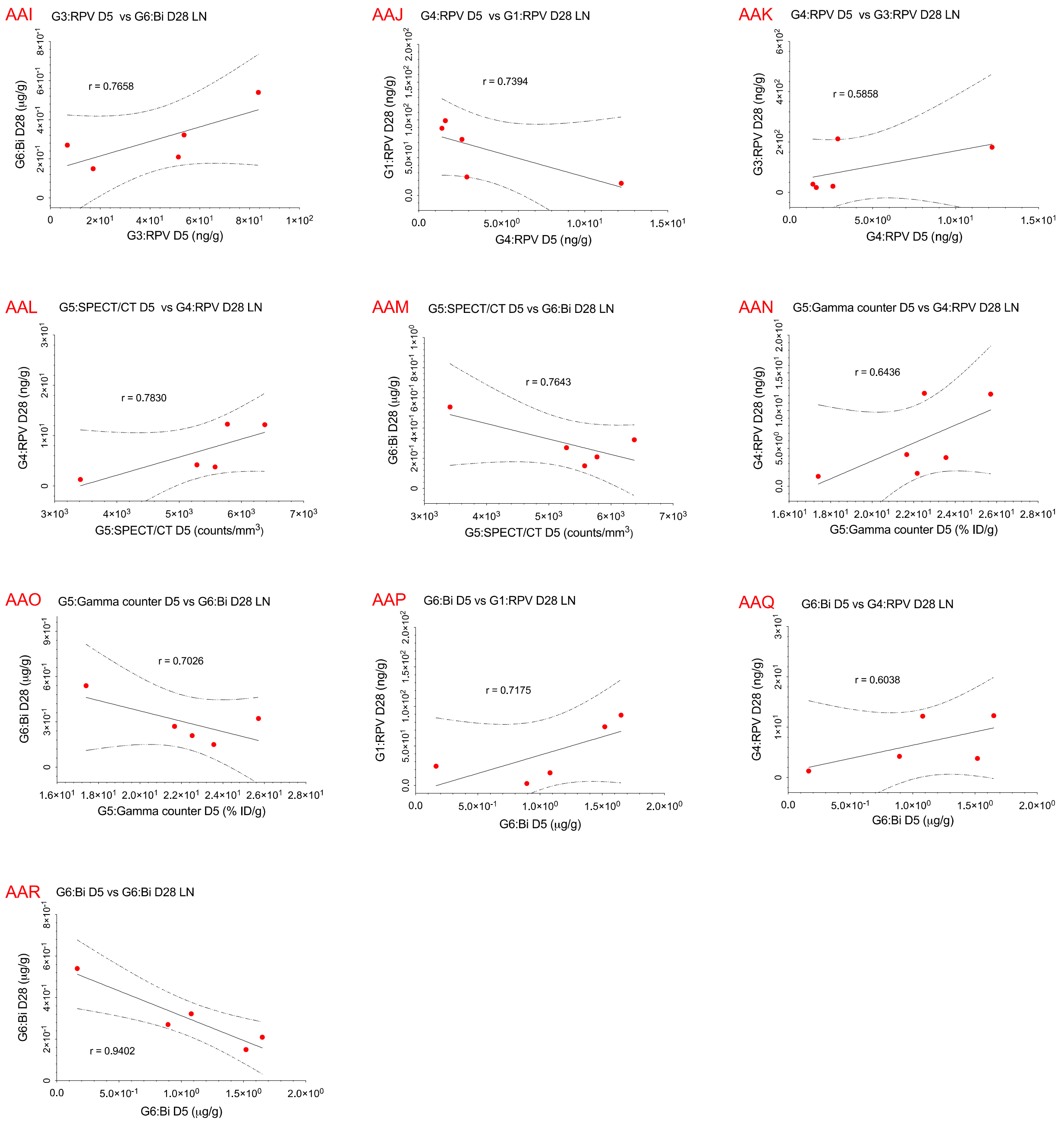

Figure S19. Pearson's correlation analysis of lymph node tissue. Comparison data sets were considered from Bi content (ICP-MS), ex-vivo radioactivity (Gamma counter), radioactivity counts per volume of tissue (SPECT/CT, ROI), and RPV drug content (UPLC-MS/MS) from days 2, 5 and 28. Only comparisons with a Pearson's coefficient greater than 0.5 ($r > 0.5$) are shown.

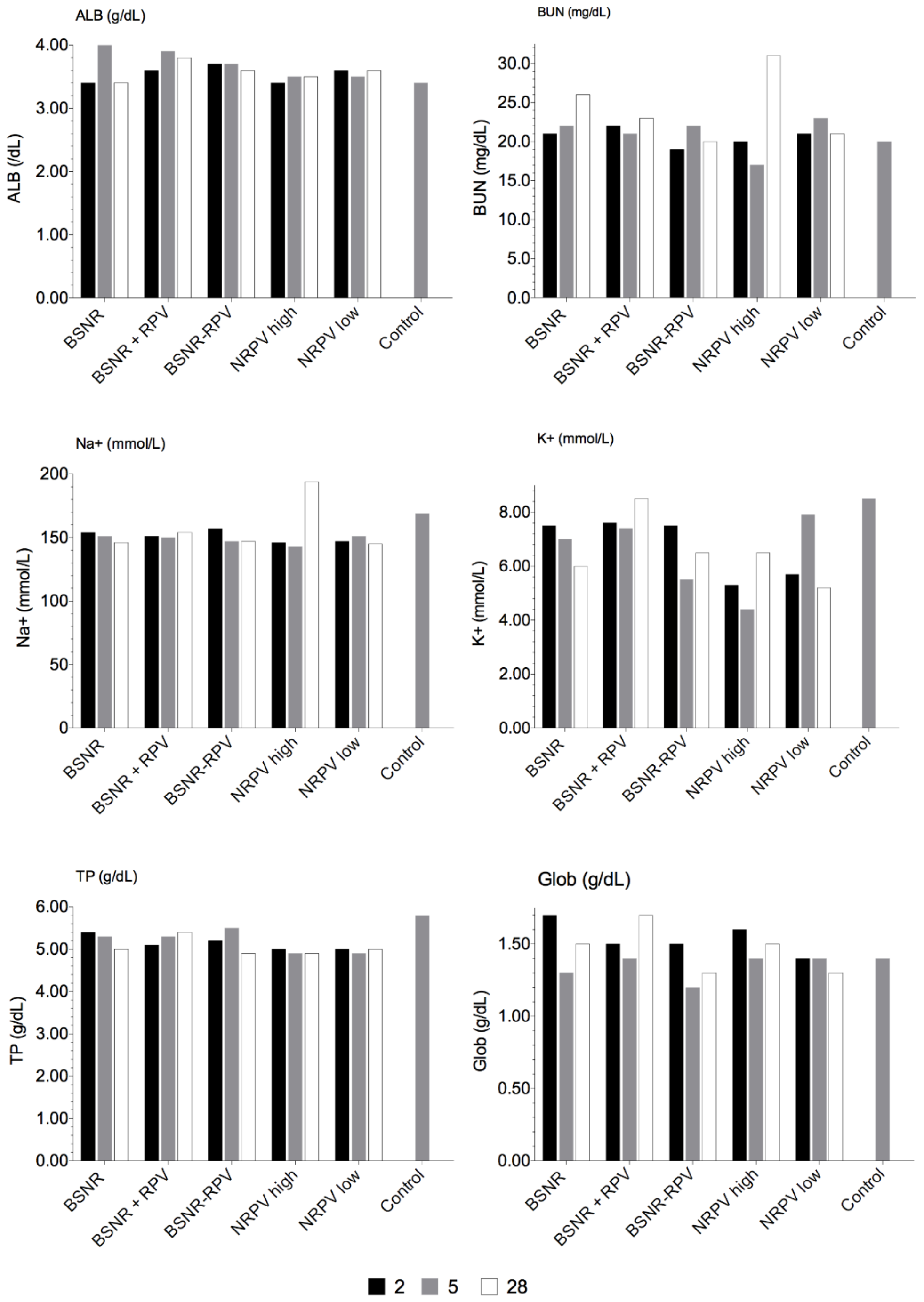

Figure S20. Serum chemistry analysis from treated mice. Comprehensive serum chemistry analysis of mice 2, 5, and 28 days post administration (IV) of NRPV, BSNRs-RPV and BSNRs particles. (ALB: albumin, BUN: blood urea nitrogen, Na+: sodium, K+: potassium, Glob: globulin TP: total protein)

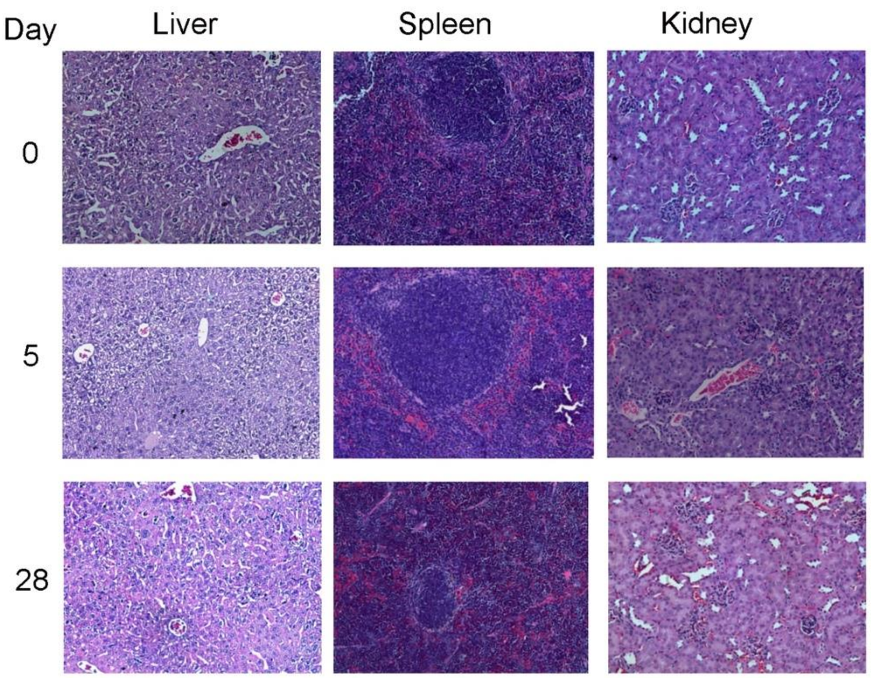

Figure S21. Tissue histopathology of hematoxylin and eosin stained liver, spleen and kidney sections.

References

1. Wang H, Zhu J-J, Zhu J-M, Chen H-Y. Sonochemical Method for the Preparation of Bismuth Sulfide Nanorods. J Phys Chem B. 2002; 106: 3848-54.
2. Pravas Kumar Panigrahi AP. The Growth of Bismuth Sulfide Nanorods from Spherical-Shaped Amorphous Precursor Particles under Hydrothermal Condition. J Nanoparticles. 2013; 2013: 11 pages.
3. Koh YW, Lai CS, Du AY, Tiekink ERT, Loh KP. Growth of Bismuth Sulfide Nanowire Using Bismuth Trisxanthate Single Source Precursors. Chem Mater. 2003; 15: 4544-54.
4. Grigas J, Talik E, Lazauskas V. X-ray Photoelectron Spectra and Electronic Structure of Bi2S3 Crystals. Phys Status Solidi B. 2002; 232: 220-30.
5. Kevadiya BD, Bade AN, Woldstad C, Edagwa BJ, McMillan JM, Sajja BR, et al. Development of europium doped core-shell silica cobalt ferrite functionalized nanoparticles for magnetic resonance imaging. Acta Biomater. 2017; 49: 507-20.
6. Johnstone CD, Lindsay P, Graves EE, Wong E, Perez JR, Poirier Y, et al. Multi-institutional MicroCT image comparison of image-guided small animal irradiators. Phy Med Bio. 2017; 62: 5760-76.
